# Immunopathological signatures in congenital tuberculosis-a case-matched study

**DOI:** 10.3389/fimmu.2026.1614510

**Published:** 2026-03-30

**Authors:** Ren Zhuxiao, Zhang Shandan, Yang Chunhui, Mo Wenhui, Xu Fang, Yang Jie

**Affiliations:** 1 Department of Neonatology, Guangdong Women and Children Hospital, Southern University of Science and Technology, Guangzhou, China; 2 Department of neonatology, Guangdong Neonatal Intensive care unit (ICU) Medical Quality Control Center, Guangzhou, China; 3 Department of Neonatology, The Maternal and Child Health Care Hospital of HuaDu District, Guangdong Medical University, Guangzhou, China; 4 Department of Neonatology, Zhongshan Boai Hospital, Zhongshan, China; 5 Department of Neonatology, Foshan Fosun Chancheng Hospital, Foshan, China; 6 Department of Neonatology, Nanfang Hospital, Southern Medical University, Guangzhou, China

**Keywords:** adaptive response, antigen presentation, congenital tuberculosis, immunopathology, single-cell RNA sequencing

## Abstract

**Introduction:**

Congenital tuberculosis (CTB) is a rare and life-threatening condition with a mortality rate of approximately 53% even with clinical intervention, which is markedly higher than that of adult tuberculosis (ATB). To date, the phenotypic and functional characteristics of immune cells in CTB neonates remain largely uncharacterized.

**Methods:**

In this case-control study, we enrolled nine CTB neonates and nine paired healthy control (pHC) neonates, collecting basic clinical characteristics of the infants and their mothers, and comparing routine blood test results, C-reactive protein (CRP) levels, and lymphocyte subset profiles via flow cytometry during hospitalization. For exploratory mechanistic investigation, we performed single-cell RNA sequencing (scRNA-seq) on peripheral blood mononuclear cells isolated from one pHC neonate and one CTB neonate at the pre-symptomatic early stage, with additional scRNA-seq data of one ATB patient included for comparative analysis.

**Results:**

At the clinical level, CTB neonates exhibited gradual lymphocyte depletion, reduced regulatory T (Treg) cell frequency, and an excessive innate immune response-changes that may drive an overwhelming pro-inflammatory response alongside a compromised adaptive immune response. Exploratory scRNA-seq analysis of the single CTB and pHC neonate revealed a general suppression of immune function in T and natural killer (NK) cells in CTB. T cell subset transcriptomic profiling further showed decreased proportions of Tregs, Th1/17 cells, proliferating T cells, and cytotoxic CD8+ T cells in the CTB neonate, with a concomitant reduction in cytotoxic NK cell frequency. The proportion of myeloid cell subsets in CTB was similar to that in HCs, however, myeloid cells in CTB showed a generally activated inflammatory response. Compared with ATB, myeloid cells of CTB expressed high levels of inflammatory markers. Transcriptomic features of T and NK cell function in the CTB neonate were similar to those in the ATB patient, yet these immune cells in the CTB neonate showed a generally weaker antigen presentation capacity. These preliminary features may partially underpin the high mortality of CTB.

**Discussion:**

Notably, the scRNA-seq findings presented here are exploratory and hypothesis-generating, and should be interpreted with caution. Further studies with expanded scRNA-seq cohorts are urgently needed to validate these initial observations and elucidate the core immune pathophysiological mechanisms of CTB.

## Graphical Abstract

**Figure:**
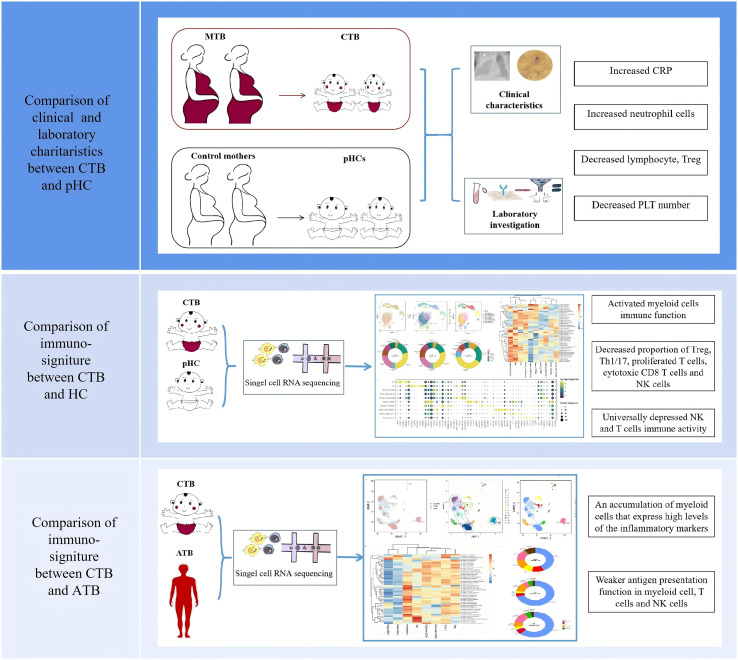
This case-control study included 9 congenital TB (CTB) patients, 9 paired healthy controls (pHC) neonates and one adult TB (ATB) patient. CTB showed a gradual depletion of lymphocytes, depressed T and NK cells function, less Treg cells frequency, as well as excessive innate immune response. These immune cells characters may lead to an overwhelming pro-inflammatory response but weaker specific immune response during CTB infection, which may be one potential factor contributing to the higher mortality of CTB. MTB, maternal tuberculosis; CTB, congenital tuberculosis; ATB, adult tuberculosis; pHC, paired heathy control; NK, natural killer cells; PLT, platelet; Treg, regulatory T cells; CRP, C reactive protein. The abstract should ideally be structured according to the IMRaD format (Introduction, Methods, Results and Discussion). Provide a structured abstract if possible. If your article has been copyedited by us, please provide the updated abstract based on this version.

## 1 Introduction

Tuberculosis (TB) remains the world’s top infectious killer (1), of which 33% comprise female infections (2–4). China is among the countries with a high TB burden; the incidence of TB was reported as 40/100,000 and showed an increasing trend in recent years. Approximately 200,000 pregnant or postpartum women develop TB annually (4). The vertical transmission rate of TB is estimated to be 16%, including all maternal statuses of TB infection (5). Early diagnosis of congenital TB (CTB) remains a major challenge (3–8). The actual number of CTB cases has been substantially underestimated (5–7). To date, approximately 400 CTB cases have been reported in the literature. CTB may cause severe maternal and infant complications, such as premature delivery and neonatal inflammation syndrome, which have been shown to be lethal to neonates, with a mortality rate of approximately 53% even after treatment, being much higher than that of individuals in other age groups infected with TB (3, 5, 8, 9). This stark clinical difference reflects fundamental developmental disparities in immune competence against *Mycobacterium tuberculosis* (M.tb).

The course of TB infection is largely influenced by the host immune system (10). Single-cell genomics studies have revealed the immune cell composition of adult TB (ATB). The immune system in neonates, especially preterm infants, exhibits distinct functions compared with those in older children and adults (11–13). Preterm monocytes exhibited decreased phagocytosis and have decreased upregulation of co-stimulatory molecules necessary for successful antigen presentation to and activation of T and B lymphocytes (11, 12). In addition, T cells in neonates are with limited capacity to activate and differentiate to antigen-specific CD4+ T cells (13). These special characteristics of the immune system in preterm infants may contribute to the high mortality associated with TB infection (10, 14, 15). However, to date, the nature and function of the immune cells of CTB have yet to be reported, and the temporal relationships between innate and adaptive immune responses are unknown.

TB therapy is more effective if patients are identified earlier in the cascade of care when interventions can prevent the deterioration of subsequent diseases (16). However, in patients with CTB, infants often exhibit deteriorated symptoms when a diagnosis is confirmed (3, 9). Thus, it is especially difficult to detect early immune cell characteristics in CTB before a diagnosis is established and the symptoms progress. Compared with active TB infection, when patients present with typical symptoms of TB, the investigation of immune cell characteristics at an early stage of CTB infection could potentially reveal the mechanisms underlying a high mortality and provided candidate immune patterns.

In this study, peripheral blood mononuclear cells (PBMCs) derived from two preterm infants in the control group from our registered randomized controlled trial [autologous cord blood mononuclear cells for prevention of bronchopulmonary dysplasia program (NCT04440670, https://clinicaltrials.gov/)] were sent for single cell RNA-sequencing (scRNA-seq) on the fourth day after birth. Incidentally, we found that one of the infants was diagnosed with CTB 22 days after birth (DAB). Therefore, to investigate the dynamic immune cell characteristics during CTB development, we retrospectively enrolled nine CTB cases and nine paired healthy control (pHC) infants with routine blood tests, lymphocyte flow-cytometric analysis, C-reactive protein (CRP) tests results. CTB is still a understudied condition; integrating well-characterized ATB data allows for identifying age-specific immune signatures that would otherwise be unobservable in a neonatal-only cohort. Therefore, we introduced the online single-cell data of PBMCs derived from one patient with active ATB.

Overall, this study aimed to present the immune signatures of CTB. Our atlas provides valuable data resources and biological insights that could facilitate an improved understanding of the disease and hold potential for guiding early diagnosis and predicting deterioration, pending further validation.

## 2 Materials and methods

### 2.1 Study design

This case-control study included consecutive cases of neonates with CTB and pHC neonates from four level 4 neonatal intensive care units (NICUs) in four tertiary hospitals in Guangdong, China. Written informed consent was obtained from the legal guardians of all participants. Investigators that analyzed the routine blood tests, immune cell subsets, and inflammatory biomarker levels were blinded to the characteristics of patients from whom the blood samples were obtained.

### 2.2 Study approval

The medical ethics committee of Nanfang Hospital, Southern medical university approved the study procedures (ID: NFEC-2022-446). The implementations were in concordance with the International Ethical Guidelines for Research Involving Human Subjects as stated in the Helsinki Declaration.

### 2.3 Study population

**Figure 1:**
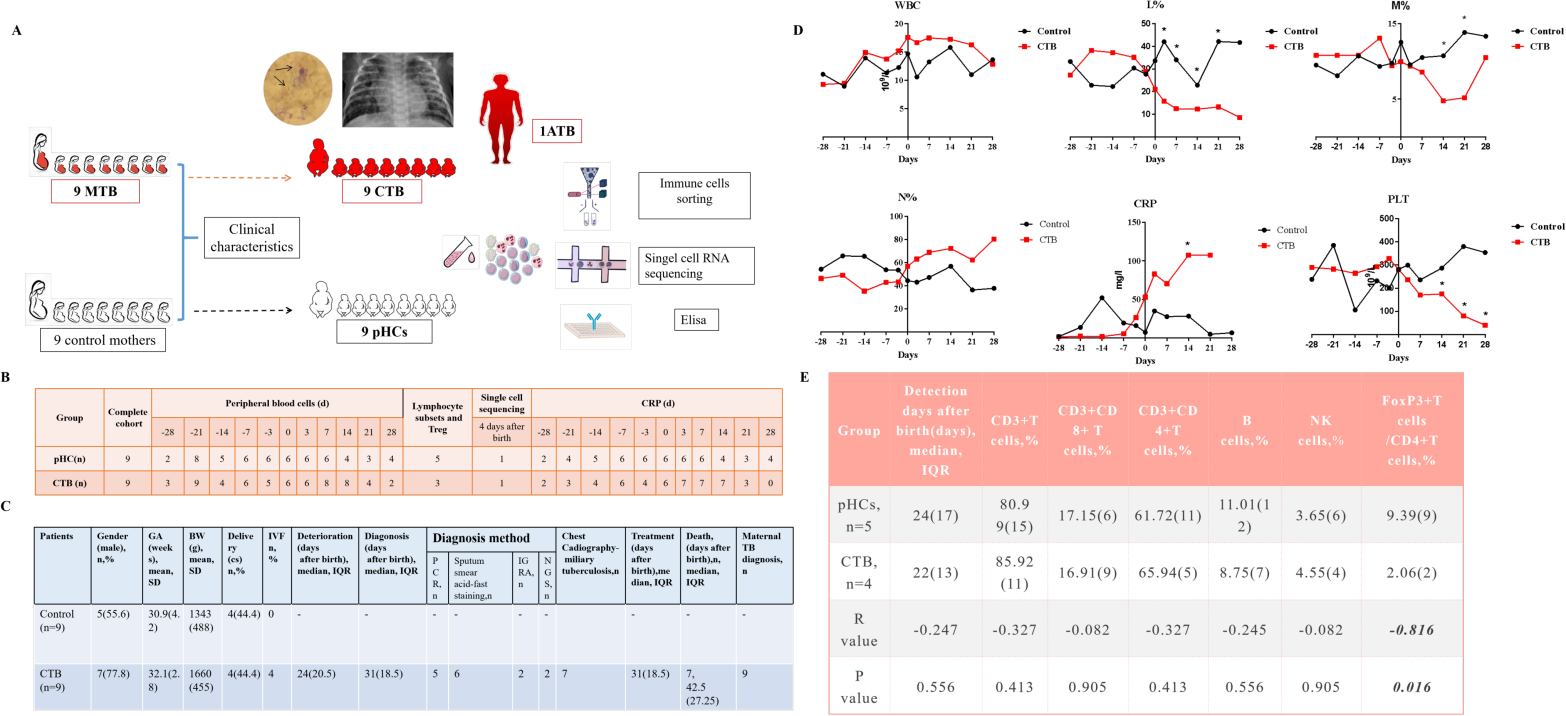
Study design, outline of the detection approach and dynamic immune cell flow-cytometric analysis results. **(A)** Schematic representation of subjects and workflow. **(B)** the number of individuals included in each analysis. **(C)** Demographic and clinical features. **(D)** The dynamic features of PBMC and CRP during TB infection developed, sample size in each time point was showed in **Figure 1B**, median was showed for each time point and non-parametric analysis was used for statistical tests, **(E)** The lymphocytes cell flow-cytometric analysis results. n=4 in CTB and N = 5 in pHC, median and interquartile range (IQR) was showed for each cell type in two groups, Mann-Whitney U test was used for statistical tests, and rank-biserial correlation (r) was used to measure the effect size for Mann-Whitney U test. *P<0.05. MTB, maternal tuberculosis; CTB, congenital tuberculosis; ATB, adult tuberculosis; pHC, paired heathy control.

Abandoned blood samples from route blood tests of two extremely preterm infants enrolled in control group in our registered randomized controlled trial- autologous cord blood mononuclear cells for prevention of bronchopulmonary dysplasia program (NCT04440670, https://clinicaltrials.gov/) on the first week after birth were subjected to performing single-cell RNA-seq. After complement, we found one of them was diagnosed with CTB on 22 days after birth. No symptoms of infection or positive culture pathogen was found in another patient. We therefore compared the single-cell RNA-seq data thus to investigate the immune cell characters of the early CTB infection stage in preterm neonates. Further, open access single-cell RNA-seq result of PBMC from an adult diagnosed with active TB drawn from https://www.ncbi.nlm.nih.gov/sra/?term=SRR11038989 was also introduced to compare with the CTB. To compare the basic characters of CTB, we also enrolled another 8 CTB patients from neonatal intensive care unit (NICUs) from 4 tertiary hospitals who were diagnosed between January, 2015 and May, 2022, and had intact demographics information, routine blood tests results, confirmed diagnosis evidence and chest imaging data. The maternal TB (MTB) diagnosis was also confirmed. 9 healthy control infants without confirmed pathogen infection or congenital malformation were paired with the CTB patients by the following characters: born during similar time duration (± 1 year) and in the same hospital, comparable gestational age (GA: ± 4 weeks), with available routine blood tests results during the similar periods after birth that were comparable to CTB cases (± 7 days). When there were several paired infants, we preferred to choose the infant with more comparable GA and testing time points to the CTB infant and who had available lymphocyte immune cells flow-cytometric analysis result. “No confirmed infection” was defined as no positive blood or cerebral spinal (or the normally sterile body sites) culture. The schematic representation of subject cohorts and work flow was showed **Figures 1A, B**.

The data harmonized across all sites were collected in uniform file where final curation was performed by the clinical research team. CTB was diagnosed as defined by the primary criterion (the presence of proven TB disease) and at least one of the secondary criteria as defined by Cantwell’s criteria (17). These secondary criteria are as follows: (i) lesions in the newborn during the first week of life; (ii) a primary hepatic complex or caseating hepatic granulomata; (iii) TB infection of the placenta or the maternal genital tract; and (iv) exclusion of the possibility of postnatal transmission by investigation of contacts. All of our CTB patients were isolated from their mothers and admitted to the neonatal intensive care unit right after birth. The CTB infection was confirmed by the sputum smear acid-fast staining or culture, positive polymerase chain reaction (PCR) or next generation sequencing (NGS) results for M.tb infection from sputum or blood or a positive interferon gamma release assay (IGRA) result. MTB diagnosis was based on TB infection symptoms and typical chest radiography or computerized tomography results with at least one of the following positive results: IGRA, tuberculin skin test (TST), sputum smear acid-fast staining and culture, PCR or NGS for M.tb from sputum or blood or vaginal secretion. The diagnosis of MTB was all confirmed.

Symptoms of CTB infection deterioration was defined as the following: a.infants who did not need tracheal intubation initially but symptoms deteriorated and needed tracheal intubation or developed shock; b. infants needed reintubation while the symptoms alleviated or were stable previously; c. infants presented as dyspnea who initially did not. Basic clinical demographics information, TB test methods, development and outcomes of CTB and MTB infection characters were presented.

### 2.4 Clinical sample collection and detection

#### 2.4.1 Routine blood tests

The peripheral leucocyte counts, proportion and C reactive protein (CRP) levels in CTB and paired healthy control (pHC) neonates during hospitalization were compared. To compared the leucocyte differential count and proportion characters during CTB developed, the day of CTB symptoms’ deterioration was defined as day 0. The peripheral leucocyte counts and classification between 1-3 days before 0 was defined as -3 point, between 4-7 days was defined as -7 point, between 8-14 was defined as -14 point, between 15 and 21 days was defined as -21 point, and between 22 and 28 days was defined as -28 point. Correspondingly, 1-3 days after 0 was defined as point +3, between 4-7 days was defined as +7 point, between 8-14 was defined as +14 point, between 15 and 21 was defined as +21 point and between 22-28 was defined as +28 point. CRP levels were detected via enzyme linked immunosorbent assay (ELISA) kit (MairuiBiomedical, China).

#### 2.4.2 Lymphocyte immune cells flow-cytometric analysis

Five health control (HC) patients and 3 CTB patients during symptoms deteriorated had available lymphocyte flow cytometry analysis results, among which one of them had twice tests. The flow cytometry analyses were performed after lysis of red blood cell. Proportion of T cells (as well as CD4+ and CD8+ T cells), natural killer cell, B cells in lymphocyte and proportion of CD3/CD4/CD25/FoxP3 (Forkhead box P3) regulatory T cells (Treg) in CD4+ cells were compared. In details, for multi-color lymphocytes flow cytometry analysis, cells were stained with surface antibodies specific for CD45 (Percp, 2D1), CD3/fluorescein isothiocyanate (FITC) (clone SK7), CD4/allophycocyanin (APC) (clone SK3), CD8/phycoerythrin (PE) (clone SK1) for the analysis of T cells subsets in one panel; CD3/fluorescein isothiocyanate (FITC) (clone SK7), CD16/PE (clone B73.1) + CD56/PE (clone NCAM16), and CD19/APC (cloneSJ25C1) for the analysis of B cells and NK cells in one panel. T lymphocytes was marked with CD3+, helper/inducer T lymphocytes was marked with CD3+CD4+, suppressor/cytotoxic T lymphocytes was marked with CD3+CD8+. B cells was marked with CD3-CD19+, natural killer cell was marked with CD3- and CD16+, CD56 +. For regulatory T cells flow cytometry analysis in one panel, cells were firstly stained with surface antibodies specific for CD45 (SV538), CD4/PerCP (clone SK3); CD25/FITC (clone 2A3), followed by intranuclear staining for FoxP3/PE (clone 259D/C7). The dilution antibodies used was 1:50. FoxP3 intranuclear staining was performed according to the manufacturer’s protocol following cell surface staining. The fixed and stained cells were diluted in fluorescence activated cell sorter (FACS) staining buffer (eBioscience, ThermoFisher Scientific, Waltham, MA, USA) and stored directly at 4 °C. Flow cytometric analysis was performed within 24 hours with a BD FACSCanto II cytometer and analyzed with FACS Diva software (BD, Bioscience, San Jose, CA, USA). Lymphocytes were determined by their position in the forward-/side-scatter plot (size/granularity) and co-expression of CD4/CD25/FoxP3 was necessary to identify the Treg cells. For the same patient, the above flow cytometry analyses were performed simultaneously. For different patients, the flow cytometry analyses were performed across different batches. To control the batch effects, the study protocol, the cytometer, the staining buffer and markers used, the gating strategy, the analyses software and the operating personnel were the same for the analyses. The representative raw flow cytometry figure data and gating strategy was showed in **Supplementary File 1**.

### 2.5 10 ×genomics single-cell sample processing and cDNA library preparation

PBMCs were isolated from whole blood through a histopaque density gradient. Cell viability was assessed by trypan blue staining and the samples (cell viability >90%) were prepared and loaded on a 10 X Genomics GemCode Single-cell instrument that generates single-cell Gel Bead-In-EMlusion (GEMs). Full-length, barcoded cDNAs were then amplified by PCR to generate sufficient mass for library construction. Libraries were generated and sequenced from the cDNAs with Chromium Next GEM Single Cell 3’ Reagent Kits v3 according to the manufacturer’s instructions. Single-cell libraries were sequenced on NovaSeq 6000 system (Illumina, USA). Raw sequencing reads are available at the National Genomics Data Center (https://ngdc.cncb.ac.cn/gsa-human/) under the accession numbers HRA015988.

### 2.6 Single-cell data processing, gene expression quantification and cell-type determination

For each sample, the cleaned data filtered for the low quality reads and unrelated sequences were imported to Cell Ranger (10x Genomics, version 2.2.0) and aligned to human reference genome (Ensembl_release 106). Cells were sorted according to the barcodes and the unique molecular identifiers (UMIs) were counted per gene for each cell. Cells with unusually high number of UMIs (≥25000) or mitochondrial gene percent (≥10%) were filtered out. We also excluded cells with less than 500 or more than 4000 genes detected. Additionally, doublet GEMs also should be filtered out. It was achieved by using the tool Doublet Finder (v2.0.3) by the generation of artificial doublets, using the PC distance to find each cell’s proportion of artificial k nearest neighbors (pANN) and ranking them according to the expected number of doublets. Seraut software was used to cluster and group the filtered cells, and R Harmony software was used to merge data and for batch effect correction. Cell type identification was performed using SingleR software.

After scaling the data, dimension reduction was performed using principal component analysis, cell clustering was performed using a graph-based method, and visualization was achieved with uniform manifold approximation and projection (UMAP) considering the top 50 principal components in Seurat. Then, to identify the differently expressed genes (DEGs) and marker genes under a particular condition, we selected the UMAP results as the final visualization of the 22 cell clusters in Seurat. Typically, cell types within a cluster can be directly identified based on canonical cell types using canonical marker genes that strongly indicate which clusters represent the corresponding cell types. For novel clusters or those clusters that lacked canonical marker genes, we used the DEGs as marker genes for cell type identification. We compared gene expression in cells belonging to each cluster with that in all other cells to obtain markers for each cluster. Specifically, the DEGs were identified by comparing cells in a particular cluster with all cells of the other 21 clusters. Then, using the graph-based cluster method, the unsupervised cell cluster result was acquired, and the marker genes were calculated by the FindAlIMarkers function under the following criteria: |log2FC|>0.5, adjusted P-value <0.05 (Ben-jamini-Hochberg correction for multiple testing), and pct>0.25. All marker genes within any particular cluster had to be in the top of the up-regulated or down-regulated genes in that cluster.

### 2.7 KEGG pathway enrichment analysis

Genes were ranked using the moderated T statistics for the relevant coefficient from the limma voom model. Enriched gene sets were identified using the pre-ranked GSEA algorithm implemented in the fgsea R package. Gene set lists were used for kyoto encyclopedia of genes and genomes (KEGG) enrichment assessment. The significance of DEG was analyzed by Model-based Analysis of Single-cell Transcriptomics (MAST) following the criteria including |log2FC|≥1, p_value_adjust ≤ 0.05. P values were adjusted using the Benjamini–Hochberg method for the whole gene set list.

Selected pathways shown in figures were manually curated to select gene sets relevant to immunology and often enriched in several cell types across the various differential expression comparisons. The normalized enrichment score was determined for pathway in each cluster.

### 2.8 Gene set variation analysis

Pathway analyses were performed on the 50 hallmark pathways annotated in the molecular signature database, which was exported using the GSEA Base package (version 2.2.4). The GSVA package (version 1.30.0) was applied with default settings to assign pathway activity estimates to individual cells^19^. Briefly, the activity scores for each cell were compared using a generalized linear model (GLM). The outputs of these GLMs were visualized in heat maps to assess differential activities of pathways between sets of cells.

### 2.9 Trajectory analysis

Destiny software package (v.2.6.2) was used to perform the trajectory analysis based on dimensionality reduction using diffusion maps. We used diffusion maps as a nonlinear dimensionality reduction technique to find the major non-linear components of variation across T cells. We computed diffusion components in each cell type separately using the Charlotte Python package, which implements diffusion maps. To account for differences in cell density and cluster size, we used a fixed perplexity Gaussian kernel with perplexity 30, with symmetric Markov normalization and t=1 diffusion steps. We selected t=1 diffusion steps because this approximates diffusion of information for each cell through its 20 nearest neighbors in our data. To avoid technical biases in cell type proportions and focus on processes explaining variation specifically within major cell types, we performed diffusion map analyses separately on all cells labeled as T cells.

### 2.10 External single-cell RNA sequencing data of active ATB

Single-cell data from the cohort of Yi Cai et al. were downloaded from https://www.ncbi.nlm.nih.gov/sra/?term=SRR11038989 (10x genomics single-cell RNA-seq of Homo sapiens: TB infection adult male peripheral blood dataset-Name: PBMC_TB_3) (18). Using the pre-annotated cell clusters from the original publication, single-cell gene expression data were pooled into pseudo-bulk libraries, and differential gene expression and GSEAs of patients with adult tuberculosis (ATB) versus CTB were done.

The UMAP plots before and after integration, and analysis scripts of all the scRNA-seq in this study are available in **Supplementary File 2**.

### 2.11 Statistical analysis

As this was an observational study, no statistical method was used to predetermine sample size. Means (standard deviation) and Student’s t-test were used for continuous variables with normal distribution, and median and interquartile range (IQR) and non-parametric analysis was used for data with non-normal distribution. The variables’ distribution characteristics were estimated with Kolmogrov-Smirnov test. The number and percentage were reported for categorical variables. Group comparisons of categorical variables were performed using the Fisher’s exact test, or chi-square test, as appropriate. For continuous variables, the mean values at each time point in each group for variables were derived from a generalized linear model and differences in values at each time point were compared using an non-parametric analysis between the two groups for data with non-normal distribution and Student’s t-test were used for data with normal distribution, since the small n and non-normal distribution of the values, median was showed for each time point and non-parametric analysis was used for statistical tests. For the time-anchored blood data, analyses were performed using linear mixed-effects models with patient ID as a random intercept to account for intra-individual correlation in repeated measurements. Each time bin contains only one measurement per infant. No repeated-measures methods were applied. Given the exploratory nature of this analysis and small n, we did not apply formal multiple testing correction (e.g., Bonferroni), as this would overly inflate Type II error. All statistical analyses were performed in GraphPad Prism (version 5.0). Two-tailed statistical tests were conducted and a P <0.05 was considered statistically significant.

## 3 Results

### 3.1 Clinical characteristics of patients with congenital tuberculosis and paired healthy controls

We included nine neonates with confirmed CTB and nine pHC infants between January 2015 and May 2022 from four NICUs in four tertiary hospitals in Guangdong, China (**Figure 1A**). The demographic, clinical, and laboratory detection characteristics of the patients with CTB and their mothers are showed in **Figures 1B, C**; **Supplementary Tables 1**, **2**. The median deterioration time was 24 DAB, and the median diagnosis time 31 DAB. None of the infants received preventive anti-TB therapy. All neonates received anti-TB treatment after diagnosis, and the median number of days of treatment was 31 DAB. A total of 77.7% (7/9) of the patients died at a median age of 42.5 days. The clinical characteristics including gender, GA, birth weight, delivery mode, Apgar scores, respiratory support condition, sepsis evaluations and BPD severity were comparable in two groups, except that infants in CTB group received longer antibiotic therapy (P = 0.001, **Supplementary Table 2**). DAB for which routine blood tests were conducted in the patients with CTB and pHC infants are shown in **Supplementary Table 3**.

### 3.2 Consecutive detection of routine blood tests, C-reactive protein levels, and lymphocyte flow-cytometric analysis

Routine blood tests during hospitalization in the two groups were compared, and the results shown in **Figure 1D**. Significant time × group interactions were detected for L% (β=-0.849, P<0.001), platelet count (PLT; β=-6.570, P = 0.002), monocyte percentage (MN%; β=-0.190, P = 0.001), CRP (β=2.886, P<0.001), and N% (β=1.067, P<0.001), demonstrating distinct temporal trajectories between CTB and pHC neonates. Specifically, compared with pHC, lymphocytes, monocyte and platelet count (PLT) in CTB showed a downward trend over time. On the contrary, the proportion of neutrophil cells and CRP levels showed an upward trend and increased before the symptoms worsened. No significant time × group interaction was found for WBC (β=0.0743, P = 0.333). The de-identified raw longitudinal complete blood counts were available in **Supplementary Table 5**.

As T cells were the major cell type identified in lymphocytes, we also collected the lymphocyte immunophenotype of five HCs and three patients with CTB (one of which had two repeated results recorded at different time points) with available results (**Supplementary Table 5**). We found the total T, B, and natural killer (NK) cell frequencies were comparable between the two groups, but the FoxP3^+^ regulatory T cell (Treg) frequency in CTB neonates (2.06%) was lower than that in pHC neonates (9.39%; p = 0.016) (**Figure 1E**). The de-identified raw flow cytometry percentages were available in **Supplementary Table 5**.

### 3.3 Single-cell RNA sequencing revealed distinct immunopathological characteristics in congenital tuberculosis

For this pilot, hypothesis-generating scRNA-seq study, we analyzed peripheral blood mononuclear cells (PBMCs) from one CTB neonate, one paired healthy control (pHC) neonate, and one adult tuberculosis (ATB) patient, representing single biological replicates per group. We initially isolated and sequenced 24,273 cells from PBMC suspensions derived from two infants: one with CTB (3,597 cells) and one paired control (9,787 cells), as well as one patient with active ATB (10,878 cells). After removing 12.92%, 13.22%, and 15.73% of the cells that represented doublets, empty droplets, and low-quality cells, respectively, we selected 21,421 cells for further analysis (**Supplementary Table 6**).

**Figure 2:**
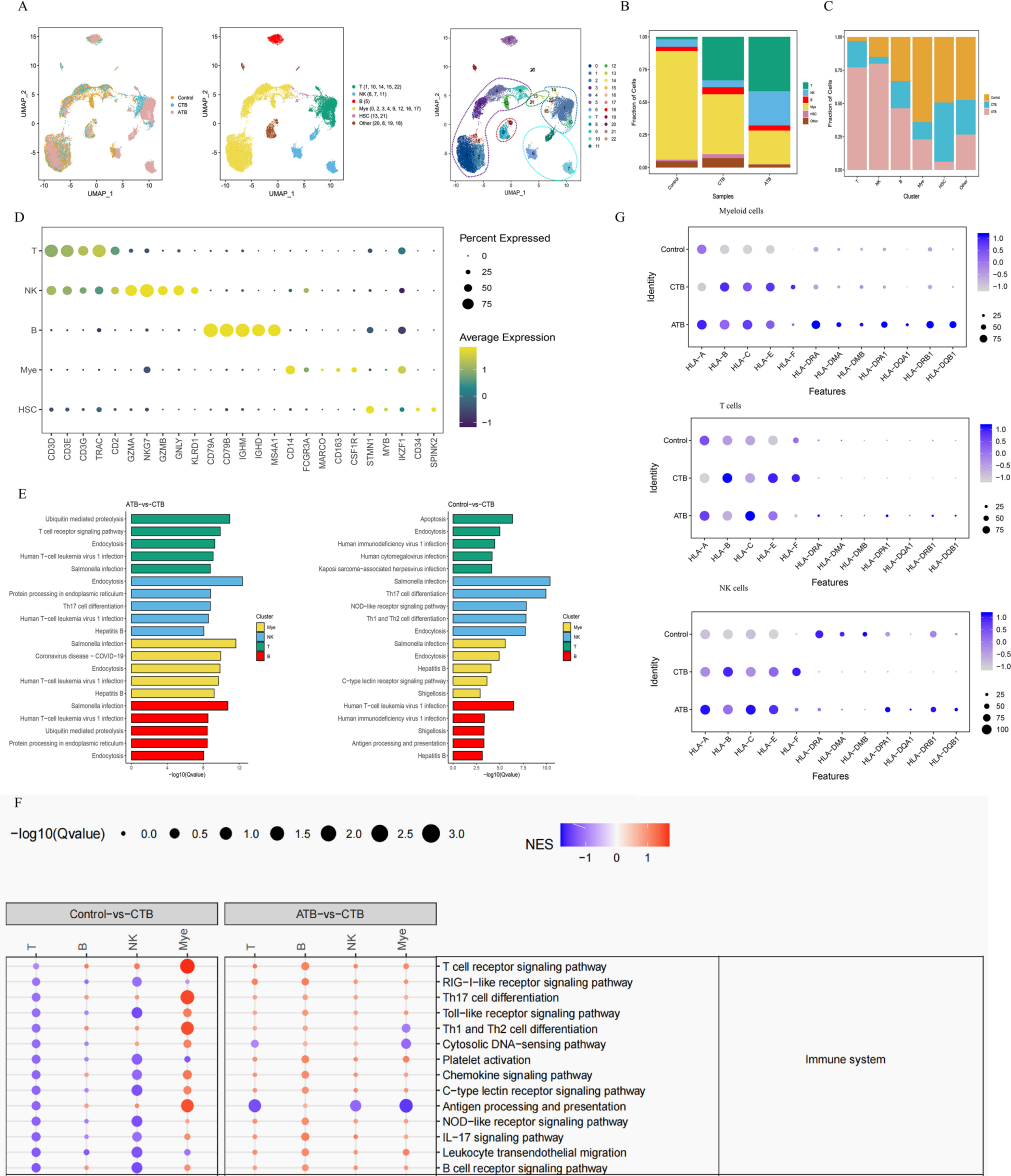
Single-cell transcriptional profiling of PBMCs from HC, CTB and ATB. **(A)** UMAP of single cell profile with each cell color-coded for sample type and associated cell type. **(B, C)** The fraction of cells for three cell type in HC, CTB and ATB. **(D)** Dot plot showing expression of immune cell markers. Dot plot color gradient represents the average gene expression scale. Dot size (pct.exp) represents the proportion of cells expressing the corresponding gene in the cell type. The larger the dot, the higher the proportion of cells expressing the gene. **(E)** KEGG enrichment analysis using the DEGs in CTB compared with HC and ATB for each cell type. The significance of DEG was analyzed by Model-based Analysis of Single-cell Transcriptomics (MAST) following the criteria including |log2FC|≥1, p_value_adjust ≤ 0.05. P values were adjusted using the Benjamini–Hochberg method for the whole gene set list. **(F)** GSEA of Control vs CTB (left) and ATB vs CTB (right) of immune system in KEGG class. Selected DEGs sets are grouped into functional/pathway categories. Dot color denotes normalized gene set enrichment score (NGS), and size indicates-log10 Q value (adjusted P value). P values were from GSEA test of the whole gene sets (Methods) and adjusted using the Benjamini–Hochberg method. The statistical significance was tested by Fisher’s exact test. **(G)** Dot plot of antigen presentation and process pathway gene sets in myeloid cells, NK cells and T cells. Dot plot size showing the scaled average mRNA expression of DEGs from the GSEA analysis of Control vs CTB and ATB vs CTB, and dot color denotes normalized gene set enrichment score (NGS).

We applied principal component analysis across all the cells and classified them into 22 groups of cell types using graph-based clustering of the informative principal components. The 22 expression groups were further grouped by hierarchical clustering, leading to the characterization of five major cellular compartments: T, B, NK, myeloid, and hematopoietic stem cells (HSCs), as well as other cell types (20-mast cells, 8,19-platelets, and 18-erythrocytes) (**Figure 2A**; **Supplementary Table 6**). Cluster-1 (24.63% of all cells) comprised T cells expressing *CD3D*, *CD3E*, *CD3G*, and *TRAC*; Cluster-2 (51.47% of all cells) comprised myeloid cells expressing *S100A8*, *S100A9*, *CD14*, and *FCN1*; Cluster-3 (3.88% of all cells) comprised B cells expressing *CD79A*, *CD79B*, *IGHM*, and *IGHd*; Cluster-4 (14.99% of all cells) comprised NK cells expressing *GZMA*, *GZMB*, *NKG7*, and *GNLY*; and Cluster-5 (1.00% of all cells) comprised HSCs expressing *STMN1*, *MYB*, *IKZF1*, and *CD34* (**Figures 2B–D**; **Supplementary Figure 1** and **Supplementary Table 7**).

The control patient had fewer T cells at this stage (four DAB); however, regarding the upward trend of the lymphocyte frequency and flow cytometry analysis results, the lack of T cells was transient. In particular, we showed the trend in lymphocyte frequency for the two patients by performing scRNA-seq, which was similar to the trend observed for the two groups, indicating the representativeness of the two samples (**Supplementary Figure 2**).

We also noticed the presence of a specific type of cell (i.e., HSCs) whose characteristics have rarely been reported. In this limited single-case comparison, HSCs appeared less abundant in the ATB patient than in the CTB and pHC infants, and relatively more abundant in the CTB infant compared with the pHC infant. These HSCs showed low expression of inflammatory markers, *S100A8* and *S100A9*, and high levels of *STMN1*, *IKZF1*, *MYB*, *SPINK2*, and *CD34* (**Figure 2D**). These marker genes play roles in the regulation of hematopoiesis, lymphoma progression, and stem cell attachment to the extracellular matrix in bone marrow. The relative enrichment of HSCs in the single CTB infant at this early stage may suggest a compensatory lymphopoietic response, which we interpret as a hypothetical early defensive mechanism against Mycobacterium tuberculosis infection.

For the differentially expressed genes (DEGs) of each cell type observed between CTB and the control or ATB, we used the Kyoto Encyclopedia of Genes and Genomes (KEGG) gene set enrichment analysis and weighed the gene effect on the gene ontology to define the pathways related to CTB. Relative to the pHC infant, DEGs in the CTB infant were enriched in apoptosis, endocytosis, Salmonella infection, and Th17 cell differentiation pathways. Compared with the ATB patient, ubiquitin-mediated proteolysis, endocytosis, and Salmonella infection pathways were more prominent in the CTB infant (**Figure 2E**; **Supplementary Table 8**).

We then used gene set enrichment analysis (GSEA) to weigh the gene effect to define the enrichment pathways involved in cellular and environmental information processes, human infectious diseases, and the immune system, and systemically assessed cell-type-specific significant enrichment pathway activity among HCs and patients with ATB and CTB based on the DEGs measured between samples. In this single-case comparison, myeloid cells in the CTB infant exhibited high expression of inflammatory factors including S100A8 and S100A9, alongside strong T cell activation signatures and elevated antigen processing and presentation signatures relative to the pHC infant. By contrast, NK and T cell effector functions appeared globally repressed in the CTB infant (**Figure 2F**; **Supplementary Figures 3A–C**, **Supplementary Table 9**). Compared with the ATB patient, antigen processing and presentation pathways in myeloid, NK, and T cells were transcriptionally less activated in the CTB infant (**Figure 2F**). These preliminary observations suggest a potential imbalance characterized by excessive innate inflammation but impaired adaptive immune activation in this CTB case. B cell subset distribution and functional signatures were comparable among the three individuals (**Supplementary Figure 4**, **Supplementary Table 10**).

The antigen processing and presentation ability plays a critical role in the ultimate outcome of TB infection (19). Therefore, we compared the gene expression signatures of the major histocompatibility complex (MHC) in the antigen presentation pathway of the three samples. We found that in myeloid cells, less expression of MHC II genes was observed in CTB compared with that in ATB, which indicated that although TB infection could induce myeloid cell activity in the early stage of infection, compared with adults, these activated cells showed decreased up regulation of co-stimulatory molecules (MHC II) that were necessary for activation of T and B lymphocytes. For NK cells, MHCI molecular expression in CTB and HC was comparable, but both exhibited lower expression of MHC II molecules than that in ATB. In T cells, MHC II expression in CTB showed reduced activation compared with that in both HC and ATB (**Figure 2G**; **Supplementary Table 11**). While these observations are limited to a single biological replicate per group, they raise the hypothesis that attenuated antigen presentation may contribute to the severe clinical course and high mortality of CTB.

### 3.4 T cell functions were universally suppressed in congenital tuberculosis

**Figure 3:**
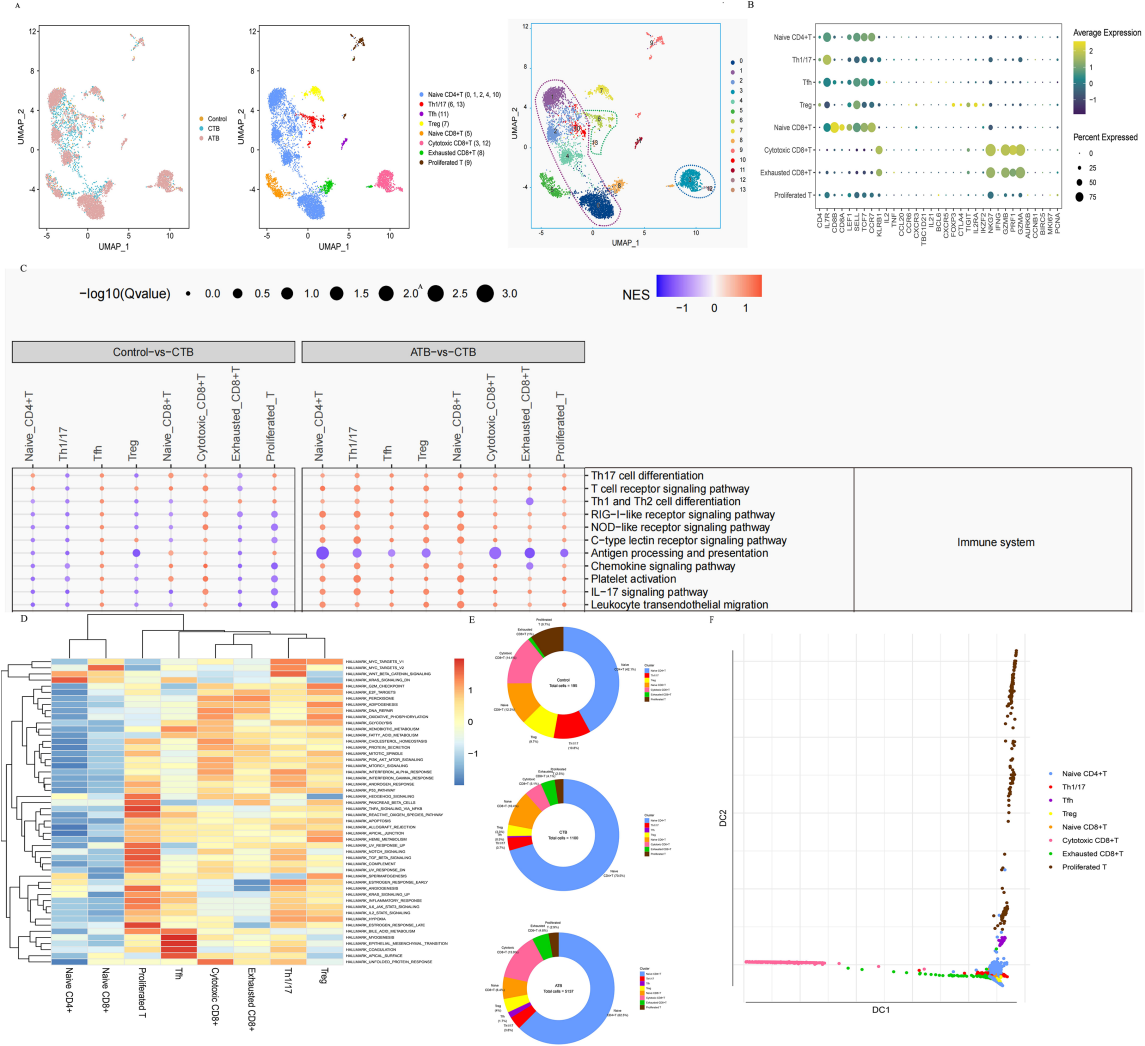
Single-cell transcriptional profiling of T cells from HC, CTB and ATB. **(A)** UMAP of single cell profile with each cell color-coded for sample type and associated cell type. **(B)** Dot plot showing expression of T cell markers. Dot plot color gradient represents the average gene expression scale. Dot size (pct.exp) represents the proportion of cells expressing the corresponding gene in the cell type. The larger the dot, the higher the proportion of cells expressing the gene. **(C)** GSEA of Control vs CTB (left) and ATB vs CTB (right) in immune system in KEGG class. Selected DEGs sets are grouped into functional/pathway categories. Dot color denotes normalized gene set enrichment score (NGS), and size indicates-log10 Q value (adjusted P value). P values were from GSEA test of the whole gene sets (Methods) and adjusted using the Benjamini–Hochberg method. The statistical significance was tested by Fisher’s exact test. **(D)** Differences in pathway activities scored per cell by GSVA between the different T cell subsets. The color denotes normalized gene set enrichment score (NGS). **(E)** The fraction of T cells subset in HC, CTB and ATB. **(F)** Diffusion map of T cell functional state transitions. DC, diffusion component.

On the basis of clinical and flow cytometry data, CTB neonates showed a declining lymphocyte trend, suggestive of insufficient anti-tuberculosis adaptive immunity. our exploratory scRNA-seq analysis detected 5,275 T cells in all three donor groups that could be subclustered into eight subsets (**Figure 3A**). The T cell subsets according to cell lineage and functional state were identified as CD4+ T (naïve CD4+ T, Th1/17, Tfh-follicular helper T, and Treg cells), CD8+ T (naïve CD8+ T, exhausted T, and cytotoxic T cells), and proliferated T cells (**Figure 3A**).

T0, T1, T2, T4, and T10 expressed high CCR7 levels, indicative of naïve-like CD4 T cells. Both T6 and T13 expressed high levels of activated CD4 T-cell markers, such as *KLRB1*, *IL17R*, *NKG7*, and *GZMA*, and were identified as Th1/17 cells (**Figure 3B**). In this single-case comparison with the pHC infant, Th1/17 cells in the CTB infant displayed transcriptional downregulation of multiple immune-related pathways (**Figure 3C**; **Supplementary Figure 3B**).

Proliferating T cells showed robust activation of TNF signaling, reactive oxygen species, IL6-JAK-STAT3, IL2-STAT5, and inflammatory response pathways, indicating strong activation (**Figure 3D**). The proportion of proliferating T cells appeared lower in the CTB and ATB samples than in the pHC sample (**Figure 3E**). Pathway analysis further suggested global downregulation of immune and infectious disease-related pathways in T cells from the CTB infant relative to the pHC infant (**Figure 3C**; **Supplementary Figure 3B**, **Supplementary Tables 12**, **13**).

Cluster T7 expressed FOXP3, CTLA4, IL2RA, and IKZF2, consistent with Tregs (**Figure 3B**). The frequency of Tregs appeared lower in the CTB and ATB samples than in the pHC sample (**Figure 3E**). Combined with longitudinal clinical data showing decreasing Treg frequency and rising CRP levels in CTB neonates, these single-sample observations support the hypothesis of disrupted immune homeostasis and excessive inflammation during CTB progression.

We identified three diverse CD8 T cell subsets: naïve CD8 T (T5: CD8A, CD8B, and CCR7), cytotoxic CD8 T (T3 and T12: GZMA, GZMB, NKG7, KLRB1, and PRF1), and exhausted CD8 T cells (T8: weaker expression of GZMA, GZMB, NKG7, KLRB1, and PRF1) (**Figures 3A, B**). The cytotoxic CD8+ T cell subsets displayed up-regulated pathways associated with peroxisomes, oxidative phosphorylation, fatty acid metabolism, and interferon alpha and interferon gamma (IFN-γ) responses, indicating that these cells had a high energy metabolism and were highly active in infectious diseases and immune systems (**Figure 3D**). In this limited comparison, the proportion of cytotoxic CD8+ T cells was lower in the CTB infant than in the pHC and ATB individual (**Figure 3E**), which may suggest a hypothetical defect in controlling mycobacterial replication. Exhausted CD8+ T cells represented a group of dysfunctional T cells, which are present in chronic infections or tumors (20, 21). We identified more exhausted T cells in patients with both CTB and ATB, which may indicate a dysfunctional state of T cells during TB infection (**Figure 3E**).

To understand the state transitions among T cell subtypes, we applied destiny in R software to draw diffusion maps to construct potential developmental trajectories of T cell subtypes. The developmental trajectory suggested that naïve CD8+ T cells may eventually enter a cytotoxic CD8+ T cell state (**Figure 3F**). In CD4+ T cells, naïve T cells may eventually enter a proliferated T cell state through Tfh cells (**Figure 3F**). These diffusion map analysis identified model-based trajectory patterns of T cell subset differentiation, which should be interpreted as computational inferences rather than definitive lineage evidence.

### 3.5 Myeloid cell functions were activated in congenital tuberculosis but showed reduced antigen presentation function than that in adult tuberculosis

**Figure 4:**
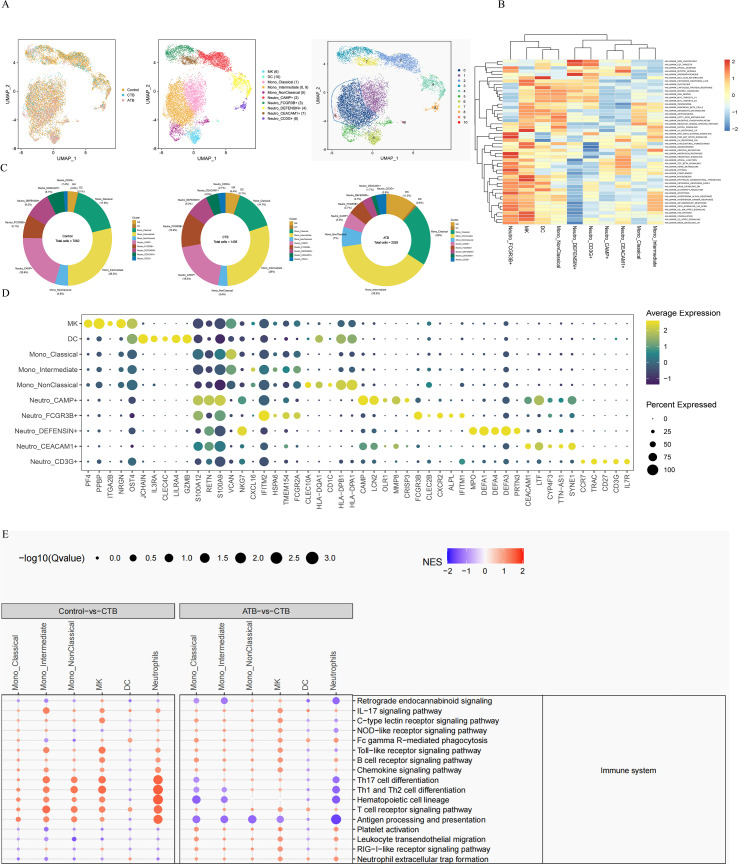
Single-cell transcriptional profiling of myeloid cells from HC, CTB and ATB. **(A)** UMAP of single cell profile with each cell color-coded for sample type and associated cell type. **(B)** Differences in pathway activities scored per cell by GSVA between the different T cell subsets. The color denotes normalized gene set enrichment score (NGS). **(C)** The fraction of myeloid cells subset in HC, CTB and ATB. **(D)** Dot plot showing expression of myeloid cell markers. Dot plot color gradient represents the average gene expression scale. Dot size (pct.exp) represents the proportion of cells expressing the corresponding gene in the cell type. The larger the dot, the higher the proportion of cells expressing the gene. **(E)** GSEA of Control vs CTB (left) and ATB vs CTB (right) in immune system in KEGG class. Selected DEGs sets are grouped into functional/pathway categories. Dot color denotes normalized gene set enrichment score (NGS), and size indicates-log10 Q value (adjusted P value). P values were from GSEA test of the whole gene sets (Methods) and adjusted using the Benjamini–Hochberg method. The statistical significance was tested by Fisher’s exact test.

Our exploratory scRNA-seq analysis identified 11,026 myeloid cells in all three donor groups that could be subclustered into 10 subsets (**Figure 4A**; **Supplementary Table 14**). The myeloid cell subsets according to cell lineage and marker genes, as well as gene set variation analysis (GSVA) hallmark pathway characteristics, were identified as three subsets of monocytes [classical (CD14+CD16), nonclassical (CD14lowCD16+), and intermediate (CD14+CD16+) monocytes], dendritic cells (high expression levels of *CLEC4C*, *IL3RA*, and *GZMB*), five subsets of neutrophils (CAMP+, FCGR3B+, DEFENSIN+, CEACAM1+, and CD3G+), and megakaryocytes (high expression levels of *PF4* and *PPBP*) (**Figures 4B–D**).

The overall distribution of myeloid cell subsets appeared similar between the single CTB and pHC infants (**Figure 4C**). Next, we systemically assessed the cell-type-specific significant enrichment pathway activity and found that compared with HCs, myeloid cells in CTB were generally more activated in the immune response, which potentially indicated a stronger T cell activation signature, increased antigen processing and presentation ability. Compared with ATB, the frequencies of the five subsets of neutrophils were higher. These neutrophils showed high expression of antimicrobial genes, such as *CAMP*, *DEFFESIN*, *FCGR3B*, *MPO*, *S100A9*, and *S100A12*. However, compared with ATB, the pathway of antigen processing and presentation was much weaker (**Figure 4E**; **Supplementary Figures 3C**, **Supplementary Table 13**). Antigen processing and presentation is critical for T cell activity (3, 19, 22, 23). These single-case observations raise the hypothesis that CTB is characterized by robust innate activation but impaired ability to prime TB-specific adaptive immunity.

### 3.6 Cytotoxic natural killer cells were reduced in congenital tuberculosis

**Figure 5:**
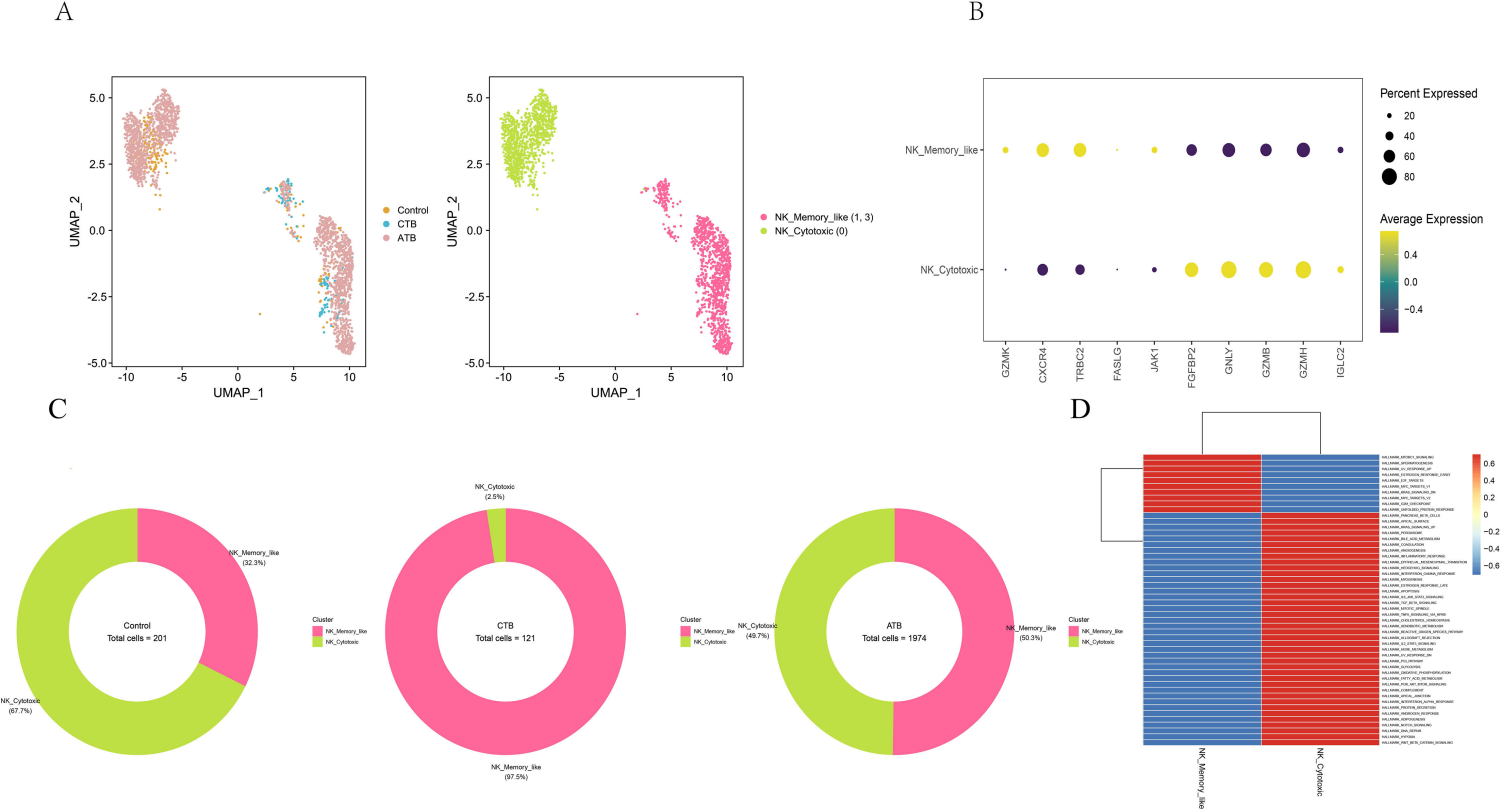
Single-cell transcriptional profiling of NK cells from HC, CTB and ATB. **(A)** UMAP of single cell profile with each cell color-coded for sample type and associated cell type. **(B)** Dot plot showing expression of myeloid cell markers. Dot plot color gradient represents the average gene expression scale. Dot size (pct.exp) represents the proportion of cells expressing the corresponding gene in the cell type. The larger the dot, the higher the proportion of cells expressing the gene. **(C)** The fraction of myeloid cells subset in HC, CTB and ATB. **(D)** Differences in pathway activities scored per cell by GSVA between the different NK cell subsets. The color denotes normalized gene set enrichment score (NGS).

The scRNA-seq analysis detected 3,211 NK cells in all three donor groups that could be subclustered into three subsets (**Figure 5A**, **Supplementary Table 10**). The NK cell subsets according to marker genes and GSVA hallmark pathway characteristics were identified as memory-like NK (high expression of *CXCR4* and *TRBC2*) and cytotoxic NK cells (high expression levels of *GZMB* and *GNLY*) (**Figures 5A–C**).

Cytotoxic NK cells displayed strong IFN−γ response and immune effector pathway activation (**Figure 5D**). NK cells play a significant role in the host defense to a variety of infections, with the capacity to secrete IFN-γ and perform cytolytic functions (21). In this exploratory single-replicate comparison, the frequency of cytotoxic NK cells was lower in the CTB infant than in the pHC and ATB individual (**Figure 5C**). Cell-type-specific pathway analysis further suggested reduced immune and anti-infectious activity in NK cells from the CTB infant compared with the pHC infant (**Figure 2F**). The functionally significant reductions in IFN-γ and TNF-α production activity, as well as the reduced cytotoxic function of NK cells in preterm infants may be one of the immune factors contributing to the gradual deterioration of symptoms under CTB infection.

## 4 Discussion

In the present study, we characterized the dynamic clinical and immunological features of congenital tuberculosis (CTB) using longitudinal peripheral blood flow cytometry and exploratory single-cell RNA sequencing (scRNA-seq). Compared with paired healthy control (pHC), CTB neonates exhibited declining lymphocyte and platelet counts over disease progression, accompanied by elevated neutrophil proportions and persistently increased C-reactive protein (CRP) levels. We also observed reduced frequencies of regulatory T cells (Tregs) in CTB patients. Collectively, these findings suggest that gradual lymphocyte depletion, diminished Treg proportions, and excessive innate immune activation may contribute to an overwhelming pro-inflammatory state alongside impaired pathogen-specific adaptive immunity in CTB.

To provide mechanistic insights into these immune dynamics, we performed exploratory, hypothesis-generating scRNA-seq in one CTB neonate, one pHC neonate, and one adult tuberculosis (ATB) patient. This analysis represents only a single biological replicate per group, and thus all transcriptomic observations are inherently limited in generalizability. As highlighted in recent literature, host genetic background-including common single nucleotide polymorphisms (SNPs) in HLA and innate immune pathways, as well as rare inborn errors of immunity-profoundly shapes inter-individual variability in immune responses to mycobacterial infection, further restricting the generalizability of observations derived from a single case (24). With these constraints in mind, our primary and most robust mechanistic interpretations are centered exclusively on the within-neonate comparison between the CTB neonate and the age-matched pHC neonate, which avoids confounding from developmental immune ontogeny. In this internally controlled comparison, myeloid cells in the CTB infant showed globally activated inflammatory signatures and high expression of pro-inflammatory mediators, whereas T and NK cell functions appeared transcriptionally suppressed relative to the pHC infant.

When comparing the CTB neonate with the ATB adult, innate and adaptive immune cells in the CTB neonate uniformly displayed weaker antigen-presentation signatures. However, such cross-age comparisons are inherently confounded by fundamental differences in immune ontogeny-preterm neonates intrinsically express lower baseline levels of MHC-II and exhibit diminished antigen-presentation and Th1 polarization capacity relative to fully mature adults, independent of infectious or disease status (3, 19, 22–24). Therefore, transcriptomic differences between the CTB neonate and the ATB adult cannot be attributed solely to disease-specific immune alterations and must be interpreted as exploratory and illustrative rather than definitive. These preliminary immune profiles may help explain the unique neonatal immune features that underlie the high mortality associated with CTB. To our knowledge, this is the first study to integrate longitudinal clinical monitoring, flow cytometry, and scRNA-seq to characterize immune cell dynamics in CTB. However, given the epistemological limitations of the scRNA-seq sample size and the complexity of the neonatal intensive care unit (NICU) environment, all transcriptomic interpretations are exploratory and require cautious interpretation. Further studies with expanded scRNA-seq cohorts are urgently needed to validate these preliminary observations.

Consistent with previous reports of 170 CTB infants, our cohort exhibited leukocytosis with neutrophil predominance, elevated CRP, and thrombocytopenia (3). A previous study predicting pulmonary TB in South African adults showed that a significantly higher CRP level could achieve high sensitivity in the diagnosis of TB (18). Another study on TB showed a decreased frequency of lymphocytes in active ATB (25). Unlike prior cross-sectional studies, we identified progressive, longitudinal declines in lymphocytes and platelets and reciprocal increases in neutrophils and CRP, which persisted despite the administration of broad-spectrum antibiotics. We also provided a concise table of neonatal baseline characteristics for both CTB and pHC groups. The two groups were comparable, except that infants in CTB group received longer antibiotic therapy - the pHC cohort averaged 3.67 days, while the CTB cohort averaged 15.0 days. This difference is clinically inevitable, as the prolonged antibiotic therapy in the CTB group directly reflects the empirical management of severe, undifferentiated infection in the NICU setting before the confirmation of tuberculosis. The prolonged broad-spectrum antibiotic exposure represents a significant clinical confounder. Antibiotic-mediated alterations to the microbiome and subsequent modulation of the baseline inflammatory tone could potentially contribute to the hyper-activated myeloid cell signatures observed in our scRNA-seq data. However, the progressive and sustained nature of the inflammatory response (such as rising CRP, falling lymphocytes) in the CTB cohort, even after two weeks of antibiotic therapy, suggests that these changes are unlikely to be solely attributable to antibiotic exposure or non-specific NICU inflammation. Instead, this pattern supports the interpretation that the observed longitudinal immunological alterations are primarily driven by the underlying CTB pathophysiology, with antibiotic exposure potentially representing a secondary modifying factor. Close monitoring of these routine laboratory parameters may therefore assist in the early recognition and monitoring of suspected CTB, although their combined predictive value, independent of antibiotic effects, requires formal investigation in larger cohorts.

Innate immune cells are the first to encounter M.tb, and their responses dictate the course of infection (26, 27). Recent studies on human neutrophils have suggested that they become activated and secrete numerous cytokines in response to TB infection (26). In the present study, we classified five distinct neutrophils that were more activated compared with those in HCs; these neutrophils showed a higher frequency than that in ATB, but a weaker antigen processing ability. Neutrophils are normally associated with the control of bacterial infections and are emerging as key drivers of the hyperinflammatory response (26). The recruitment of these activated innate immune cells in CTB may serve as an early line of defense against TB infection, but also induces excessive inflammation, together with insufficient antigen processing ability, which may result in progressive disease. Monocytes are the main antigen-presenting cells linking the innate and adaptive immune systems (28). We found that although monocytes showed a higher activated antigen presentation and process pathway compared with that in HCs, only higher expression of MHC I genes were observed with no MHC II genes activated, which may indicate an impaired stimulating ability of the adaptive immune system in CTB.

Although monocyte phenotypes are largely mature at birth (29), GSEA revealed comparable myeloid immune activity between the CTB neonate and the ATB adult, but significantly weaker MHC class II expression in the CTB sample. MHC class II is essential for effective antigen presentation to T and B lymphocytes (30), and its blunted upregulation may impair anti-mycobacterial immunity and contribute to severe disease (19, 22). However, cross-age comparisons are inherently confounded by neonatal immune immaturity, and differences in clinical severity, study design, technical platforms, and biological confounders may also contribute to the observed inter-group differences in MHC expression. Cross-age single-cell comparisons therefore remain exploratory and hypothesis-generating and require validation in larger, harmonized studies.

T cells play a critical role in controlling M.tb infection (22, 23). A study on fetal immune development showed that T cell phenotypes in newborns exhibited no sign of convergence with adult-like phenotypes (29). The immune system of preterm infants exhibits distinct functions compared with those of more mature and older humans (11, 29). However, how adaptive immune cells act in response to M.tb infection during the early stages in neonates has not been reported previously. A decrease in lymphocytes has been reported in active ATB (25). A previous study on adults showed that progressive depletion and dysfunction of CD4+ T cells following HIV infection leads to immune suppression and has a negative impact on immunity against M.tb (31). Similar to patients with HIV, we observed a gradual depletion of lymphocytes, especially an obvious depletion in Tregs in the lymphocyte subsets of infants with CTB. Further, T cell function was generally suppressed compared with that in HCs, which was not observed in ATB. Moreover, the antigen presentation ability of T cells was weaker than that in ATB. However, the latter observation must be interpreted with caution due to developmental differences in immune ontogeny between neonates and adults, which may independently attenuate antigen-presentation function. These findings suggest impaired development of tuberculosis-specific adaptive immune surveillance in CTB.

Cytotoxic CD8+ T cells limit M.tb replication by secreting cytolytic effector molecules (19, 22, 23). In contrast, exhausted CD8+ T cells are characterized by attenuated effector cytotoxicity (20). In our scRNA-seq data, the CTB infant showed reduced proportions of cytotoxic CD8+ T cells and increased exhausted CD8+ T cells, suggesting CD8+ T cell dysfunction. These preliminary findings are exploratory due to the n=1 design but generate the testable hypothesis that impaired cytotoxic T cell activity contributes to CTB pathogenesis.

Th17 cells produce the pro-inflammatory cytokine, IL-17, and contribute to the response against M.tb infection (19, 26). In adults, a significantly high frequency of IL-17-producing CD4+ T cells was identified in the PBMCs and bronchoalveolar lavage fluid of BCG-vaccinated healthy individuals, which declined in adults with active TB disease (23). In our study, Th17 cell proportions were decreased in both ATB and CTB. Moreover, Th17 cells in the CTB infant showed weaker antigen-presentation and processing signatures than in the ATB patient; however, this difference may reflect both disease-related changes and inherent developmental immaturity of the neonatal immune system.

FoxP3+ Tregs are immune suppressor cells (19, 32). A previous study showed that depletion of Tregs before and early after ATB infection enhances the control of bacterial burden (33). The frequency of Tregs in patients with CTB and active ATB was lower than that in HCs. The limited expansion of Treg subsets during the early stages may contribute to an optimal response against M.tb (19). However, the progressive loss of Tregs in chronically infected TB could impair T cell function (23, 33). Active TB is characterized by inflammation and the up-regulation of acute-phase proteins, which in turn can affect T cell function and development (24, 34). In the current study, we observed a reduced proportion of Tregs and sustained increase in CRP levels during CTB deterioration. This may indicate that Treg depletion is potentially associated with increased inflammation, which might result in tissue impairment and adverse outcomes in the later stages of TB infection in neonates.

NK cells are innate lymphocytes with the capacity to perform cytolytic functions to mediate the control of M.tb (19). A previous study on TB in adults showed that depletion of cytotoxic NK cell subsets could help identify patients with active ATB (25). In our study, the frequency of cytotoxic NK cells in CTB was lower than that in both pHC and ATB, and the cytolytic capacity of NK cells was diminished in patients with CTB relative to HCs. A fetal study demonstrated the development of adult-like phenotypes of NK cells in neonates (29). This indicated that a lower frequency of activated cytotoxic NK cells in the early infection stage might represent candidate immune patterns that hold potential for distinguishing patients with CTB.

A previous study on adults showed that platelets participate in the immune response against M.tb through the release of antimicrobial molecules, such as factor platelet 4 and inflammatory cytokines (35, 36). However, no information is available on the characteristics of platelets during CTB infection. We initially found that during the early stages of TB infection, the PLT was comparable with that in HCs, and MK cells showed a higher immune ability than that in HCs; however, the PLT dramatically decreased as the disease deteriorated. Considering the contribution of platelets to anti-TB immunity, progressive platelet exhaustion during TB infection may further impair anti-mycobacterial defenses in neonates.

### 4.1 Limitations

This study has several important limitations, particularly regarding the epistemological constraints of scRNA-seq sample size. First, scRNA-seq was performed on only one individual per group (one CTB neonate, one pHC neonate, one ATB patient). With single biological replicates, it is impossible to determine whether the observed transcriptomic shifts reflect universal pathophysiological features of CTB or individual-specific genetic and developmental idiosyncrasies. As highlighted by Conti et al., host genetic background, including common polymorphisms in HLA and innate immune signaling pathways and rare inborn errors of immunity, profoundly shapes susceptibility and immune responses to mycobacterial infection (24). Such inter-individual genetic variability severely limits the generalizability of n=1 transcriptomic data. Accordingly, all scRNA-seq findings in this study are explicitly interpreted as exploratory and hypothesis-generating, not definitive population-level conclusions. To mitigate this limitation, we leveraged flow cytometry data from our larger 9−patient CTB cohort to orthogonally validate key scRNA-seq observations, including Treg depletion and trends in lymphocyte subset dynamics. However, additional protein-level validation of other critical findings-such as reduced cytotoxic CD8+ T cells and altered NK cell frequencies-could not be performed due to the lack of remaining clinical samples, which is acknowledged as a major limitation. Second, critical consideration must be given to neonatal immune ontogeny when interpreting immune differences between congenital tuberculosis and adult tuberculosis. Preterm infants exhibit profound developmental immaturity in antigen−presenting cell function, including intrinsically lower baseline expression of MHC−II molecules, diminished co−stimulatory capacity, and reduced Th1−polarizing potential relative to fully mature adults (28, 29). These developmental differences are independent of infectious or disease status and represent inherent features of the neonatal immune system. Accordingly, direct cross−age comparison between the CTB neonate and the ATB adult is inherently confounded by developmental immune immaturity, and observed differences in MHC−II expression, antigen processing, and T cell function cannot be solely attributed to disease−specific immune evasion mechanisms. To isolate true CTB−specific immune signatures, all mechanistic conclusions in this study should be anchored on the internal, age−matched comparison between the CTB neonate and the pHC neonate. Transcriptomic differences between the CTB neonate and the ATB adult should therefore be interpreted as exploratory and illustrative. This rigorous focus on the internally controlled contrast strengthens the interpretability of our findings and ensures that conclusions regarding CTB pathogenesis are not conflated with inherent developmental immaturity of the neonatal immune system. Third, most patients deteriorated rapidly despite early intervention, precluding analysis of immune dynamics following anti-tuberculosis therapy.

## 5 Conclusion

This study delineates dynamic immune and molecular signatures in CTB using complementary clinical, flow cytometric, and exploratory scRNA-seq approaches. These findings advance our understanding of CTB pathophysiology and identify candidate immune markers with potential utility for early diagnosis, thus potentially shifting the paradigm of CTB management from rescue therapy to early intervention. However, given the limited scRNA-seq sample size, host genetic variability, and lack of comprehensive protein validation, all transcriptomic conclusions remain preliminary and require rigorous validation in larger, prospective cohorts.

## Data Availability

The datasets presented in this study can be found in online repositories. The names of the repository/repositories and accession number(s) can be found in the article/**Supplementary Material**.

## Supplementary material

Supplementary Figure 1 Expression of marker genes for the main cell types. Color gradient (from light to dark, 0→3): The larger the value and the darker the color, the higher the expression level of the gene in the corresponding cells.

Supplementary Figure 2 The dynamic features of lymphocytes frequency in two patients conducting single-cell seq. **(A)** control patient. **(B)** CTB patient.

Supplementary Figure 3 GSEA of Control vs CTB and ATB vs CTB in main cell types of PBMC **(A)**, myeloid cells **(B)** and T cells subsets **(C)**. Selected DEGs sets are grouped into functional/pathway categories. Dot color denotes normalized gene set enrichment score, and size indicates-log10 (adjusted P value). P values were from GSEA test of the whole gene sets (Methods) and adjusted using the Benjamini–Hochberg method.

Supplementary Figure 4 Single-cell transcriptional profiling of B cells from HC, CTB and ATB. **(A)** UMAP of single cell profile with each cell color-coded for sample type and associated cell type. **(B)** Dot plot showing expression of B cell markers. Dot plot color gradient represents the average gene expression scale. Dot size (pct.exp) represents the proportion of cells expressing the corresponding gene in the cell type. The larger the dot, the higher the proportion of cells expressing the gene. **(C)** The fraction of B cells subset in HC, CTB and ATB. **(D)** Differences in pathway activities scored per cell by GSVA between the different B cell subsets. The color denotes normalized gene set enrichment score (NGS).

Supplementary Table 5 The raw data of routine blood tests and the frequencies of lymphocyte subset from HC and CTB.

Supplementary Table 6 The cell numbers and frequency of all subsets in PBMC from HC, CTB and ATB, and Characteristics of scRNA-seq of the PBMC samples included in this study.

Supplementary Table 7 Significantly different expressed genes of PBMC major cell types identified by scRNA-seq.

Supplementary Table 8 KEGG pathway enrichment analysis of DEGs in three samples.

Supplementary Table 9 GSEA of Control vs CTB and ATB vs CTB in main cell types of PBMC.

Supplementary Table 10 Significantly different expressed genes of B cell and NK cell subsets identified by scRNA-seq.

Supplementary Table 11 The gene expression of MHCI and MHCII in myeloid cells, T cells and NK cells.

Supplementary Table 12 Significantly different expressed genes of T cell subsets identified by scRNA-seq.

Supplementary Table 13 GSEA of Control vs CTB and ATB vs CTB in T and myeloid cells subsets.

Supplementary Table 14 Significantly different expressed genes of myeloid subsets identified by scRNA-seq.

Supplementary File 1 1.1 The representative gating strategy figure of Lymphocyte immune cells flow-cytometric analysis; 1.2 UMAP plots before and after integration; 1.3 The custom R processing scripts.

## References

[1] Trajman A Campbell JR Kunor T Ruslami R Amanullah F Behr MA . Tuberculosis. Lancet. (2025) 405:850–66. doi: 10.1007/978-3-031-15955-8_27. PMID: 40057344

[2] Floyd K Glaziou P Zumla A Raviglione M . The global tuberculosis epidemic and progress in care, prevention, and research: an overview in year 3 of the End TB era. Lancet Respir Med. (2018) 6:299–314. doi: 10.1016/s2213-2600(18)30057-2. PMID: 29595511

[3] Shao Y Hageman JR Shulman ST . Congenital and perinatal tuberculosis. Neoreviews. (2021) 22:e600–5. doi: 10.1542/neo.22-9-e600. PMID: 34470761

[4] WHO . Global tuberculosis report 2024 (2024). Available online at: https://www.who.int/teams/global-programme-on-tuberculosis-and-lung-health/data (Accessed December 30, 2024).

[5] De Schutter I Schepers K Singh M Mascart F Malfroot A . Latent tuberculosis in a newborn: diagnostic challenges. Eur J Pediatr. (2010) 169:1155–8. doi: 10.1007/s00431-010-1177-8. PMID: 20411276

[6] Newberry DM Robertson Bell T . Congenital tuberculosis: a new concern in the neonatal intensive care unit. Adv Neonatal Care. (2018) 18:341–9. 10.1097/ANC.0000000000000555 30096058

[7] Orazulike N Sharma JB Sharma S Umeora OUJ . Tuberculosis (TB) in pregnancy - a review. Eur J Obstet Gynecol Reprod Biol. (2021) 259:167–77. doi: 10.1016/j.ejogrb.2021.02.016. PMID: 33684671

[8] Saramba MI Zhao D . A perspective of the diagnosis and management of congenital tuberculosis. J Pathog. (2016) 2016:8623825. doi: 10.1155/2016/8623825. PMID: 27999684 PMC5143719

[9] Zhang X Zhuxiao R Xu F Zhang Q Yang H Chen L . Congenital tuberculosis after *in vitro* fertilization: suggestion for tuberculosis tests in infertile women in developing countries. J Int Med Res. (2018) 46:5316–21. doi: 10.1177/0300060518808179. PMID: 30453806 PMC6300949

[10] Liu CH Liu H Ge B . Innate immunity in tuberculosis: host defense vs pathogen evasion. Cell Mol Immunol. (2017) 14:963–75. doi: 10.1038/cmi.2017.88. PMID: 28890547 PMC5719146

[11] Gustafsson A Bernhardsson AK Zhang C Bohlin K Brodin P . Stereotypic immune system development in newborn children. Cell. (2018) 174:1277–1292.e14. doi: 10.1016/j.cell.2018.06.045. PMID: 30142345 PMC6108833

[12] Collins A Weitkamp JH Wynn JL . Why are preterm newborns at increased risk of infection? Arch Dis Child Fetal Neonatal Ed. (2018) 103:F391–4. doi: 10.1136/archdischild-2017-313595. PMID: 29382648 PMC6013388

[13] Peterson LS Hedou J Ganio EA Stelzer IA Feyaerts D Harbert E . Single-cell analysis of the neonatal immune system across the gestational age continuum. Front Immunol. (2021) 12:714090. doi: 10.3389/fimmu.2021.714090. PMID: 34497610 PMC8420969

[14] Chang CW Wu PW Yeh CH Wong KS Wang CJ Chang CC . Congenital tuberculosis: case report and review of the literature. Paediatr Int Child Health. (2018) 38:216–9. doi: 10.1080/20469047.2017.1315912. PMID: 28421876

[15] P R Yk S . Congenital tuberculosis in a neonate: a diagnostic dilemma. J Neonatal Surg. (2014) 3:49. 26023520 PMC4420334

[16] Tuberculosis: an ongoing global epidemic. EClinicalMedicine. (2021) 33:100785. doi: 10.1016/j.eclinm.2021.100785. PMID: 33842869 PMC8020150

[17] Cantwell MF Shehab ZM Costello AM Sands L Green WF Ewing EP . Brief report: congenital tuberculosis. N Engl J Med. (1994) 330:1051–4. doi: 10.1056/nejm199404143301505. PMID: 8127333

[18] Calderwood CJ Reeve BW Mann T Palmer Z Nyawo G Mishra H . Clinical utility of C-reactive protein-based triage for presumptive pulmonary tuberculosis in South African adults. J Infect. (2023) 86:24–32. doi: 10.1016/j.jinf.2022.10.041. PMID: 36375640 PMC10567578

[19] Jasenosky LD Scriba TJ Hanekom WA Goldfeld AE . T cells and adaptive immunity to Mycobacterium tuberculosis in humans. Immunol Rev. (2015) 264:74–87. doi: 10.1111/imr.12274. PMID: 25703553

[20] Huang ZY Shao MM Zhang JC Yi FS Du J Zhou Q . Single-cell analysis of diverse immune phenotypes in Malignant pleural effusion. Nat Commun. (2021) 12:6690. doi: 10.1038/s41467-021-27026-9. PMID: 34795282 PMC8602344

[21] Miller BC Sen DR Al Abosy R Bi K Virkud YV LaFleur MW . Subsets of exhausted CD8+ T cells differentially mediate tumor control and respond to checkpoint blockade. Nat Immunol. (2019) 20:326–36. doi: 10.1038/s41590-019-0312-6. PMID: 30778252 PMC6673650

[22] Shao MM Yi FS Huang ZY Peng P Wu FY Shi HZ . T cell receptor repertoire analysis reveals signatures of T cell responses to human Mycobacterium tuberculosis. Front Microbiol. (2022) 13:829694. doi: 10.3389/fmicb.2022.829694. PMID: 35197957 PMC8859175

[23] Larsen SE Williams BD Rais M Coler RN Baldwin SL . It takes a village: the multifaceted immune response to Mycobacterium tuberculosis infection and vaccine-induced immunity. Front Immunol. (2022) 13:840225. doi: 10.3389/fimmu.2022.840225. PMID: 35359957 PMC8960931

[24] Conti F Moratti M Cinicola BL Castagnoli R Papa R Federici S . Unraveling the genetic predisposition on respiratory infections: from single nucleotide polymorphisms to inborn errors of immunity. Interdiscip Perspect Infect Dis. (2025). doi: 10.1155/ipid/1081820. PMID: 41018718

[25] Cai Y Dai Y Wang Y Yang Q Guo J Wei C . Single-cell transcriptomics of blood reveals a natural killer cell subset depletion in tuberculosis. EBioMedicine. (2020) 53:102686. doi: 10.1016/j.ebiom.2020.102686. PMID: 32114394 PMC7047188

[26] Xu W Snell LM Guo M Boukhaled G Macleod BL Li M . Early innate and adaptive immune perturbations determine long-term severity of chronic virus and Mycobacterium tuberculosis coinfection. Immunity. (2021) 54:526–541.e7. doi: 10.1016/j.immuni.2021.01.003. PMID: 33515487 PMC7946746

[27] Scott NR Swanson RV Al-Hammadi N Domingo-Gonzalez R Rangel-Moreno J Kriel BA . S100A8/A9 regulates CD11b expression and neutrophil recruitment during chronic tuberculosis. J Clin Invest. (2020) 130:3098–112. doi: 10.1172/jci130546. PMID: 32134742 PMC7259997

[28] Sampath P Moideen K Ranganathan UD Bethunaickan R . Monocyte subsets: phenotypes and function in tuberculosis infection. Front Immunol. (2018) 9:1726. doi: 10.3389/fimmu.2018.01726. PMID: 30105020 PMC6077267

[29] Jardine L Webb S Goh I Quiroga Londoño M Reynolds G Mather M . Blood and immune development in human fetal bone marrow and Down syndrome. Nature. (2021) 598:327–31. doi: 10.1038/s41586-021-03929-x. PMID: 34588693 PMC7612688

[30] Pishesha N Harmand TJ Ploegh HL . A guide to antigen processing and presentation. Nat Rev Immunol. (2022) 22:751–64. doi: 10.1038/s41577-022-00707-2. PMID: 35418563

[31] Esmail H Riou C Bruyn D Lai RP Harley YXR Meintjes G . The immune response to Mycobacterium tuberculosis in HIV-1-coinfected persons. Annu Rev Immunol. (2018) 36:603–38. doi: 10.1146/annurev-immunol-042617-053420. PMID: 29490165

[32] Rubtsov YP Rasmussen JP Chi EY Fontenot J Castelli L Ye X . Regulatory T cell-derived interleukin-10 limits inflammation at environmental interfaces. Immunity. (2008) 28:546–58. doi: 10.1016/j.immuni.2008.02.017. PMID: 18387831

[33] Wu YE Zhang SW Peng WG Li KS Li K Jiang JK . Changes in lymphocyte subsets in the peripheral blood of patients with active pulmonary tuberculosis. J Int Med Res. (2009) 37:1742–9. doi: 10.1177/147323000903700610. PMID: 20146872

[34] Mulenga H Fiore-Gartland A Mendelsohn SC Penn-Nicholson A Mbandi SK Nemes E . Evaluation of a transcriptomic signature of tuberculosis risk in combination with an interferon gamma release assay: a diagnostic test accuracy study. EClinicalMedicine. (2022) 47:101396. doi: 10.1016/j.eclinm.2022.101396. PMID: 35497063 PMC9046130

[35] Feng Y Dorhoi A Mollenkopf HJ Yin H Dong Z Mao L . Platelets direct monocyte differentiation into epithelioid-like multinucleated giant foam cells with suppressive capacity upon mycobacterial stimulation. J Infect Dis. (2014) 210:1700–10. doi: 10.1093/infdis/jiu355. PMID: 24987031 PMC4224136

[36] Fox KA Kirwan DE Whittington AM Krishnan N Robertson BD Gilman RH . Platelets regulate pulmonary inflammation and tissue destruction in tuberculosis. Am J Respir Crit Care Med. (2018) 198:245–55. doi: 10.1164/rccm.201710-2102oc. PMID: 29420060 PMC6058979

