# Supplementary material for: Immunopathological signatures in congenital tuberculosis-a case-matched study
Source: Front Immunol. 2026 Mar 30;17:1614510. doi: 10.3389/fimmu.2026.1614510 (PMC13070812; doi:10.3389/fimmu.2026.1614510)
Supplement: Supplementary file 2 [file Table1.docx]

Supplemental table 1. Demographic characteristics of the CTB populations and the mothers.

| Patient No. | Gender  (male=0,  female=1) | GA  (weeks) | BW  (g) | Delivery  (CS=1, natural delivery=0) | IVF | Deterioration   (days after   birth) | Diagnosis (days after birth) | TB  associated  suspected  symptoms | Diagnosis method | | | | Chest  Radiography- miliary  tuberculosis | Treatment starting time (DAB) | Death  (DAB) | Maternal  symptoms | MTB  diagnosis |
| --- | --- | --- | --- | --- | --- | --- | --- | --- | --- | --- | --- | --- | --- | --- | --- | --- | --- |
|  |  |  |  |  |  |  |  |  | PCR | Sputum   smear  acid fast  staining | IGRA | NGS |  |  |  |  |  |
| 1DHL | 1 | 27.00 | 1060 | 1 | 1, salpingemphraxis | 14 | 22 | numerous  miliary nodules in chest  radiography | - | √ | - | √,  blood | √ | 22 | 23 | salpingemphraxis | after delivery, tuberculosis  antibody |
| 2YJZ | 1 | 34.00 | 2400 | 0 | 0 | 22 | 28 | septic shock ,  resistant to   antibiotic therapy | √,  blood |  |  |  | - | 30 | 30 | no specific  symptoms | after delivery, miliary  tuberculosis |
| 3CDN | 1 | 31.57 | 1540 | 0 | 0 | 42 | 54 | Persistent fever,  miliary nodules  in CT, resistant to antibiotic  therapy |  | √ | √ |  | √ | 54 | 55 | recurrent fever before  delivery | after delivery, miliary  tuberculosis |
| 4ZJJ | 1 | 33.43 | 1220 | 1 | 0 | 24 | 30 | Deterioration   after intubation, resistant to  Antibiotic   therapy |  | √ |  |  | - | 31 | - | transient fever after  delivery | after delivery, miliary  tuberculosis |
| 5CJ | 1 | 35.86 | 2100 | 0 | 0 | 24 | 35 | Persistent fever resistant to  antibiotic  therapy |  |  | √ | √  (BALF) | - | 35 | - | a mass in the   right neck after delivery | after delivery, chest radiography miliary tuberculosis |
| 6XJH | 1 | 35.00 | 2100 | 0 | 0 | 15 | 30 | Deteriorationeven after  intubation,  resistant to antibiotic therapy | √（sputum） | √ |  |  | - | 31 | 31 | hyperpyrexia before and  after delivery | after delivery, chest radiography miliary tuberculosis |
| 7ZLE | 0 | 32.14 | 1740 | 0 | 1, Tubal tuberculosis | 29 | 31 | Deterioration  even after  intubation,  resistant to  antibiotic therapy | √（sputum) | √ |  |  | - | 32 | - | fever during  delivery | Before delivery, tubal tuberculosis |
| 8TMQ | 1 | 29.71 | 1350 | 1 | 1 | 35 | 45 | Deterioration  even after   intubation,  resistant to  antibiotic therapy | √（sputum |  |  |  | - | 45 | 54 | fever, cough   and headache before  delivery | after delivery, chest radiography miliary tuberculosis |
| 9QJ | 0 | 30.43 | 1430 | 1 | 1, Tubal tuberculosis | 47 | 50 | Deterioration  even after  intubation,  resistant to  antibiotic therapy | √  (BALF) | √ |  |  | - | 50 | 57 | fever before  delivery | Before delivery,tubal  tuberculosis |

GA,gestational age; BW, birth weight; CS, Caesarean section;IVF, in-vitro fertilization;,PCR,Polymerase Chain Reaction; IGRA,Interferon Gamma Release Assay;NGS, Next Generation Sequencing; DAB, days after birth; BALF, Bronchoalveolar Lavage Fluid.
