# Supplementary material for: Immunopathological signatures in congenital tuberculosis-a case-matched study
Source: Front Immunol. 2026 Mar 30;17:1614510. doi: 10.3389/fimmu.2026.1614510 (PMC13070812; doi:10.3389/fimmu.2026.1614510)
Supplement: Supplementary file 3 [file Table2.docx]

| Patients | Groups | Gender  (male=0,  female=1) | GA (weeks) | BW  (g) | Delivery  (CS=1,natural delivery=0) | Apgars  Score 1minute | Apgars  Score  5 minute | Respiratory support  on admission | Sepsis evaluation | Antibiotics therapy duration, days (for CTB-before TB diagnosis) | BPD |
| --- | --- | --- | --- | --- | --- | --- | --- | --- | --- | --- | --- |
| 1LQY | HC | 0 | 24.00 | 610 | 0 | 9 | 10 | Invasive | No sepsis | 7 | Yes, mild |
| 1DHL | CTB | 1 | 27.00 | 1060 | 1 | 9 | 10 | Invasive | Mtb | 13 | no |
| 2ZL | HC | 1 | 35.85 | 2120 | 0 | 9 | 10 | No support | No sepsis | 0 | no |
| 2YJZ | CTB | 1 | 34.00 | 2400 | 0 | 9 | 10 | No support | Mtb | 18 | no |
| 3ZSH | HC | 0 | 28.31 | 1040 | 0 | 8 | 10 | NCPAP | No sepsis | 10 | no |
| 3CDN | CTB | 1 | 31.57 | 1540 | 0 | 9 | 10 | NCPAP | Mtb | 20 | Yes, mild |
| 4HJY | HC | 1 | 32.14 | 1030 | 1 | 9 | 10 | NCPAP | No sepsis | 5 | no |
| 4ZJJ | CTB | 1 | 33.43 | 1220 | 1 | 9 | 10 | NCPAP | Mtb | 7 | no |
| 5LJR | HC | 1 | 37.14 | 2027 | 1 | 10 | 10 | No support | No sepsis | 0 | no |
| 5CJ | CTB | 1 | 35.86 | 2100 | 0 | 10 | 10 | No support | Mtb | 15 | no |
| 6RY | HC | 1 | 33.26 | 1500 | 1 | 10 | 10 | No support | No sepsis | 0 | no |
| 6XJH | CTB | 1 | 35.00 | 2100 | 0 | 10 | 10 | No support | Mtb | 16 | no |
| 7LHM | HC | 0 | 31.00 | 1460 | 1 | 8 | 10 | NCPAP | No sepsis | 3 | no |
| 7ZLE | CTB | 0 | 32.14 | 1740 | 0 | 9 | 10 | NCPAP | Mtb | 10 | no |
| 8HXL | HC | 1 | 28.86 | 1170 | 0 | 9 | 10 | NCPAP | No sepsis | 3 | Yes, severe |
| 8TMQ | CTB | 1 | 29.71 | 1350 | 1 | 8 | 10 | NCPAP | Mtb | 18 | Yes, severe |
| 9CC | HC | 0 | 27.71 | 1130 | 0 | 9 | 10 | NCPAP | No sepsis | 5 | Yes, moderate |
| 9QJ | CTB | 0 | 30.43 | 1430 | 1 | 8 | 10 | NCPAP | Mtb | 18 | Yes, severe |
| P value | - | 0.620 | 0.480 | 0.145 | 1.000 | 1.000 | 1.000 | 1.000 | - | **0.001** | 1.000 |

Supplemental table 2. Neonatal baseline characteristics of the CTB and pHC infants. GA,gestational age; BW, birth weight; CS,Caesarean section; NCPAP, non-invasive Continuos Positive Airway Pressure; M.tb, Mycobacterium tuberculosis; BPD,bronchopulmonary dysplasia.

Group comparisons of categorical variables were performed using the Fisher’s exact test. For continuous variables, non-parametric analysis Mann-Whitney U was used for statistical tests.
