# Supplementary material for: Immunopathological signatures in congenital tuberculosis-a case-matched study
Source: Front Immunol. 2026 Mar 30;17:1614510. doi: 10.3389/fimmu.2026.1614510 (PMC13070812; doi:10.3389/fimmu.2026.1614510)
Supplement: Supplementary file 4 [file Table3.docx]

| **Patients** | **Groups** | **Days after birth** | | | | | | | | | | |
| --- | --- | --- | --- | --- | --- | --- | --- | --- | --- | --- | --- | --- |
| 1LQY | HC1 |  | 2 | 3 | 6 | 11 | 16 | 17 | 20 | 25 | 30 | 37 |
| 1DHL | TB1 |  |  | 1 | 4 |  | 14 | 15 | 18 | 20 |  |  |
| 2ZL | HC2 |  | 5 | 7 | 11 | 12 | 18 | 27 | 32 |  |  |  |
| 2YJZ | TB2 |  |  | 15 | 18 |  | 22 | 23 | 25 |  |  |  |
| 3ZSH | HC3 | 1 | 13 |  |  | 33 | 42 |  | 50 | 51 | 52 | 53 |
| 3CDN | TB3 | 10 | 20 | 25 | 26 | 39 | 42 | 44 | 49 | 53 | 54 |  |
| 4HJY | HC4 | 1 | 6 | 8 | 12 | 19 | 20 | 22 | 26 |  |  | 32 |
| 4ZJJ | TB4 | 1 | 2 | 6 | 14 | 19 | 21 | 24 | 26 | 27 | 29 | 31 |
| 5LJR | HC5 |  |  |  |  |  | 24 |  | 31 | 35 |  |  |
| 5CJ | TB5 |  |  |  |  |  | 24 | 27 | 31 |  |  |  |
| 6RY | HC6 |  |  |  |  |  |  |  |  | 21 | 24 | 31 |
| 6XJH | TB6 |  |  |  |  |  |  |  |  | 28 | 29 | 33 |
| 7LHM | HC7 |  |  |  |  |  | 29 | 32 | 37 |  |  |  |
| 7ZLE | TB7 |  |  |  |  |  | 30 | 31 | 32 |  |  |  |
| 8HXL | HC8 | 1 | 6 | 12 | 19 | 23 | 30 | 40 | 45 |  |  | 51 |
| 8TMQ | TB8 | 7 | 10 | 19 | 25 | 30 | 35 | 37 | 40 | 42 | 43 | 45 |
| 9CC | HC9 |  |  |  |  |  |  | 48 | 53 | 58 |  |  |
| 9QJ | TB9 |  |  |  |  |  |  | 48 | 49 | 51 |  |  |


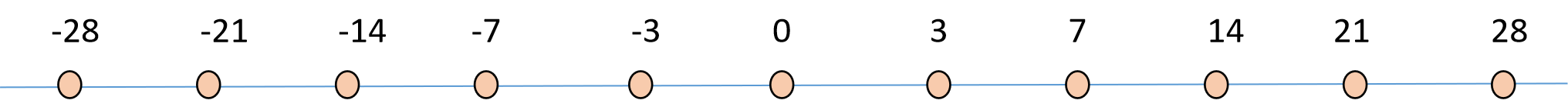


Supplemental table 3. The dynamic detection time point of routine blood tests.
