# Supplementary material for: Immunopathological signatures in congenital tuberculosis-a case-matched study
Source: Front Immunol. 2026 Mar 30;17:1614510. doi: 10.3389/fimmu.2026.1614510 (PMC13070812; doi:10.3389/fimmu.2026.1614510)
Supplement: Supplementary file 5 [file Table4.docx]

| Variable | βGroup  CTB VS HC | P | β (Days) | P | β(Days×Group) | P |
| --- | --- | --- | --- | --- | --- | --- |
| WBC | 2.931 | 0.006 | 0.0524 | 0.345 | 0.743 | 0.333 |
| L% | -10.483 | <0.001 | 0.333 | 0.009 | -0.849 | **<0.001** |
| N% | 7.677 | 0.012 | -0.525 | 0.001 | 1.067 | **<0.001** |
| MN% | -1.208 | 0.128 | 0.079 | 0.054 | -0.190 | **0.001** |
| CRP | 32.71 | <0.001 | -0.348 | 0.001 | 2.886 | **<0.001** |
| PLT | -31.669 | 0.269 | 2.113 | 0.154 | -6.570 | **0.002** |

Supplemental table 4.Longitudinal Changes in peripheral blood parameters in congenital tuberculosis (CTB) and healthy control (HC) neonates analyzed by

linear mixed-effects model.

WBC, white blood cell count; L%, lymphocyte percentage; N%, neutrophil percentage; MN%, monocyte percentage; CRP, C-reactive protein; PLT, platelet count; β, beta coefficient.

Analyses were performed using linear mixed-effects models with patient ID as a random intercept to account for intra-individual correlation in repeated measurements.
