# Supplementary figures and images for: Immunopathological signatures in congenital tuberculosis-a case-matched study
Source: Front Immunol. 2026 Mar 30;17:1614510. doi: 10.3389/fimmu.2026.1614510 (PMC13070812; doi:10.3389/fimmu.2026.1614510)

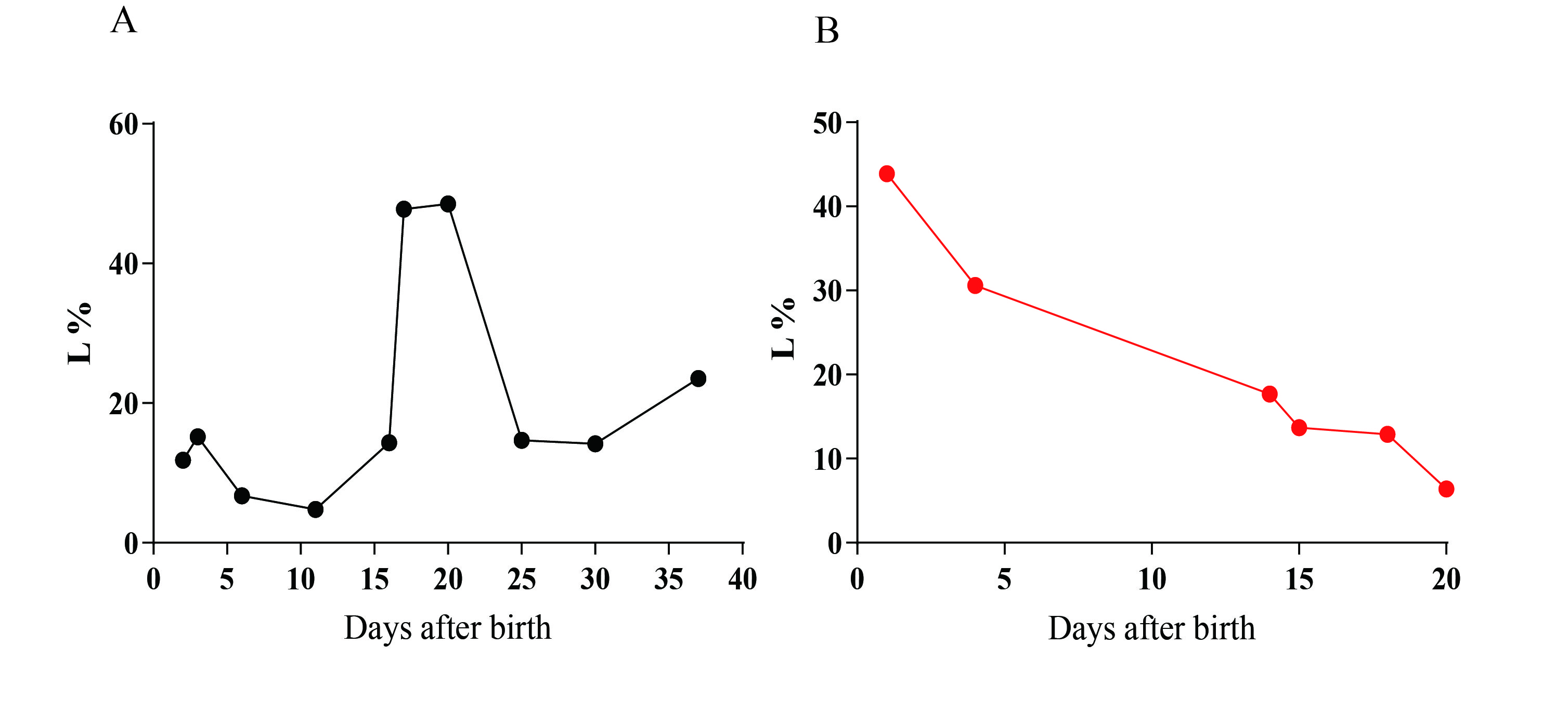

Supplement: Supplementary Figure 2 — The dynamic features of lymphocytes frequency in two patients conducting single-cell seq. (A) control patient. (B) CTB patient. [file Image2.jpeg]

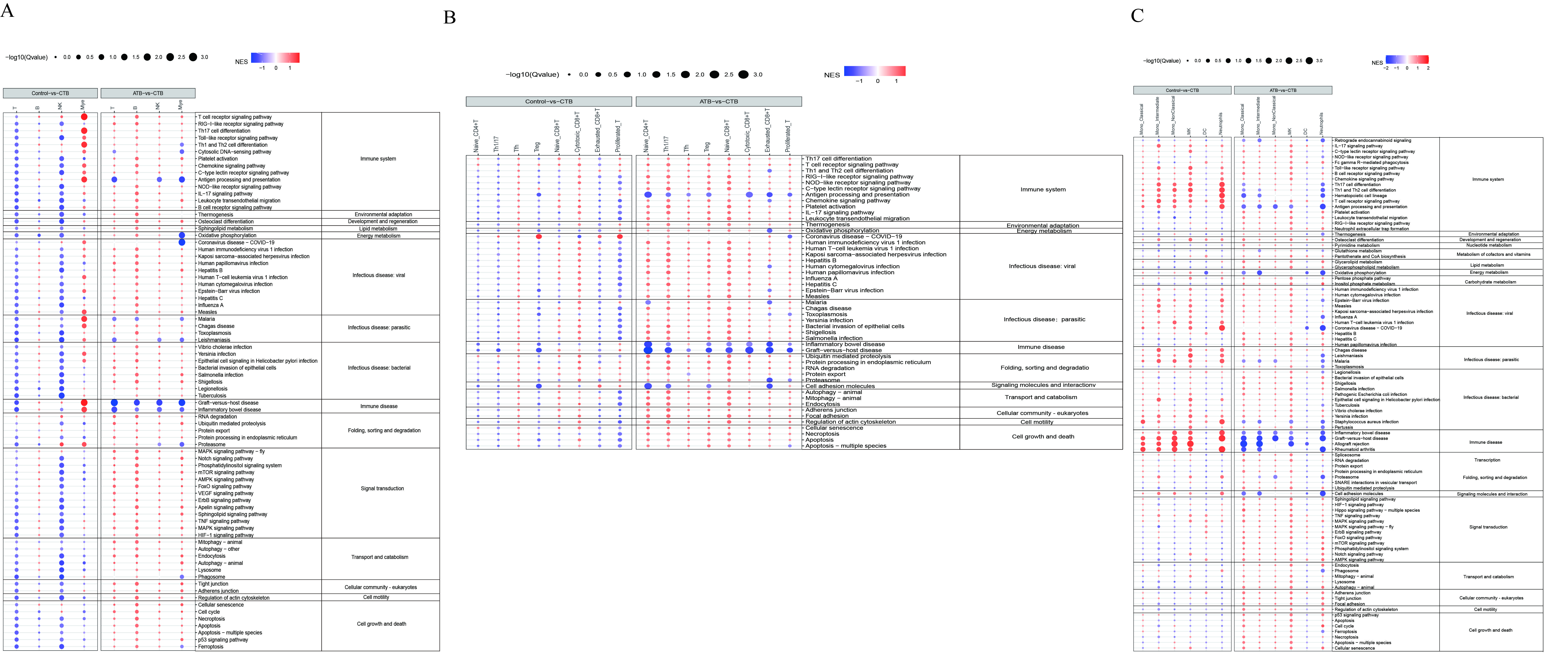

Supplement: Supplementary Figure 3 — GSEA of Control vs CTB and ATB vs CTB in main cell types of PBMC (A), myeloid cells (B) and T cells subsets (C). Selected DEGs sets are grouped into functional/pathway categories. Dot color denotes normalized gene set enrichment score, and size indicates-log10 (adjusted P value). P values were from GSEA test of the whole gene sets (Methods) and adjusted using the Benjamini–Hochberg method. [file Image3.jpeg]

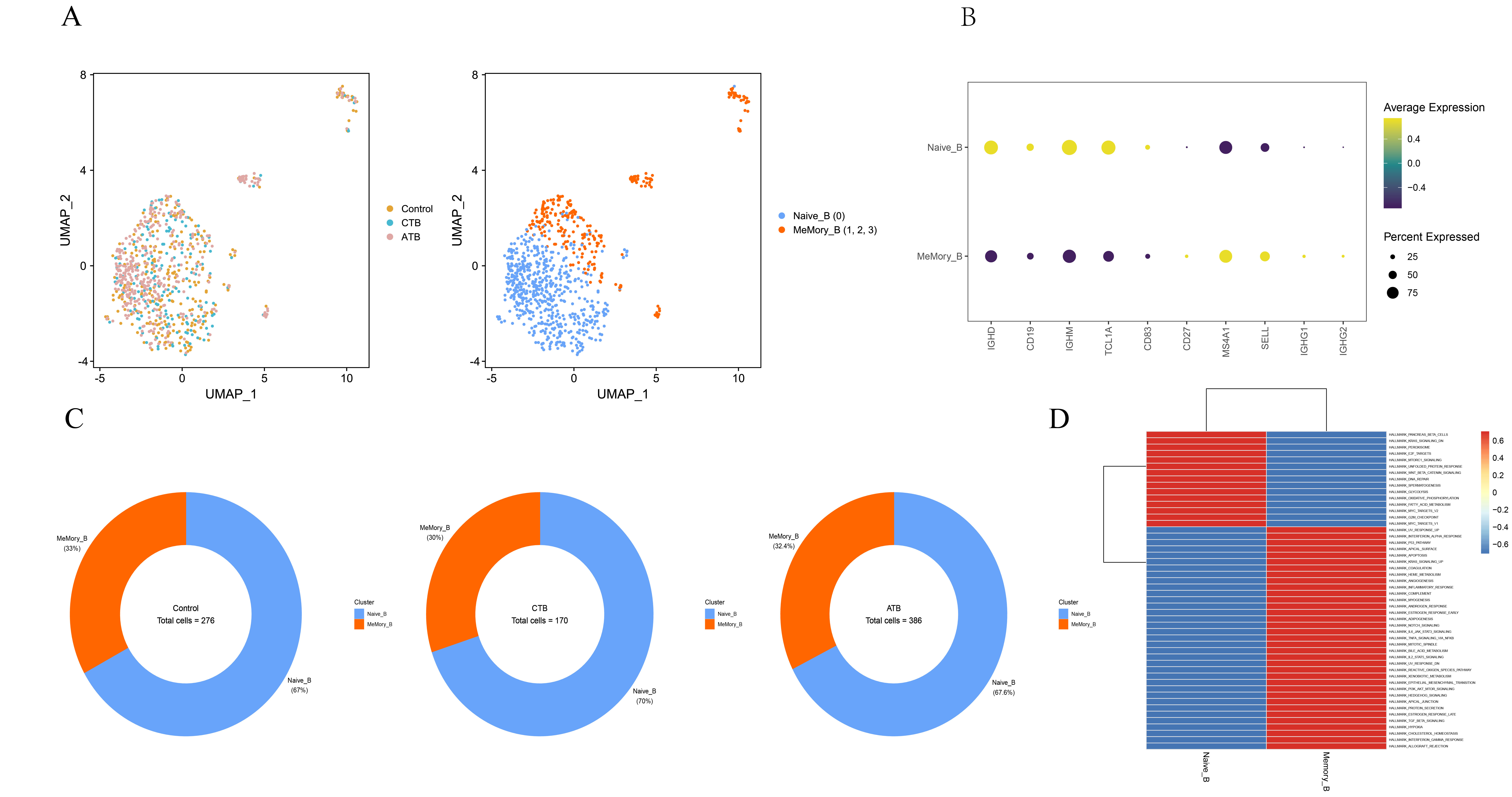

Supplement: Supplementary Figure 4 — Single-cell transcriptional profiling of B cells from HC, CTB and ATB. (A) UMAP of single cell profile with each cell color-coded for sample type and associated cell type. (B) Dot plot showing expression of B cell markers. Dot plot color gradient represents the average gene expression scale. Dot size (pct.exp) represents the proportion of cells expressing the corresponding gene in the cell type. The larger the dot, the higher the proportion of cells expressing the gene. (C) The fraction of B cells subset in HC, CTB and ATB. (D) Differences in pathway activities scored per cell by GSVA between the different B cell subsets. The color denotes normalized gene set enrichment score (NGS). [file Image4.jpeg]
