# Supplementary material for: Immunopathological signatures in congenital tuberculosis-a case-matched study
Source: Front Immunol. 2026 Mar 30;17:1614510. doi: 10.3389/fimmu.2026.1614510 (PMC13070812; doi:10.3389/fimmu.2026.1614510)
Supplement: Supplementary File 1 — 1.1 The representative gating strategy figure of Lymphocyte immune cells flow-cytometric analysis; 1.2 UMAP plots before and after integration; 1.3 The custom R processing scripts. [file Table15.docx]

Supplemental file 1.

1.1 The representative gating strategy figure of Lymphocyte immune cells flow-cytometric analysis.

The gating strategy for immunophenotypic analysis of lymphocytes was performed as follows:

· Initial lymphocyte gating: Lymphocyte populations were first gated based on forward scatter (FSC) and side scatter (SSC) properties to exclude cell debris, dead cells, and non-lymphoid cellular contaminants.

· Lymphocyte confirmation with CD45: The gated lymphocyte fraction was further refined by selecting CD45-positive (CD45+) cells, a specific marker for leukocytes, to confirm the lymphocyte population.

· T cell subset gating: Within the CD45+ lymphocyte population, CD3-positive (CD3+) cells were gated to identify total T cells. CD3+ T cells were subsequently subdivided into CD4+ single-positive, CD8+ single-positive, and CD4+CD8+ double-positive subsets based on the expression of CD4 and CD8 markers (as shown in the "CD3/CD8/CD45/CD4 TruC" panel).

· NK and B cell gating: In the CD3-negative (CD3−) lymphocyte fraction, CD16+CD56+ cells were gated to identify natural killer (NK) cells, and CD19+ cells were gated to identify B cells (as shown in the "CD3/CD16+56/CD45/CD19 TruC" panel).


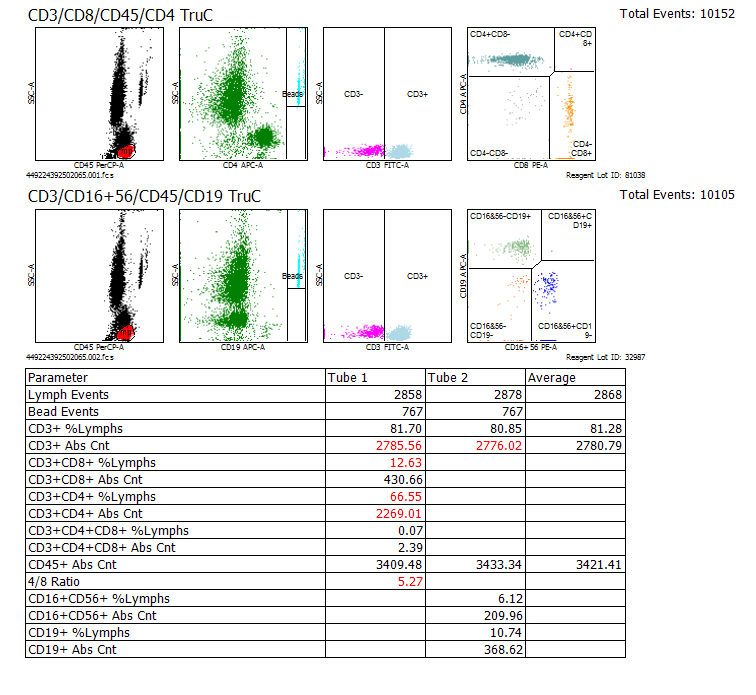


T cell subset gating


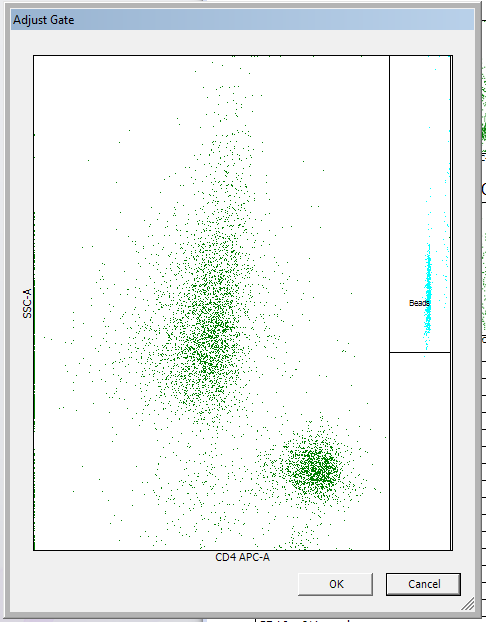

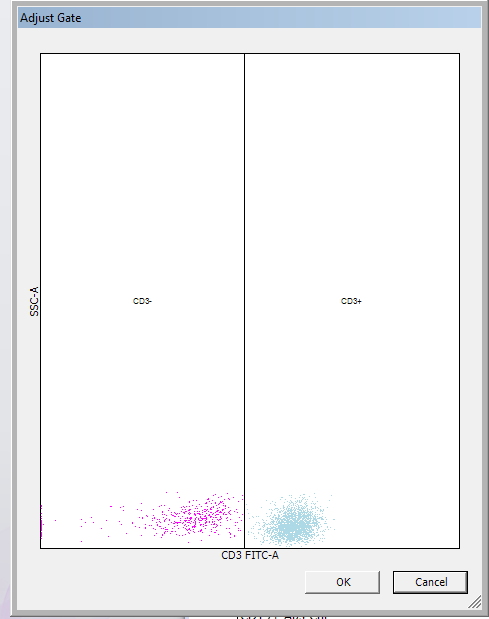


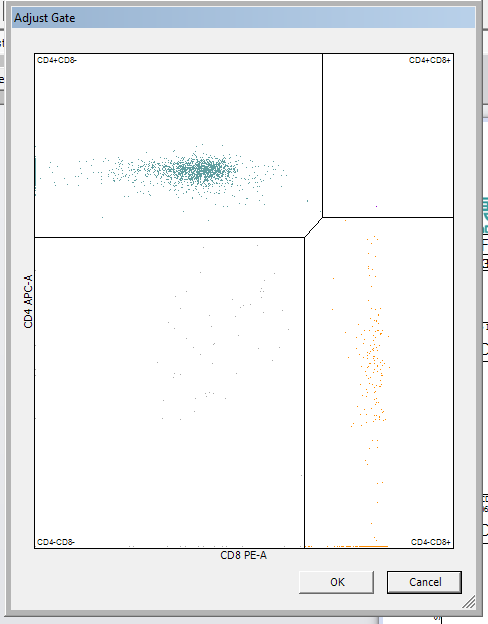


NK and B cell gating:


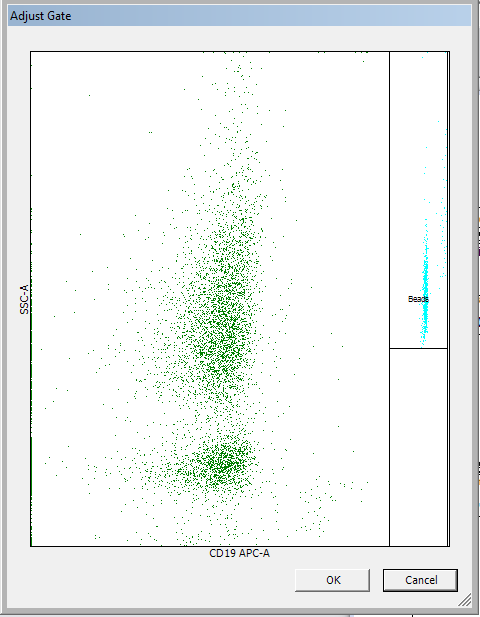

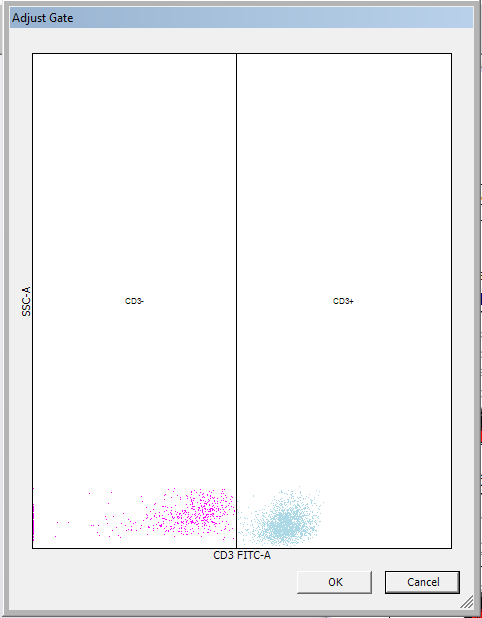


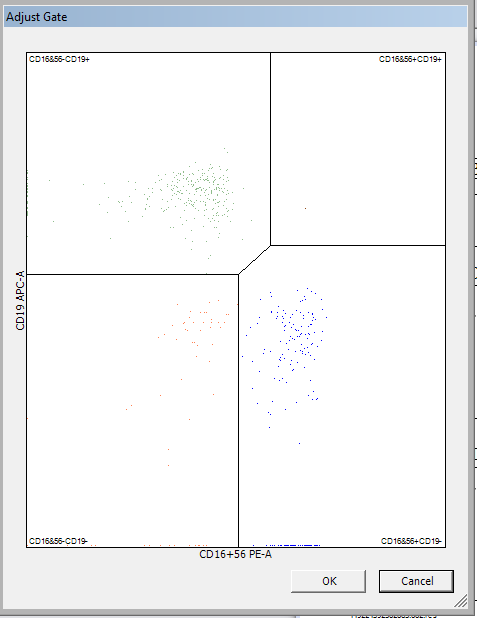


Gating Strategy for CD4+CD25+Foxp3+ Regulatory T (Treg) Cell Analysis.

The gating strategy for identifying CD4+CD25+Foxp3+ Treg cells via flow cytometry was implemented in a hierarchical manner to ensure precise enrichment of the target cell subset.

Negative controls were used to empirically determine and set the boundaries for Foxp3 and CD25 positivity in the flow cytometry analysis.

Single nucleated cell gating: First, forward scatter height (FSC-H) vs. forward scatter area (FSC-A) and side scatter area (SSC-A) vs. FSC-A dot plots were used to gate single nucleated cells, excluding cell debris, cell aggregates, and dead cells to ensure the integrity of the analyzed cell population.

Leukocyte confirmation with CD45:Within the gated single nucleated cell fraction, CD45-positive (CD45+) cells were selected to identify leukocytes, eliminating non-hematopoietic cells from the analysis.

CD4+ T cell enrichment:From the CD45+ leukocyte population, CD4-positive (CD4+) T cells were gated using CD4 expression, excluding other immune cell subsets (e.g., CD8+ T cells, NK cells, B cells).

CD4+CD25+ cell subset selection:In the CD4+ T cell fraction, CD25-positive (CD25+) cells were further gated to isolate the CD4+CD25+ cell subset, a surface marker-enriched population for Treg cells.

Foxp3+ Treg cell identification:Finally, within the CD4+CD25+ cell subset, Foxp3-positive (Foxp3+) cells were gated (Q1 quadrant in the Foxp3 PE-A vs. CD4 PerCP-A dot plot) to define the final CD4+CD25+Foxp3+ Treg cell population, the key target subset for this analysis.


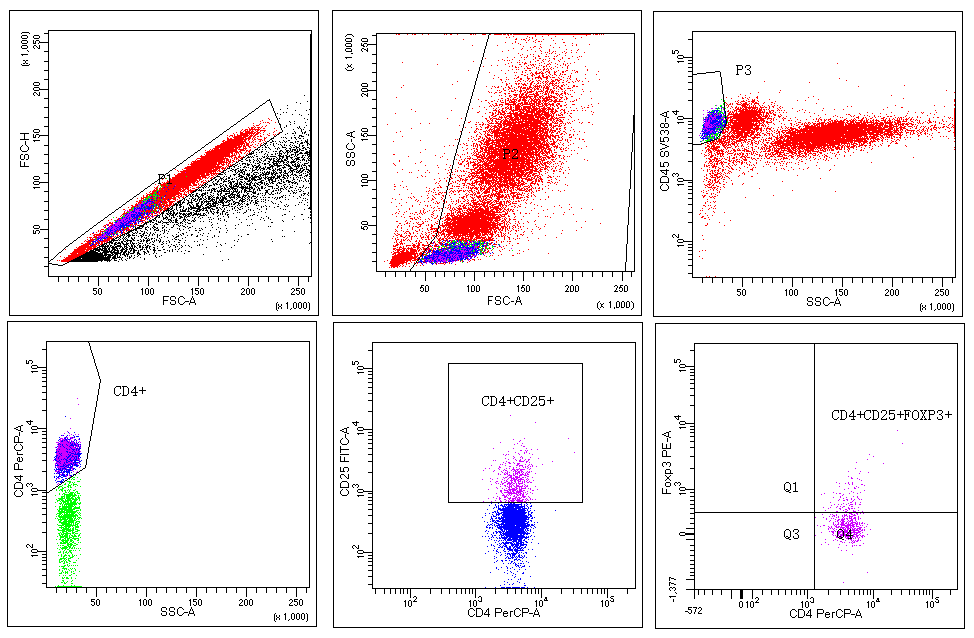


1.2 UMAP plots before and after integration

Before integration

Control


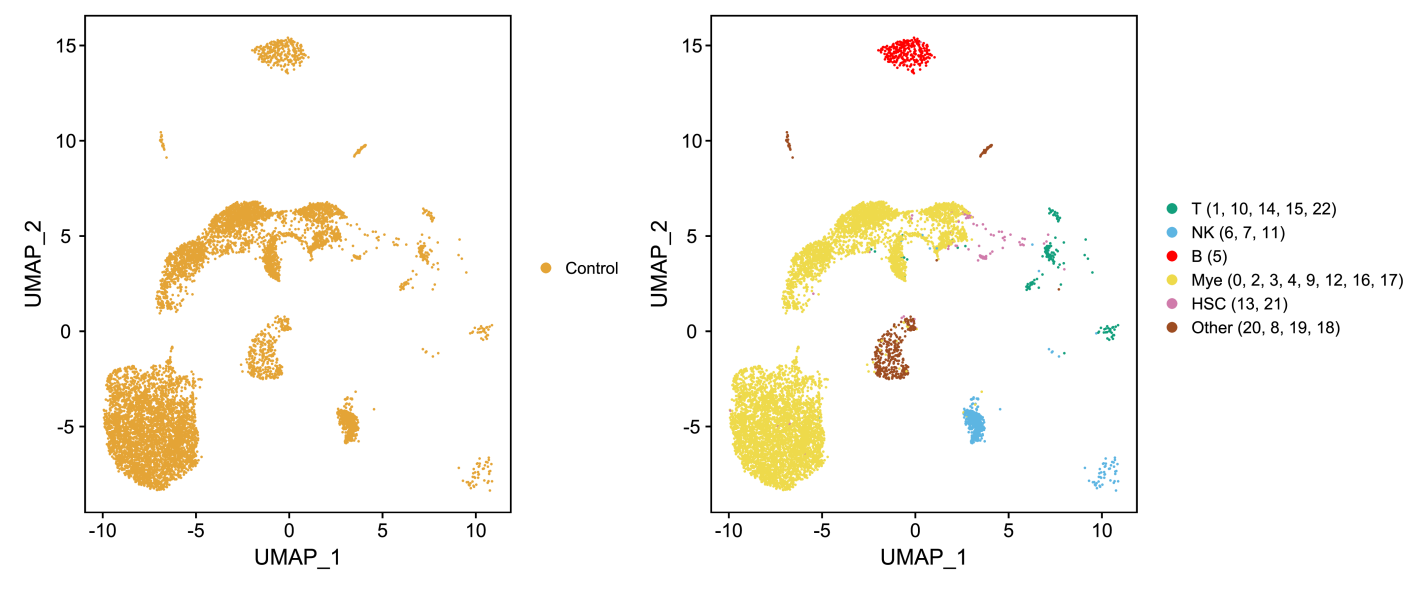


CTB


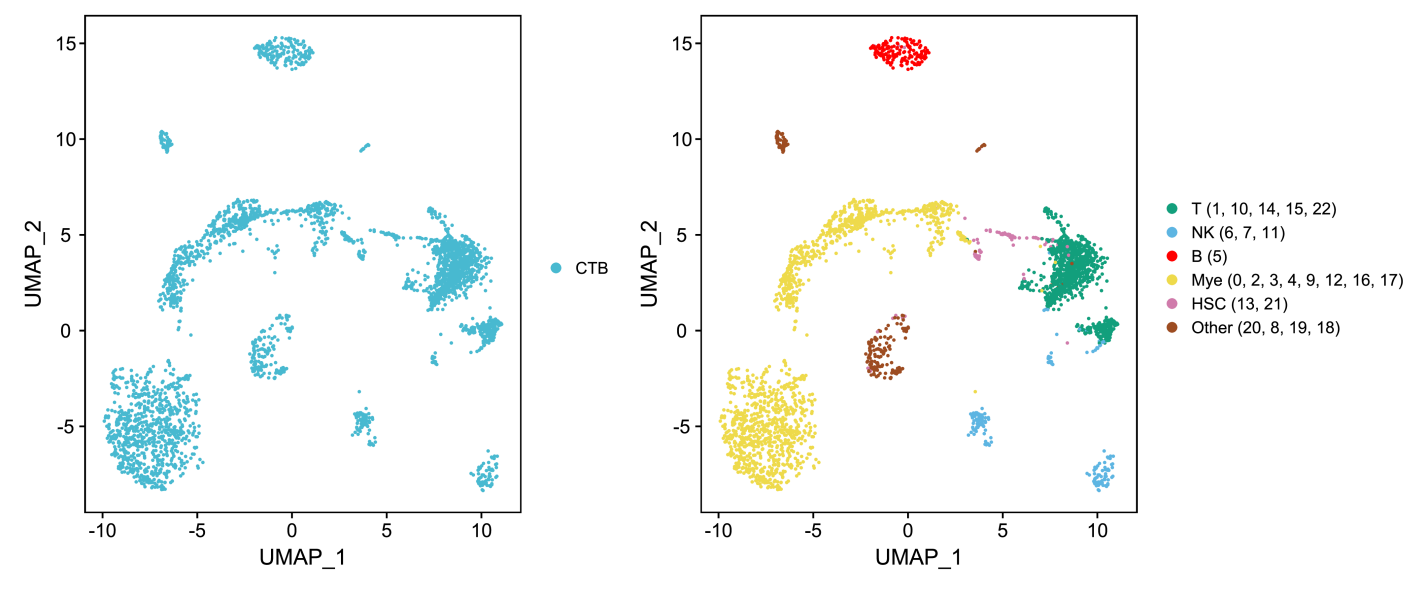


ATB


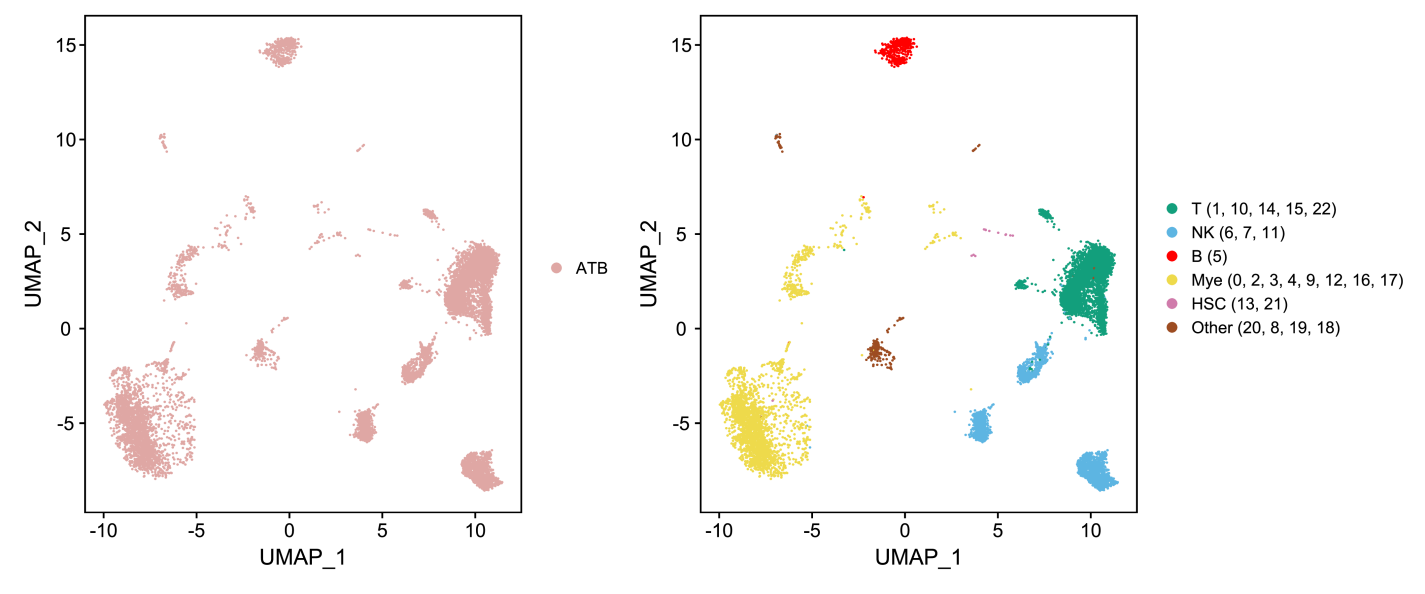


After integration


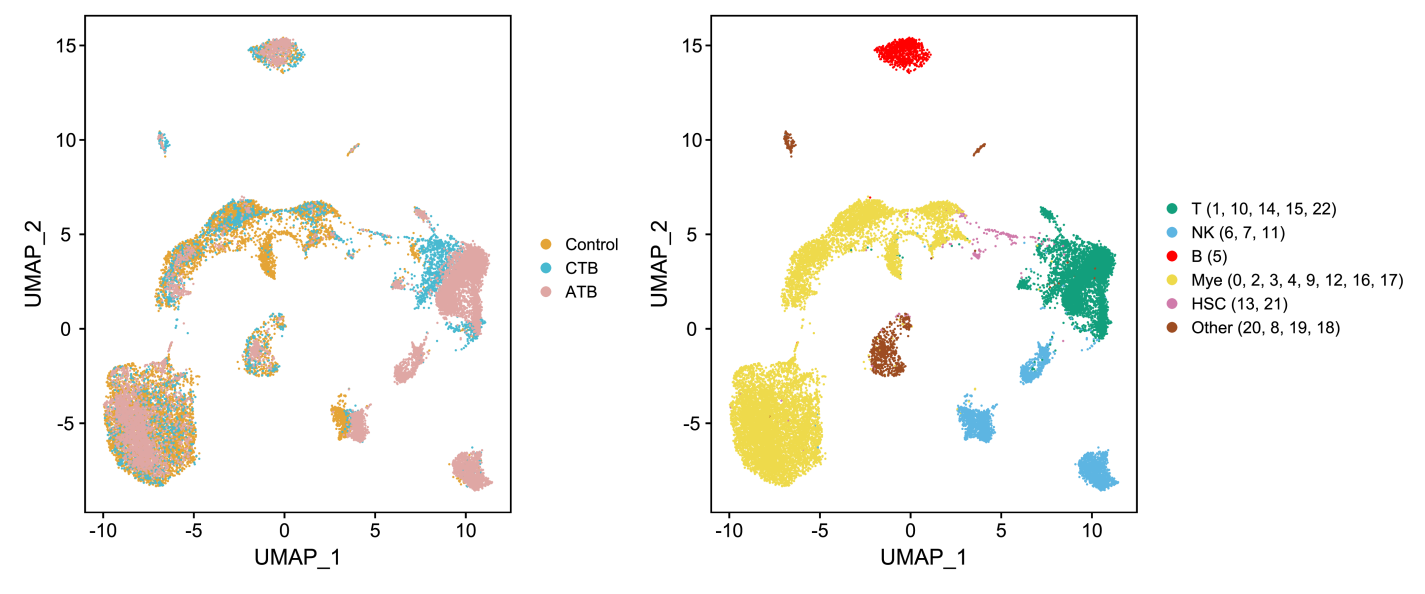


1.3 The custom R processing scripts.

Cell ranger

export SGE_CLUSTER_NAME=SGE; cd pipe_out/1.CellRanger/Samples; cellranger count --id Ctrl-1 --transcriptome GRCh38_annot/ --disable-ui --fastqs upload/Ctrl-1 --sample Ctrl-1 --force-cells 8571 --chemistry SC3Pv3 --include-introns --jobmode sge --maxjobs 8 --mempercore 6

export SGE_CLUSTER_NAME=SGE; cd pipe_out/1.CellRanger/Samples; cellranger count --id Ctrl-2 --transcriptome GRCh38_annot/ --disable-ui --fastqs upload/Ctrl-2 --sample Ctrl-2 --expect-cells 3000 --chemistry SC3Pv3 --include-introns --jobmode sge --maxjobs 5 --mempercore 6

export SGE_CLUSTER_NAME=SGE; cd pipe_out/1.CellRanger/Samples; cellranger count --id Ctrl-3 --transcriptome GRCh38_annot/ --disable-ui --fastqs upload/Ctrl-3 --sample Ctrl-3 --expect-cells 3000 --chemistry SC3Pv3 --include-introns --jobmode sge --maxjobs 5 --mempercore 6

export SGE_CLUSTER_NAME=SGE; cd pipe_out/1.CellRanger/Samples; cellranger count --id EM-1 --transcriptome GRCh38_annot/ --disable-ui --fastqs upload/EM-1 --sample EM-1 --expect-cells 3000 --chemistry SC3Pv3 --include-introns --jobmode sge --maxjobs 5 --mempercore 6

export SGE_CLUSTER_NAME=SGE; cd pipe_out/1.CellRanger/Samples; cellranger count --id EM-2 --transcriptome GRCh38_annot/ --disable-ui --fastqs upload/EM-2 --sample EM-2 --expect-cells 3000 --chemistry SC3Pv3 --include-introns --jobmode sge --maxjobs 5 --mempercore 6

export SGE_CLUSTER_NAME=SGE; cd pipe_out/1.CellRanger/Samples; cellranger count --id EM-3 --transcriptome GRCh38_annot/ --disable-ui --fastqs upload/EM-3 --sample EM-3 --expect-cells 3000 --chemistry SC3Pv3 --include-introns --jobmode sge --maxjobs 5 --mempercore 6

Doublet

### Deal arguements

args <- commandArgs(T)

indir <- args[1]

sample <- args[2]

add_lib <- args[3]

outdir <- args[4]

rate <- args[5]

if ( is.null( file ) | is.na( file ) ){

warning( "\n Usage : Seurat.R <parameter.yaml> (<outdir>)\n" )

quit()

}

library(Seurat)

library(dplyr)

library(ggplot2)

library(patchwork)

library(DoubletFinder)

library(future)

options(future.globals.maxSize = 100 * 1024 * 1024^2)

plan("multiprocess", workers = 4)

#plan("sequential")

source(add_lib, chdir = TRUE)

### Let's shake it

if ( ! is.na(outdir) ) setwd(outdir)

### Creat Seurat Object

message( "==>Reading 10x data<==" )

obj <- MakeSeuratObj(data_name = sample, data_dir = indir)

### Normalization Data

message( "==>Normalization Data<==" )

obj <- DoNormalization(obj, vars.regress = "none", is_SCTransform = FALSE, is.check = FALSE, scale.only.var.genes = TRUE)

### Reduce dimension

message( "==>Reduce dimension<==" )

obj <- DoDimReduc(obj, is.checkpca = FALSE, check_duplicates = FALSE)

dims <- seq(obj@reductions$pca)

### get pN

pN <- 0.25

### get pK

sweep.res.list <- paramSweep_v3(obj, PCs = dims, sct = FALSE)

sweep.stats <- summarizeSweep(sweep.res.list)

bcmvn <- find.pK(sweep.stats)

pK <- as.numeric(as.vector(bcmvn$pK[which.max(bcmvn$BCmetric)[1]]))

pdf(paste0("pK.", sample, ".pdf"))

plot(x = as.vector(bcmvn$pK), y = bcmvn$BCmetric, type = "b", xlab = "pK", ylab = "BCmetric", col = "blue", pch = 19)

abline(v = pK, lty = 2, col = "red")

dev.off()

WriteTable(bcmvn, file = paste0("pK.", sample, ".xls"))

### get nExp

if ( is.na(rate) )

rate <- 7.6 * 10^-6 * ncol(obj) + 5.27 * 10^-4

nExp_poi <- round(as.numeric(rate) * ncol(obj))

if( exists("seurat_clusters", obj@meta.data) ){

annotations <- obj@meta.data$seurat_clusters

homotypic.prop <- modelHomotypic(annotations)

nExp_poi <- round(nExp_poi * (1 - homotypic.prop))

}

## Run DoubletFinder with varying classification stringencies ----------------------------------------------------------------

obj <- doubletFinder_v3(obj, PCs = dims, pN = pN, pK = pK, nExp = nExp_poi, reuse.pANN = FALSE, sct = FALSE)

colnames(obj@meta.data)[grep('pANN', colnames(obj@meta.data))] <- "pANN"

colnames(obj@meta.data)[grep('DF.classifications', colnames(obj@meta.data))] <- "classifications"

embeddings <- cbind(obj@reductions[["tsne"]]@cell.embeddings, obj@reductions[["umap"]]@cell.embeddings)

data <- obj@meta.data[,c("pANN", "classifications")]

WriteTable(cbind(Cells = rownames(data), data), file = paste0("DF.classify.", sample, ".xls"))

WriteTable(cbind(Cells = rownames(data), data, embeddings), file = paste0("DF.classify.", sample, ".tmp"))

p1 <- DimPlot(obj, reduction = "umap", group.by = "classifications", cols = c("Singlet" = "black", "Doublet" = "red")) + dot_theme_default() + ggtitle(NULL)

ggsave(p1, file = paste0("DF.classify.UMAP.", sample, ".pdf"), width = 6, height = 5)

p2 <- DimPlot(obj, reduction = "tsne", group.by = "classifications", cols = c("Singlet" = "black", "Doublet" = "red")) + dot_theme_default() + ggtitle(NULL)

ggsave(p2, file = paste0("DF.classify.tSNE.", sample, ".pdf"), width = 6, height = 5)

PlotFeaturePlot(obj, "pANN", 'umap', outfile = paste0("pANN.UMAP.", sample, ".pdf"))

PlotFeaturePlot(obj, "pANN", 'tsne', outfile = paste0("pANN.tSNE.", sample, ".pdf"))

p <- ggplot(obj@meta.data, aes(x = pANN)) + geom_histogram() + bar_theme_default()

ggsave(p, file = paste0("pANN.hist.", sample, ".pdf"), width = 7, height = 7)

PlotBasicStat(obj, "BasicInfo", group.point.by = "classifications", group.point.color = c("Singlet" = "black", "Doublet" = "red"))

Seurat.R

### Deal arguements

args <- commandArgs(T)

file <- args[1]

outdir <- args[2]

add_lib <- args[3]

if ( is.null( file ) | is.na( file ) ){

warning( "\n Usage : Seurat.R <parameter.yaml> (<outdir>)\n" )

quit()

}

### Loading Library

handlers <- list("bool#no" = function(x){if ( x %in% c("false", "FALSE") ) FALSE else x}, "bool#yes" = function(x){if ( x %in% c("true", "TRUE") ) TRUE else x})

parameter <- yaml::yaml.load_file( file, handlers = handlers)

library(Seurat)

library(dplyr)

library(ggplot2)

library(patchwork)

library(harmony)

library(future)

options(future.globals.maxSize = 100 * 1024 * 1024^2)

plan("multiprocess", workers = 4)

#plan("sequential")

#source("/home/xushuyang/Pipeline/R/SCellWare/R/Seurat_lib.R", chdir = T)

if ( ! is.na(add_lib) ) source(add_lib, chdir = T)

### Let's shake it

if ( ! is.na(outdir) ) setwd(outdir)

### Creat Seurat Object

message( "==>Reading 10x data<==" )

obj <- MakeSeuratObj(parameter)

obj <- OverrideFeatures(obj, parameter)

### Add some check flag

message( "==>Adding MetaData<==" )

if ( ! is.null(parameter$marker$mito_list) ) obj <- StatFeatures(obj, parameter$marker$mito_list, col.name = "percent.mito", stat_pct = T, add_to_pdata = T)

if ( ! is.null(parameter$marker$plastid_list) ) obj <- StatFeatures(obj, parameter$marker$plastid_list, col.name = "percent.plastid", stat_pct = T, add_to_pdata = T)

if ( ! is.null(parameter$marker$expected) ) obj <- StatFeatures(obj, parameter$marker$expected, col.name = "expected.marker")

if ( ! is.null(parameter$marker$excluded) ) obj <- StatFeatures(obj, parameter$marker$excluded, col.name = "exclude.marker")

if ( ! is.null(parameter$marker$more) ) obj@misc[["more.marker"]] <- FindFeaturesID(obj, parameter$marker$more, unlist = FALSE)

WriteTable(tibble::rownames_to_column(obj@meta.data, var = "Cells"), file = "metadata.xls")

### Data Stat - before filter

message( "==>Stat before BasicInfo<==" )

if ( is.null(parameter$filter$filter.cells) ) {

PlotBasicStat(obj, "BasicInfo", nRow = 1)

if ( ! is.null(parameter$Groups) ) {

PlotBasicStat(obj, "BasicInfo.groups", group.by = "Groups", nRow = 1)

}

} else {

obj[["DF"]] <- "Singlet"

filter.cells <- readLines(parameter$filter$filter.cells)

obj@meta.data[filter.cells, "DF"] <- "Doublet"

PlotBasicStat(obj, "BasicInfo", group.point.by = "DF", group.point.color = c("Singlet" = "black", "Doublet" = "red"), nRow = 1)

if ( ! is.null(parameter$Groups) ) {

PlotBasicStat(obj, "BasicInfo.groups", group.by = "Groups", group.point.by = "DF", group.point.color = c("Singlet" = "black", "Doublet" = "red"), nRow = 1)

}

}

### Filter

message( "==>Filter<==" )

obj <- FilterGenes(obj, parameter)

obj <- FilterCells(obj, parameter, do.stat = FALSE)

StatFilterCells(obj, group.by = "orig.ident", outfile = "Filter.stat.xls")

if ( ! is.null(parameter$Groups) ) {

StatFilterCells(obj, group.by = "Groups", outfile = "Filter.stat.groups.xls")

}

### Data Stat - after filter

message( "==>Stat after BasicInfo<==" )

PlotBasicStat(obj, "AfterFilter.BasicInfo", nRow = 1)

if ( ! is.null(parameter$Groups) ) {

PlotBasicStat(obj, "AfterFilter.BasicInfo.groups", group.by = "Groups", nRow = 1)

}

### Normalization Data

message( "==>Normalization Data<==" )

obj <- DoNormalization(obj, parameter, is_SCTransform = FALSE,

scale.only.var.genes = TRUE,

vfeature.must = obj@misc[['expected.marker']],

vfeature.remove = obj@misc[['exclude.marker']])

### Reduce dimension

message( "==>Reduce dimension<==" )

obj <- DoDimReduc(obj)

### Find clusters

message( "==>Find clusters<==" )

obj <- DoFindClusters(obj, reduction = "pca", dims = NULL, resolution = parameter$cluster_resolution)

if ( ! is.null(parameter$integration$method) ) {

if ( length(table(obj[["orig.ident"]])) > 1 ) {

## check before integration visualization

obj[["beforeInteg.cluster"]] <- Idents(object = obj)

PlotCluster(obj, reduction = 'umap_RNA', outpref = "UMAP_before" )

PlotCluster(obj, reduction = 'tsne_RNA', outpref = "tSNE_before" )

if ( ! is.null(parameter$Groups) ) {

PlotCluster(obj, reduction = 'umap_RNA', outpref = "UMAP_before.groups", split.by = "Groups", p1.group.by = "Groups" )

PlotCluster(obj, reduction = 'tsne_RNA', outpref = "tSNE_before.groups", split.by = "Groups", p1.group.by = "Groups" )

}

### Integration

message( "==> Do Integration <==" )

if ( parameter$integration$method == "CCA" ) {

obj <- DoIntegration(obj, split.by = "orig.ident")

obj <- DoDimReduc(obj)

obj <- DoFindClusters(obj, reduction = "pca", resolution = parameter$cluster_resolution)

} else {

obj <- RunHarmony(obj, group.by.vars = "orig.ident", project.dim = FALSE, assay.use = DefaultAssay(obj))

obj <- DoDimReduc(obj, reduction = "harmony", reduction.surfix = "harmony")

obj <- DoFindClusters(obj, reduction = "harmony", resolution = parameter$cluster_resolution)

}

}

}

## Draw t-SNE plot

message( "==>Draw t-SNE plot<==" )

PlotCluster(obj, reduction = 'umap', outpref = "UMAP" )

PlotCluster(obj, reduction = 'tsne', outpref = "tSNE" )

if ( ! is.null(parameter$Groups) ) {

PlotCluster(obj, reduction = 'umap', outpref = "UMAP.groups", split.by = "Groups", p1.group.by = "Groups" )

PlotCluster(obj, reduction = 'tsne', outpref = "tSNE.groups", split.by = "Groups", p1.group.by = "Groups" )

}

### Save data object

message( "==>Output obj.Rda<==" )

DefaultAssay(obj) <- "RNA"

save(obj, file = "obj.Rda")

### stat table

message( "==>Stat table<==" )

StatCluster(obj)

if ( ! is.null(parameter$Groups) ) {

StatCluster(obj, "Groups")

}

CalAvgExp(obj)

CalAvgExp(obj, group.by = "orig.ident", outfile = "AllGene.avg_exp.Samples.xls")

CalPctExp(obj, outfile = "AllGene.avg_pct.xls")

CalPctExp(obj, group.by = "orig.ident", outfile = "AllGene.avg_pct.Samples.xls")

ListCellCluster(obj)

PlotPresetMarker(obj)

### Find maker genes

message( "==>Find maker genes<==" )

obj.markers <- DoFindAllMarkers(obj, parameter)

message( "==>Output markers.Rda<==" )

save( obj.markers, file = "markers.Rda" )

#obj.markers$gene <- ChangeOUTName(obj.markers$gene, object@misc$fdata)

## stat marker

message( "==>stat marker<==" )

StatMarker(obj.markers, color = obj@misc$color.cluster)

ListMarker(obj, obj.markers)

### Top marker

message( "==>display top markers<==" )

top <- FindTopMarker(obj.markers, top_num = parameter$heatmap$top, object = obj)

PlotAboutFeatures(obj, features = unique(top$gene), outpref = "Top")

dir.create("DensityPlot/", showWarnings = F, recursive = T)

unlink("DensityPlot/*", recursive = T)

PlotDensityPlot(obj, unique(top$gene), reduction = 'umap', outpref = "DensityPlot/")

dir.create("ExpPlot/", showWarnings = F, recursive = T)

unlink("ExpPlot/*", recursive = T)

PlotFeaturePlot(obj, unique(top$gene), reduction = 'umap', outpref = "ExpPlot/ExpPlot", is.combine = FALSE)

dir.create("ViolinPlot/", showWarnings = F, recursive = T)

unlink("ViolinPlot/*", recursive = T)

PlotVlnPlot(obj, unique(top$gene), outpref = "ViolinPlot/ViolinPlot")

### Hasta la vista, baby

message( "==>All Done!<==" )

Seurat_lib.R

warning(' You may also need to load below packages : \n "Seurat", "dplyr", "ggplot2", "patchwork"', call. = FALSE)

.FilterCells <-

function (object, standard = NULL, set.num = "none", set.num.seed = 42,

filter.cells = NULL, record_file = "filtered_used_parameter.yaml")

{

if (class(object) == "Seurat") {

metadata <- object@meta.data

metadata$cell <- rownames(metadata)

}

else {

metadata <- object

metadata$orig.ident <- metadata$Sample

}

cells.use <- metadata$cell

if (!is.null(set.num) && set.num != "none") {

if (set.num == "min") {

cell_num <- min(table(metadata$orig.ident))

}

else if (set.num != "none") {

cell_num <- min(as.integer(set.num), max(table(metadata$orig.ident)))

}

seed <- set.num.seed

cells.use <- as.character(unlist(by(cells.use, metadata$orig.ident,

function(x) sample(x, min(cell_num, length(x))))))

}

pm.used <- list()

for (i in names(standard)) {

if (exists(i, metadata)) {

if (length(standard[[i]]) == 1) {

value <- standard[[i]]

if (value == "auto") {

standard[[i]] <- autothres(data = metadata[[i]],

name = i, bin = 100)

print(standard[[i]])

}

else {

cells.use <- metadata %>% filter(.data[[i]] ==

value & cell %in% cells.use) %>% select(cell) %>%

unlist()

}

}

if (length(standard[[i]]) == 2) {

lower <- standard[[i]][[1]]

upper <- standard[[i]][[2]]

cells.use <- metadata %>% filter(.data[[i]] >=

lower & .data[[i]] <= upper & cell %in% cells.use) %>%

select(cell) %>% unlist()

}

pm.used[[i]] <- standard[[i]]

}

}

if (!is.null(filter.cells)) {

cells.use <- setdiff(cells.use, filter.cells)

pm.used[["select.out.cells"]] <- filter.cells

}

if (!is.null(record_file)) {

yaml::write_yaml(pm.used, file = record_file)

}

return(cells.use)

}

.GetMetaData <-

function (object, cols = NULL)

{

name <- names(cols)

name[is.na(name) | name == ""] <- cols[is.na(name) | name ==

""]

if (is.null(name))

name <- cols

names(cols) <- name

cols <- cols[cols %in% colnames(object@meta.data)]

metadata <- object@meta.data[, cols]

colnames(metadata) <- names(cols)

metadata <- cbind(Cells = rownames(metadata), metadata)

return(metadata)

}

.my_now_dir <-

"src/Rlib"

.PlotCluster <-

function (object, reduction = NULL, cells = NULL, outfile = NULL,

p1.group.by = "orig.ident", p1.color = NULL, p1.label = FALSE,

p2.group.by = "seurat_clusters", p2.color = NULL, p2.label = TRUE,

plot.basic.size = 6, ...)

{

if (is.null(p1.color) && !is.null(p1.group.by)) {

p1.color <- switch(p1.group.by, Groups = object@misc[["color.group"]],

orig.ident = object@misc[["color.sample"]], seurat_clusters = object@misc[["color.cluster"]])

if (!is.null(cells)) {

p1.color <- p1.color[levels(droplevels(object@meta.data[cells,

p1.group.by]))]

}

}

if (is.null(p2.color) && !is.null(p2.group.by)) {

p2.color <- switch(p2.group.by, Groups = object@misc[["color.group"]],

orig.ident = object@misc[["color.sample"]], seurat_clusters = object@misc[["color.cluster"]])

if (!is.null(cells)) {

p2.color <- p2.color[levels(droplevels(object@meta.data[cells,

p2.group.by]))]

}

}

p1 <- DimPlot(object, reduction = reduction, cells = cells,

group.by = p1.group.by, cols = p1.color, label = p1.label,

...)

p2 <- DimPlot(object, reduction = reduction, cells = cells,

group.by = p2.group.by, cols = p2.color, label = p2.label,

...)

p1 <- p1 + dot_theme_default() + ggtitle(NULL)

p2 <- p2 + dot_theme_default() + ggtitle(NULL)

if (is.null(p2.group.by)) {

p <- p1

width <- plot.basic.size * 1.2

height <- plot.basic.size

}

else if (is.null(p1.group.by)) {

p <- p2

width <- plot.basic.size * 1.2

height <- plot.basic.size

}

else {

p <- p1 + p2

width <- plot.basic.size * 1.2 * 2

height <- plot.basic.size

}

if (is.null(outfile)) {

return(p)

}

else {

ggsave(p, file = outfile, width = width, height = height,

limitsize = FALSE)

}

}

.PlotClusterStat <-

function (object, stat.what = "seurat_clusters", group.by = "orig.ident",

color.st = NULL, color.gb = NULL, outpref = NULL, ...)

{

if (class(object) == "Seurat") {

metadata <- object@meta.data

}

else {

metadata <- object

}

if (is.null(color.st)) {

if ("misc" %in% slotNames(object) && exists(stat.what,

object@misc)) {

color.st <- object@misc[[stat.what]]

}

else {

color.st <- switch(stat.what, seurat_clusters = object@misc$color.cluster,

orig.ident = object@misc$color.sample, Groups = object@misc$color.group)

}

}

if (is.null(color.gb)) {

if ("misc" %in% slotNames(object) && exists(group.by,

object@misc)) {

color.gb <- object@misc[[group.by]]

}

else {

color.gb <- switch(group.by, seurat_clusters = object@misc$color.cluster,

orig.ident = object@misc$color.sample, Groups = object@misc$color.group)

}

}

name.st <- switch(stat.what, seurat_clusters = "Cluster",

orig.ident = "Samples", stat.what)

name.gb <- switch(group.by, seurat_clusters = "Cluster",

orig.ident = "Samples", group.by)

stat.what <- as.name(stat.what)

group.by <- as.name(group.by)

stat_sample <- metadata %>% group_by(`:=`(!!name.gb, !!group.by),

`:=`(!!name.st, !!stat.what)) %>% summarise(`Number of cells` = n())

p <- list()

p[["by"]] <- ggplot(stat_sample, aes_(x = as.name(name.gb),

y = ~`Number of cells`, fill = as.name(name.st)))

p[["in"]] <- ggplot(stat_sample, aes_(x = as.name(name.st),

y = ~`Number of cells`, fill = as.name(name.gb)))

if (!is.null(color.st))

p[["by"]] <- p[["by"]] + scale_fill_manual(values = color.st)

if (!is.null(color.gb))

p[["in"]] <- p[["in"]] + scale_fill_manual(values = color.gb)

geom_stack <- geom_bar(stat = "identity", position = "stack")

geom_fill <- geom_bar(stat = "identity", position = "fill")

if (is.null(outpref)) {

outpref <- paste0(name.st, ".stat")

}

for (i in names(p)) {

p[[i]] <- p[[i]] + bar_theme_default()

ggsave(p[[i]] + geom_stack, file = paste0(outpref, ".",

i, name.gb, ".pdf"), height = 6, width = 8)

ggsave(p[[i]] + geom_fill + ylab("Fraction of Cells"),

file = paste0(outpref, ".", i, name.gb, ".pct.pdf"),

height = 6, width = 8)

}

}

.PlotDensityPlot <-

function (object, features = NULL, reduction = "umap", is.return = FALSE,

outpref = NULL, is.consider.exp = FALSE, is.filter.noexp = TRUE)

{

dt <- as.data.frame(object[[reduction]]@cell.embeddings)

if (is.null(features)) {

if (nrow(dt) < 2)

return(NULL)

dt$density <- KDE(x = dt[[1]], y = dt[[2]])

outname <- paste0(outpref, "DensityPlot.pdf")

}

else {

features <- features[1]

exp <- t(as.data.frame(GetAssayData(object)[features,

, drop = F]))

dt <- cbind(dt, exp)

dt.filter <- dt[dt[[3]] > 0, , drop = FALSE]

if (nrow(dt.filter) < 2)

return(NULL)

if (is.consider.exp) {

dt.filter$density <- KDE(x = dt.filter[[1]], y = dt.filter[[2]],

z = dt.filter[[3]])

}

else {

dt.filter$density <- KDE(x = dt.filter[[1]], y = dt.filter[[2]])

}

dt <- full_join(dt, dt.filter)

dt <- dt[order(-dt$density, na.last = F), , drop = FALSE]

name <- FindFeaturesName(object, features)

name <- gsub("[ /\\]", "_", name)

name <- gsub("%20", "_", name)

outname <- paste0(outpref, "DensityPlot.", name, ".pdf")

}

p <- ggplot(dt, aes_string(x = colnames(dt)[1], y = colnames(dt)[2],

color = "density")) + geom_point() + scale_color_viridis_c(option = "A",

na.value = "grey90")

if (!is.null(features))

p <- p + ggtitle(name) + theme(plot.title = element_text(hjust = 0.5))

p <- p + dot_theme_default()

if (is.return) {

return(list(p, dt))

}

else {

ggsave(p, file = outname, width = 8, height = 7)

}

}

.PlotFeaturePlot <-

function (object, features, outfile = NULL, reduction = NULL,

is.use.name = TRUE, color.high = "blue", color.low = "lightgrey",

show.cluster.label = FALSE, nCol = NULL, plot.basic.size = 4,

group.by = "seurat_clusters", is.combine = TRUE, cols = NULL,

...)

{

if (show.cluster.label)

Idents(object) <- group.by

if (is.null(cols))

cols <- c(color.low, color.high)

plots <- FeaturePlot(object, features = features, order = TRUE,

reduction = reduction, combine = FALSE, label = show.cluster.label,

cols = cols, ...)

if (is.use.name) {

name <- FindFeaturesName(object, features)

for (i in seq(plots)) {

plots[[i]] <- plots[[i]] + ggtitle(name[i])

}

names(plots) <- name

}

else {

names(plots) <- features

}

if (is.null(nCol))

nCol <- ceiling(sqrt(length(features)))

nRow <- ceiling(length(features)/nCol)

p <- wrap_plots(plots, ncol = nCol) & dot_theme_default()

if (is.null(outfile)) {

return(p)

}

else {

ggsave(p, file = outfile, width = plot.basic.size * (6/5) *

nCol, height = plot.basic.size * nRow, limitsize = FALSE)

}

}

.Read10X <-

function (data.path, use.names = FALSE, assay = NULL)

{

data <- if (dir.exists(data.path)) {

Seurat::Read10X(data.path, gene.column = ifelse(use.names,

2, 1))

}

else if (grepl("\\.h5", data.path)) {

Seurat::Read10X_h5(data.path, use.names = use.names)

}

else {

sep <- if (grepl("\\.csv", data.path))

","

else "\t"

read.table(data.path, header = T, row.names = 1, sep = sep)

}

if (class(data) == "list") {

names(data) <- sapply(names(data), function(x) switch(x,

`Gene Expression` = "RNA", Peaks = "ATAC", x))

if (!is.null(assay)) {

data <- data[assay]

}

if (length(data) == 1) {

data <- data[[1]]

}

}

else {

if (all(grepl(pattern = "-[0-9]+$", x = colnames(data)))) {

colnames(data) <- as.vector(x = as.character(x = sapply(X = colnames(data),

FUN = Seurat:::ExtractField, field = 1, delim = "-")))

}

}

return(data)

}

.StatCluster <-

function (object, outpref = "Cluster.stat", stat.what = "seurat_clusters",

assay = DefaultAssay(object))

{

metadata <- if (class(object) == "Seurat")

object@meta.data

else object

name <- switch(stat.what, seurat_clusters = "Cluster", orig.ident = "Samples",

stat.what)

nFeature <- paste0("nFeature_", assay)

nCount <- paste0("nCount_", assay)

Cluster.stat <- metadata %>% group_by(`:=`(!!name, !!as.name(stat.what))) %>%

summarise(`Cells number` = n(), `Median Features per Cell` = median(!!as.name(nFeature)),

`Median Counts per Cell` = median(!!as.name(nCount)))

WriteTable(Cluster.stat, paste0(outpref, ".xls"))

}

.StatCluster_by <-

function (object, group.by = "orig.ident", outpref = "Cluster.stat",

stat.what = "seurat_clusters")

{

metadata <- if (class(object) == "Seurat")

object@meta.data

else object

name.stat.what <- switch(stat.what, seurat_clusters = "Cluster",

orig.ident = "Samples", stat.what)

Cluster.stat <- metadata %>% group_by(name = !!as.name(group.by),

`:=`(!!name.stat.what, !!as.name(stat.what))) %>% summarise(y = n()) %>%

mutate(`:=`(!!name.stat.what, factor(!!as.name(name.stat.what),

levels = c("Total", levels(!!as.name(name.stat.what)))))) %>%

full_join(x = metadata %>% group_by(name = !!as.name(group.by)) %>%

summarise(`:=`(!!name.stat.what, factor("Total",

levels = c("Total", levels(!!as.name(stat.what))))),

y = sum(n()))) %>% reshape2::dcast(as.formula(paste0(name.stat.what,

" ~ name")), fill = 0) %>% mutate_if(is.numeric, list(~paste0(.,

" (", round(./.[1] * 100, 2), "%)")))

name <- switch(group.by, orig.ident = "Samples", seurat_clusters = "Cluster",

group.by)

WriteTable(Cluster.stat, paste0(outpref, ".", name, ".xls"))

}

AddFData <-

function (object, ref_name_file = NULL, col.name = NULL)

{

if (!is.null(ref_name_file) && file.exists(ref_name_file)) {

fdata <- read.table(ref_name_file, row.names = 1, stringsAsFactors = F,

sep = "\t", quote = "\"")

if (ncol(fdata) > 3)

fdata <- fdata[, 1:3]

colnames(fdata) <- c("merge_name", "name", "type")[1:ncol(fdata)]

}

else {

fdata <- data.frame(name = rownames(object), row.names = rownames(object),

stringsAsFactors = F)

}

fdata$merge_name <- fdata$name

fdata$merge_name[fdata$merge_name == "-"] <- rownames(fdata)[fdata$merge_name ==

"-"]

index <- c(which(duplicated(fdata$merge_name, fromLast = T)),

which(duplicated(fdata$merge_name, fromLast = F)))

fdata$merge_name[index] <- paste0(fdata$merge_name[index],

" (", rownames(fdata)[index], ")")

fdata <- AddUnderscore(fdata)

if (is.null(col.name)) {

return(fdata)

}

else {

object@misc[[col.name]] <- fdata

return(object)

}

}

AddModuleScore <-

function (object, features, pool = NULL, nbin = 24, ctrl = 100,

k = FALSE, assay = NULL, name = "Cluster", seed = 1, search = FALSE,

...)

{

if (!is.null(x = seed)) {

set.seed(seed = seed)

}

assay.old <- DefaultAssay(object = object)

assay <- assay %||% assay.old

DefaultAssay(object = object) <- assay

assay.data <- GetAssayData(object = object)

features.old <- features

if (k) {

.NotYetUsed(arg = "k")

features <- list()

for (i in as.numeric(x = names(x = table(object@kmeans.obj[[1]]$cluster)))) {

features[[i]] <- names(x = which(x = object@kmeans.obj[[1]]$cluster ==

i))

}

cluster.length <- length(x = features)

}

else {

if (is.null(x = features)) {

stop("Missing input feature list")

}

features <- lapply(X = features, FUN = function(x) {

missing.features <- setdiff(x = x, y = rownames(x = object))

if (length(x = missing.features) > 0) {

warning("The following features are not present in the object: ",

paste(missing.features, collapse = ", "), ifelse(test = search,

yes = ", attempting to find updated synonyms",

no = ", not searching for symbol synonyms"),

call. = FALSE, immediate. = TRUE)

if (search) {

tryCatch(expr = {

updated.features <- UpdateSymbolList(symbols = missing.features,

...)

names(x = updated.features) <- missing.features

for (miss in names(x = updated.features)) {

index <- which(x == miss)

x[index] <- updated.features[miss]

}

}, error = function(...) {

warning("Could not reach HGNC's gene names database",

call. = FALSE, immediate. = TRUE)

})

missing.features <- setdiff(x = x, y = rownames(x = object))

if (length(x = missing.features) > 0) {

warning("The following features are still not present in the object: ",

paste(missing.features, collapse = ", "),

call. = FALSE, immediate. = TRUE)

}

}

}

return(intersect(x = x, y = rownames(x = object)))

})

cluster.length <- length(x = features)

}

if (!all(LengthCheck(values = features))) {

warning(paste("Could not find enough features in the object from the following feature lists:",

paste(names(x = which(x = !LengthCheck(values = features)))),

"Attempting to match case..."))

features <- lapply(X = features.old, FUN = CaseMatch,

match = rownames(x = object))

}

if (!all(LengthCheck(values = features))) {

stop(paste("The following feature lists do not have enough features present in the object:",

paste(names(x = which(x = !LengthCheck(values = features)))),

"exiting..."))

}

pool <- pool %||% rownames(x = object)

data.avg <- Matrix::rowMeans(x = assay.data[pool, , drop = F])

data.avg <- data.avg[order(data.avg)]

data.cut <- as.numeric(x = Hmisc::cut2(x = data.avg, m = round(x = length(x = data.avg)/(nbin +

1))))

ctrl <- min(ctrl, min(table(data.cut)))

names(x = data.cut) <- names(x = data.avg)

ctrl.use <- vector(mode = "list", length = cluster.length)

for (i in 1:cluster.length) {

features.use <- features[[i]]

for (j in 1:length(x = features.use)) {

ctrl.use[[i]] <- c(ctrl.use[[i]], names(x = sample(x = data.cut[which(x = data.cut ==

data.cut[features.use[j]])], size = ctrl, replace = FALSE)))

}

}

ctrl.use <- lapply(X = ctrl.use, FUN = unique)

ctrl.scores <- matrix(data = numeric(length = 1L), nrow = length(x = ctrl.use),

ncol = ncol(x = object))

for (i in 1:length(ctrl.use)) {

features.use <- ctrl.use[[i]]

ctrl.scores[i, ] <- Matrix::colMeans(x = assay.data[features.use,

, drop = F])

}

features.scores <- matrix(data = numeric(length = 1L), nrow = cluster.length,

ncol = ncol(x = object))

for (i in 1:cluster.length) {

features.use <- features[[i]]

data.use <- assay.data[features.use, , drop = FALSE]

features.scores[i, ] <- Matrix::colMeans(x = data.use)

}

features.scores.use <- features.scores - ctrl.scores

rownames(x = features.scores.use) <- paste0(name, 1:cluster.length)

features.scores.use <- as.data.frame(x = t(x = features.scores.use))

rownames(x = features.scores.use) <- colnames(x = object)

object[[colnames(x = features.scores.use)]] <- features.scores.use

CheckGC()

DefaultAssay(object = object) <- assay.old

return(object)

}

AddUnderscore <-

function (data)

{

if (!is.null(data)) {

if (!exists("underscore", data) || !exists("dash", data)) {

data$underscore <- rownames(data)

data$dash <- gsub("_", "-", rownames(data))

}

}

return(data)

}

arial_ttf <-

"src/Rlib/../../fonts/tff/msttcore/arial.ttf"

autothres <-

function (data, name = "default", bin = 100, digits = 2)

{

thres <- Otsu(data = data, bin = bin)

if (grepl("nCount", name)) {

up <- signif(thres[2], digits)

down <- -Inf

}

else if (grepl("nFeature", name)) {

up <- signif(thres[2], digits)

down <- signif(thres[1], digits)

if (up > 200) {

down <- max(down, 200)

}

}

else if (name == "percent.mito") {

if (all(data <= 1))

up <- if (thres[2] <= 0.10000000000000001)

0.10000000000000001

else if (thres[2] <= 0.25)

0.25

else signif(thres[2], digits)

else up <- if (thres[2] <= 10)

10

else if (thres[2] <= 25)

25

else signif(thres[2], digits)

down <- -Inf

}

else {

up <- signif(thres[2], digits)

down <- signif(thres[1], digits)

}

return(c(down, up))

}

bar_theme_default <-

function (font_use = "Arial")

{

library(ggplot2)

options(scipen = -1)

mytheme <- theme_bw() + theme(panel.grid = element_blank(),

panel.border = element_rect(color = "#000000", size = 0.80000000000000004),

axis.text = element_text(color = "#000000", size = 11),

axis.text.x = element_text(angle = 45, hjust = 1, vjust = 1),

axis.text.y = element_text(hjust = 1, vjust = 0.5), axis.title = element_text(color = "#000000",

size = 14, face = "plain"), axis.title.x = element_text(margin = margin(2.5,

0, 2.5, 0, "mm")), axis.title.y = element_text(margin = margin(0,

2.5, 0, 2.5, "mm")), axis.ticks = element_line(color = "#000000",

size = 0.5), axis.ticks.length = unit(0.10000000000000001,

"cm"), legend.title = element_blank(), plot.title = element_text(size = 16,

face = "plain", hjust = 0.5), plot.margin = unit(c(5,

5, 5, 5), "mm"))

if (font_use != "" & "extrafont" %in% installed.packages()) {

library(extrafont)

library(extrafontdb)

library(Rttf2pt1)

if (font_use %in% fonts()) {

mytheme <- mytheme + theme(text = element_text(family = font_use))

}

}

if (FALSE & font_use == "Arial" & "Cairo" %in% installed.packages()) {

library(Cairo)

CairoFonts(regular = "Arial:style=Regular", bold = "Arial:style=Bold",

italic = "Arial:style=Italic", bolditalic = "Arial:style=Bold Italic,BoldItalic")

source(ggsave_R)

}

if (FALSE & "showtext" %in% installed.packages()) {

library(showtext)

showtext_auto(enable = TRUE)

font_add("Arial", regular = arial_ttf)

mytheme <- mytheme + theme(text = element_text(family = "Arial"))

}

mytheme

}

bin_stat <-

function (data, bin = 256)

{

data <- as.numeric(data)

b <- floor((data - min(data))/diff(range(data)) * bin)

b[b == bin] <- bin - 1

names(b) <- data

return(b)

}

box_theme_default <-

function (font_use = "Arial")

{

library(ggplot2)

mytheme <- theme_bw() + theme(panel.grid.major = element_blank(),

panel.grid.minor = element_blank(), panel.border = element_rect(color = "#000000",

size = 0.80000000000000004), axis.text = element_text(color = "#000000",

size = 11), axis.text.x = element_text(angle = 0,

hjust = 0.5, vjust = 0.5), axis.text.y = element_text(hjust = 0.5,

vjust = 0.5), axis.title = element_text(color = "#000000",

size = 14, face = "plain"), axis.title.x = element_text(margin = margin(2.5,

0, 2.5, 0, "mm")), axis.title.y = element_text(margin = margin(0,

2.5, 0, 2.5, "mm")), axis.ticks = element_line(color = "#000000",

size = 0.5), axis.ticks.length = unit(0.11, "cm"),

legend.title = element_blank(), legend.justification = "center",

plot.title = element_text(size = 16, face = "plain",

hjust = 0.5), plot.margin = unit(c(5, 5, 5, 5), "mm"))

if (font_use != "" & "extrafont" %in% installed.packages()) {

library(extrafont)

library(extrafontdb)

library(Rttf2pt1)

if (font_use %in% fonts()) {

mytheme <- mytheme + theme(text = element_text(family = font_use))

}

}

if (FALSE & font_use == "Arial" & "Cairo" %in% installed.packages()) {

library(Cairo)

CairoFonts(regular = "Arial:style=Regular", bold = "Arial:style=Bold",

italic = "Arial:style=Italic", bolditalic = "Arial:style=Bold Italic,BoldItalic")

source(ggsave_R)

}

if (FALSE & "showtext" %in% installed.packages()) {

library(showtext)

showtext_auto(enable = TRUE)

font_add("Arial", regular = arial_ttf)

mytheme <- mytheme + theme(text = element_text(family = "Arial"))

}

mytheme

}

box_theme_splitviolin <-

function (font_use = "Arial")

{

library(ggplot2)

mytheme <- theme_bw() + theme(panel.grid.major = element_blank(),

panel.grid.minor = element_blank(), panel.border = element_rect(color = "#000000",

size = 0.80000000000000004), axis.text = element_text(color = "#000000",

size = 11), axis.text.x = element_text(angle = 0,

hjust = 0.5, vjust = 0.5), axis.text.y = element_text(hjust = 0.5,

vjust = 0.5), axis.title = element_text(color = "#000000",

size = 14, face = "plain"), axis.title.x = element_text(margin = margin(2.5,

0, 2.5, 0, "mm")), axis.title.y = element_text(margin = margin(0,

2.5, 0, 2.5, "mm")), axis.ticks = element_line(color = "#000000",

size = 0.5), axis.ticks.length = unit(0.11, "cm"),

legend.title = element_blank(), legend.justification = "center",

plot.title = element_text(size = 16, face = "plain",

hjust = 0.5), plot.margin = unit(c(5, 5, 5, 5), "mm"))

if (font_use != "" & "extrafont" %in% installed.packages()) {

library(extrafont)

library(extrafontdb)

library(Rttf2pt1)

if (font_use %in% fonts()) {

mytheme <- mytheme + theme(text = element_text(family = font_use))

}

}

if (FALSE & font_use == "Arial" & "Cairo" %in% installed.packages()) {

library(Cairo)

CairoFonts(regular = "Arial:style=Regular", bold = "Arial:style=Bold",

italic = "Arial:style=Italic", bolditalic = "Arial:style=Bold Italic,BoldItalic")

source(ggsave_R)

}

if (FALSE & "showtext" %in% installed.packages()) {

library(showtext)

showtext_auto(enable = TRUE)

}

mytheme

}

CalAvgExp <-

function (object, features = NULL, group.by = NULL, assay = NULL,

slot = "data", is.expm1 = ifelse(slot == "data", TRUE, FALSE),

is.return = FALSE, is.reverse = FALSE, is.bulk = FALSE, outfile = "AllGene.avg_exp.xls",

preftext = "Cluster")

{

data <- GetAssayData(object, assay = assay, slot = slot)

if (!is.null(features))

data <- data[features, , drop = FALSE]

if (is.expm1)

data <- expm1(data)

if (!is.null(group.by))

Idents(object) <- group.by

mean_exp <- do.call(cbind, by(colnames(object), Idents(object),

function(x) {

y <- if (is.reverse) {

setdiff(colnames(object), x)

}

else {

x

}

Matrix::rowMeans(data[, y, drop = F])

}, simplify = FALSE))

if (is.bulk) {

mean_exp <- cbind(bulk = Matrix::rowMeans(data), mean_exp)

}

if (is.return) {

return(mean_exp)

}

else {

if (!is.null(preftext)) {

colnames(mean_exp) <- paste(preftext, colnames(mean_exp))

}

Gene_ID <- ChangeOUTName(rownames(mean_exp), object@misc$fdata)

Gene_name <- FindFeaturesName(object, rownames(mean_exp),

"name")

mean_exp <- cbind(Gene_ID = Gene_ID, Gene_name = Gene_name,

mean_exp)

WriteTable(mean_exp, file = outfile)

}

}

CalPctExp <-

function (object, features = NULL, group.by = NULL, assay = NULL,

slot = "counts", is.return = FALSE, is.reverse = FALSE, is.bulk = FALSE,

outfile = "AllGene.avg_pct.xls")

{

data <- GetAssayData(object, assay = assay, slot = slot)

if (!is.null(features))

data <- data[features, , drop = FALSE]

if (!is.null(group.by))

Idents(object) <- group.by

mean_exp <- do.call(cbind, by(colnames(object), Idents(object),

function(x) {

y <- if (is.reverse) {

setdiff(colnames(object), x)

}

else {

x

}

Matrix::rowSums(data[, y, drop = F] > 0)/length(y)

}))

if (is.bulk) {

mean_exp <- cbind(bulk = Matrix::rowSums(data > 0)/ncol(data),

mean_exp)

}

if (is.return) {

return(mean_exp)

}

else {

colnames(mean_exp) <- paste("Cluster", colnames(mean_exp))

Gene_ID <- ChangeOUTName(rownames(mean_exp), object@misc$fdata)

Gene_name <- FindFeaturesName(object, rownames(mean_exp),

"name")

mean_exp <- cbind(Gene_ID = Gene_ID, Gene_name = Gene_name,

mean_exp)

WriteTable(mean_exp, file = outfile)

}

}

ChangeOUTName <-

function (features, fdata)

{

features <- as.character(features)

fdata <- AddUnderscore(fdata)

if (!is.null(fdata) && all(features %in% fdata$dash)) {

underscore_id <- fdata$underscore

names(underscore_id) <- fdata$dash

features <- underscore_id[features]

}

return(features)

}

CheckPCA <-

function (object, reduction = "pca")

{

p1 <- DimPlot(object, reduction = reduction, group.by = "orig.ident")

w <- 6

if (exists("Phase", object@meta.data)) {

p2 <- DimPlot(object, reduction = reduction, group.by = "Phase")

p1 <- p1 + p2

w <- 12

}

ggsave(p1, file = "pcaPlot.pdf", width = w, height = 6)

dims <- min(20, ncol(Reductions(object, reduction)))

p3 <- DimHeatmap(object, dims = seq(dims), cells = 500, balanced = TRUE,

ncol = 4, fast = FALSE, reduction = reduction)

ggsave(p3, file = "pcaHeatmap.pdf", width = min(4, dims) *

4, height = ceiling(dims/4) * 4)

if (!is.null(dev.list()))

dev.off()

p4 <- ElbowPlot(object, ndims = ncol(Reductions(object, reduction)),

reduction = reduction)

ggsave(p4, file = "pcaElbowPlot.pdf", width = 6, height = 6)

}

CheckVariableFeature <-

function (object)

{

top10 <- head(VariableFeatures(object), 10)

plot1 <- VariableFeaturePlot(object)

plot2 <- LabelPoints(plot = plot1, points = top10, labels = object@misc$fdata[top10,

"merge_name"], repel = TRUE, xnudge = 0, ynudge = 0)

plot2 <- plot2 + theme(legend.position = "top")

ggsave(plot2, file = "Variable_gene.pdf", width = 6, height = 6)

writeLines(VariableFeatures(object), "var_gene.xls")

}

col2grey <-

function (red, green, blue, algorithms = c("luminance", "luma",

"average", "desaturation", "max", "min", "red", "blue", "green"),

maxColorValue = 255)

{

if (missing(green) && missing(blue)) {

if (is.matrix(red) || is.data.frame(red)) {

red <- data.matrix(red)

if (ncol(red) < 3L)

stop("at least 3 columns needed")

}

else {

red <- t(col2rgb(red))

}

green <- red[, 2L]

blue <- red[, 3L]

red <- red[, 1L]

}

algorithms <- match.arg(algorithms)

Y <- switch(algorithms, luminance = 0.29899999999999999 *

red + 0.58699999999999997 * green + 0.114 * blue, luma = 0.21260000000000001 *

red + 0.71519999999999995 * green + 0.0722 * blue, average = mean(c(red,

green, blue)), desaturation = (max(red, green, blue) +

min(red, green, blue))/2, max = max(red, green, blue),

min = min(red, green, blue), red = red, green = green,

blue = blue)

color <- rgb(red = Y, green = Y, blue = Y, maxColorValue = maxColorValue)

return(color)

}

color.list <-

list(venn = list(default = list(`2` = c("#FFBDC0", "#C7D4EE"),

`3` = c("#69A4F9", "#FFCC66", "#FCB4B4"), `4` = c("#FFCC66",

"#BFE046", "#FCB4B4", "#69A4F9"), `5` = c("#BFE046", "#69A4F9",

"#ACB9EA", "#FCB4B4", "#FFCC66"), `6` = c("#FCB4B4", "#FFCC66",

"#BFE046", "#28C580", "#69A4F9", "#ACB9EA"), `7` = c("#FCB4B4",

"#FFCC66", "#BFE046", "#28C580", "#69A4F9", "#ACB9EA", "#C3C3C3"

), `0` = c("#FCB4B4", "#FFCC66", "#BFE046", "#28C580", "#69A4F9",

"#ACB9EA", "#C3C3C3"))), line = list(default = list(`1` = "#2771A7",

`2` = c("#D32421", "#2771A7"), `3` = c("#3A9736", "#2771A7",

"#D32421"), `4` = c("#3A9736", "#2771A7", "#C6AFD1", "#D32421"

), `5` = c("#3A9736", "#2771A7", "#C6AFD1", "#D32421", "#F3BB6F"

), `8` = c("#D32421", "#F09594", "#2771A7", "#3A9736", "#F3BB6F",

"#C6AFD1", "#831D20", "#A2C8DC"), `10` = c("#A2C8DC", "#F09594",

"#2771A7", "#C6AFD1", "#D32421", "#831D20", "#3A9736", "#F3BB6F",

"#A3A49E", "#5B4232"), `15` = c("#00468B", "#925E9F", "#759EDD",

"#0099B4", "#0A7C2E", "#B8D24D", "#EDE447", "#FAB158", "#FF7777",

"#FD0000", "#AD002A", "#AE8691", "#DEB8A1", "#CE9573", "#5B4232"

), `20` = c("#00468B", "#5377A7", "#6C6DA4", "#925E9F", "#759EDD",

"#0099B4", "#42C1BB", "#76D1B1", "#0A7C2E", "#B8D24D", "#EDE447",

"#FAB158", "#FDAF91", "#FF7777", "#FD0000", "#AD002A", "#AE8691",

"#DEB8A1", "#4C4E4E", "#5B4232"), `30` = c("#00468B", "#5377A7",

"#3B81AB", "#5C298F", "#6C6DA4", "#925E9F", "#759EDD", "#76C8DC",

"#0099B4", "#42C1BB", "#76D1B1", "#0F8074", "#0A7C2E", "#28AA6C",

"#B8D24D", "#EDE447", "#FAB158", "#FDAF91", "#E67E74", "#FF7777",

"#FD0000", "#AD002A", "#792244", "#AD556B", "#AE8691", "#CE9573",

"#B09F91", "#DEB8A1", "#4C4E4E", "#5B4232"), `0` = c("#00468B",

"#5377A7", "#6C6DA4", "#925E9F", "#759EDD", "#0099B4", "#42C1BB",

"#76D1B1", "#0A7C2E", "#B8D24D", "#EDE447", "#FAB158", "#FDAF91",

"#FF7777", "#FD0000", "#AD002A", "#AE8691", "#DEB8A1", "#4C4E4E",

"#5B4232"))), pie = list(default = list(`1` = "#42B540",

`2` = c("#42B540", "#EDE447"), `3` = c("#42B540", "#EDE447",

"#FF7777"), `4` = c("#00468B", "#42B540", "#EDE447", "#FF7777"

), `5` = c("#00468B", "#42B540", "#EDE447", "#759EDD", "#FF7777"

), `8` = c("#00468B", "#0099B4", "#76D1B1", "#42B540", "#EDE447",

"#FF7777", "#AD002A", "#759EDD"), `10` = c("#00468B", "#0099B4",

"#76D1B1", "#42B540", "#EDE447", "#FF7777", "#AD002A", "#759EDD",

"#DEB8A1", "#5B4232"), `20` = c("#00468B", "#5377A7", "#6C6DA4",

"#925E9F", "#759EDD", "#0099B4", "#42C1BB", "#76D1B1", "#42B540",

"#B8D24D", "#EDE447", "#FAB158", "#FDAF91", "#FF7777", "#FD0000",

"#AD002A", "#AE8691", "#CE9573", "#DEB8A1", "#5B4232"), `30` = c("#00468B",

"#5377A7", "#3B81AB", "#5C298F", "#6C6DA4", "#925E9F", "#759EDD",

"#76C8DC", "#0099B4", "#42C1BB", "#76D1B1", "#0F8074", "#28AA6C",

"#42B540", "#B8D24D", "#EDE447", "#FAB158", "#FDAF91", "#E67E74",

"#FF7777", "#FD0000", "#AD002A", "#792244", "#AD556B", "#AE8691",

"#CE9573", "#B09F91", "#756455", "#DEB8A1", "#5B4232"), `0` = c("#2e0a4a",

"#7a1b6c", "#15ad68", "#ded531", "#db9421", "#14b5b5", "#ede893",

"#76cfed", "#4599de", "#db2830", "#5F64AC", "#B271AD", "#eda4d3",

"#d33c67", "#EC6925", "#a155f9", "#70F2D3", "#6FBA33", "#EDAC2E",

"#096d42", "#4ec4b5", "#a36924", "#125fb2", "#7350EB", "#891a3a",

"#bf109a", "#E8851F", "#e77def", "#4ebee5", "#69a4f9", "#f9cfa5",

"#13D9B1", "#bfe046", "#DEB8A1", "#5B4232"))), twopie = list(

default = list(`1` = "#2771A7", `2` = c("#D32421", "#2771A7"

), `3` = c("#3A9736", "#2771A7", "#D32421"), `4` = c("#3A9736",

"#2771A7", "#C6AFD1", "#D32421"), `5` = c("#3A9736", "#2771A7",

"#C6AFD1", "#D32421", "#F3BB6F"), `8` = c("#D32421", "#F09594",

"#2771A7", "#3A9736", "#F3BB6F", "#C6AFD1", "#831D20", "#A2C8DC"

), `10` = c("#A2C8DC", "#F09594", "#2771A7", "#C6AFD1", "#D32421",

"#831D20", "#3A9736", "#F3BB6F", "#A3A49E", "#5B4232"), `15` = c("#00468B",

"#925E9F", "#759EDD", "#0099B4", "#0A7C2E", "#B8D24D", "#EDE447",

"#FAB158", "#FF7777", "#FD0000", "#AD002A", "#AE8691", "#DEB8A1",

"#CE9573", "#5B4232"), `20` = c("#00468B", "#5377A7", "#6C6DA4",

"#925E9F", "#759EDD", "#0099B4", "#42C1BB", "#76D1B1", "#0A7C2E",

"#B8D24D", "#EDE447", "#FAB158", "#FDAF91", "#FF7777", "#FD0000",

"#AD002A", "#AE8691", "#DEB8A1", "#4C4E4E", "#5B4232"), `30` = c("#00468B",

"#5377A7", "#3B81AB", "#5C298F", "#6C6DA4", "#925E9F", "#759EDD",

"#76C8DC", "#0099B4", "#42C1BB", "#76D1B1", "#0F8074", "#0A7C2E",

"#28AA6C", "#B8D24D", "#EDE447", "#FAB158", "#FDAF91", "#E67E74",

"#FF7777", "#FD0000", "#AD002A", "#792244", "#AD556B", "#AE8691",

"#CE9573", "#B09F91", "#DEB8A1", "#4C4E4E", "#5B4232"), `0` = c("#00468B",

"#5377A7", "#6C6DA4", "#925E9F", "#759EDD", "#0099B4", "#42C1BB",

"#76D1B1", "#0A7C2E", "#B8D24D", "#EDE447", "#FAB158", "#FDAF91",

"#FF7777", "#FD0000", "#AD002A", "#AE8691", "#DEB8A1", "#4C4E4E",

"#5B4232"))), bar = list(default = list(`1` = "#2771A7",

`2` = c("#D32421", "#2771A7"), `3` = c("#3A9736", "#2771A7",

"#D32421"), `4` = c("#3A9736", "#2771A7", "#C6AFD1", "#D32421"

), `5` = c("#3A9736", "#2771A7", "#C6AFD1", "#D32421", "#F3BB6F"

), `8` = c("#D32421", "#F09594", "#2771A7", "#3A9736", "#F3BB6F",

"#C6AFD1", "#831D20", "#A2C8DC"), `10` = c("#A2C8DC", "#F09594",

"#2771A7", "#C6AFD1", "#D32421", "#831D20", "#3A9736", "#F3BB6F",

"#A3A49E", "#5B4232"), `15` = c("#00468B", "#925E9F", "#759EDD",

"#0099B4", "#0A7C2E", "#B8D24D", "#EDE447", "#FAB158", "#FF7777",

"#FD0000", "#AD002A", "#AE8691", "#DEB8A1", "#CE9573", "#5B4232"

), `20` = c("#00468B", "#5377A7", "#6C6DA4", "#925E9F", "#759EDD",

"#0099B4", "#42C1BB", "#76D1B1", "#0A7C2E", "#B8D24D", "#EDE447",

"#FAB158", "#FDAF91", "#FF7777", "#FD0000", "#AD002A", "#AE8691",

"#DEB8A1", "#4C4E4E", "#5B4232"), `30` = c("#00468B", "#5377A7",

"#3B81AB", "#5C298F", "#6C6DA4", "#925E9F", "#759EDD", "#76C8DC",

"#0099B4", "#42C1BB", "#76D1B1", "#0F8074", "#0A7C2E", "#28AA6C",

"#B8D24D", "#EDE447", "#FAB158", "#FDAF91", "#E67E74", "#FF7777",

"#FD0000", "#AD002A", "#792244", "#AD556B", "#AE8691", "#CE9573",

"#B09F91", "#DEB8A1", "#4C4E4E", "#5B4232"), `0` = c("#00468B",

"#5377A7", "#6C6DA4", "#925E9F", "#759EDD", "#0099B4", "#42C1BB",

"#76D1B1", "#0A7C2E", "#B8D24D", "#EDE447", "#FAB158", "#FDAF91",

"#FF7777", "#FD0000", "#AD002A", "#AE8691", "#DEB8A1", "#4C4E4E",

"#5B4232")), stack = list(`1` = "#42B540", `2` = c("#42B540",

"#EDE447"), `3` = c("#42B540", "#EDE447", "#FF7777"), `4` = c("#00468B",

"#42B540", "#EDE447", "#FF7777"), `5` = c("#00468B", "#42B540",

"#EDE447", "#759EDD", "#FF7777"), `8` = c("#00468B", "#0099B4",

"#76D1B1", "#42B540", "#EDE447", "#FF7777", "#AD002A", "#759EDD"

), `10` = c("#00468B", "#0099B4", "#76D1B1", "#42B540", "#EDE447",

"#FF7777", "#AD002A", "#759EDD", "#DEB8A1", "#5B4232"), `20` = c("#00468B",

"#5377A7", "#6C6DA4", "#925E9F", "#759EDD", "#0099B4", "#42C1BB",

"#76D1B1", "#42B540", "#B8D24D", "#EDE447", "#FAB158", "#FDAF91",

"#FF7777", "#FD0000", "#AD002A", "#AE8691", "#CE9573", "#DEB8A1",

"#5B4232"), `30` = c("#00468B", "#5377A7", "#3B81AB", "#5C298F",

"#6C6DA4", "#925E9F", "#759EDD", "#76C8DC", "#0099B4", "#42C1BB",

"#76D1B1", "#0F8074", "#28AA6C", "#42B540", "#B8D24D", "#EDE447",

"#FAB158", "#FDAF91", "#E67E74", "#FF7777", "#FD0000", "#AD002A",

"#792244", "#AD556B", "#AE8691", "#CE9573", "#B09F91", "#756455",

"#DEB8A1", "#5B4232"), `0` = c("#2e0a4a", "#7a1b6c", "#15ad68",

"#ded531", "#db9421", "#14b5b5", "#ede893", "#76cfed", "#4599de",

"#db2830", "#5F64AC", "#B271AD", "#eda4d3", "#d33c67", "#EC6925",

"#a155f9", "#70F2D3", "#6FBA33", "#EDAC2E", "#096d42", "#4ec4b5",

"#a36924", "#125fb2", "#7350EB", "#891a3a", "#bf109a", "#E8851F",

"#e77def", "#4ebee5", "#69a4f9", "#f9cfa5", "#13D9B1", "#bfe046",

"#DEB8A1", "#5B4232")), polarbar = list(`1` = c("#54778f", "#4EB043",

"#E69D2A", "#DD4714", "#A61650"), `2` = c("#440154", "#3B528B",

"#21908C", "#5DC863", "#FDE725"), `3` = c("#806E6E", "#B7BBC4",

"#D8A69F"), `0` = c("#54778f", "#4EB043", "#E69D2A", "#DD4714",

"#A61650"))), hist = list(default = list(`1` = "#2771A7", `2` = c("#D32421",

"#2771A7"), `3` = c("#3A9736", "#2771A7", "#D32421"), `4` = c("#3A9736",

"#2771A7", "#C6AFD1", "#D32421"), `5` = c("#3A9736", "#2771A7",

"#C6AFD1", "#D32421", "#F3BB6F"), `8` = c("#D32421", "#F09594",

"#2771A7", "#3A9736", "#F3BB6F", "#C6AFD1", "#831D20", "#A2C8DC"

), `10` = c("#A2C8DC", "#F09594", "#2771A7", "#C6AFD1", "#D32421",

"#831D20", "#3A9736", "#F3BB6F", "#A3A49E", "#5B4232"), `15` = c("#00468B",

"#925E9F", "#759EDD", "#0099B4", "#0A7C2E", "#B8D24D", "#EDE447",

"#FAB158", "#FF7777", "#FD0000", "#AD002A", "#AE8691", "#DEB8A1",

"#CE9573", "#5B4232"), `20` = c("#00468B", "#5377A7", "#6C6DA4",

"#925E9F", "#759EDD", "#0099B4", "#42C1BB", "#76D1B1", "#0A7C2E",

"#B8D24D", "#EDE447", "#FAB158", "#FDAF91", "#FF7777", "#FD0000",

"#AD002A", "#AE8691", "#DEB8A1", "#4C4E4E", "#5B4232"), `30` = c("#00468B",

"#5377A7", "#3B81AB", "#5C298F", "#6C6DA4", "#925E9F", "#759EDD",

"#76C8DC", "#0099B4", "#42C1BB", "#76D1B1", "#0F8074", "#0A7C2E",

"#28AA6C", "#B8D24D", "#EDE447", "#FAB158", "#FDAF91", "#E67E74",

"#FF7777", "#FD0000", "#AD002A", "#792244", "#AD556B", "#AE8691",

"#CE9573", "#B09F91", "#DEB8A1", "#4C4E4E", "#5B4232"), `0` = c("#00468B",

"#5377A7", "#6C6DA4", "#925E9F", "#759EDD", "#0099B4", "#42C1BB",

"#76D1B1", "#0A7C2E", "#B8D24D", "#EDE447", "#FAB158", "#FDAF91",

"#FF7777", "#FD0000", "#AD002A", "#AE8691", "#DEB8A1", "#4C4E4E",

"#5B4232"))), heatmap = list(default = list(`0` = c("#2F70AD",

"#FFFFFF", "#BA2831"), `1` = c("#4785B6", "#FFFFFF", "#FF1717"

), `2` = c("#5B8089", "#FFFFFF", "#009933"), `3` = c("#5B8089",

"#FDECBE", "#009933"), `4` = c("#3F99CB", "#FFFFFD", "#A1CD46"

), `5` = c("#FFFD36", "#FDFFFE", "#2C9B68"), `6` = c("#013F84",

"#FDECBE", "#02908B")), corr = list(`0` = c("#FFFFFF", "#C4DEEC",

"#2166AC"), `1` = c("#4785B6", "#FFFFFF", "#FF1717"), `2` = c("#5B8089",

"#FFFFFF", "#009933"), `3` = c("#5B8089", "#FDECBE", "#009933"

), `4` = c("#3F99CB", "#FFFFFD", "#A1CD46"), `5` = c("#FFFD36",

"#FDFFFE", "#2C9B68"), `6` = c("#013F84", "#FDECBE", "#02908B"

), `7` = c("#2F70AD", "#FFFFFF", "#BA2831"))), dot = list(default = list(

`1` = "#2771A7", `2` = c("#D32421", "#2771A7"), `3` = c("#3A9736",

"#2771A7", "#D32421"), `4` = c("#3A9736", "#2771A7", "#C6AFD1",

"#D32421"), `5` = c("#3A9736", "#2771A7", "#C6AFD1", "#D32421",

"#F3BB6F"), `8` = c("#D32421", "#F09594", "#2771A7", "#3A9736",

"#F3BB6F", "#C6AFD1", "#831D20", "#A2C8DC"), `10` = c("#A2C8DC",

"#F09594", "#2771A7", "#C6AFD1", "#D32421", "#831D20", "#3A9736",

"#F3BB6F", "#A3A49E", "#5B4232"), `15` = c("#00468B", "#925E9F",

"#759EDD", "#0099B4", "#0A7C2E", "#B8D24D", "#EDE447", "#FAB158",

"#FF7777", "#FD0000", "#AD002A", "#AE8691", "#DEB8A1", "#CE9573",

"#5B4232"), `20` = c("#00468B", "#5377A7", "#6C6DA4", "#925E9F",

"#759EDD", "#0099B4", "#42C1BB", "#76D1B1", "#0A7C2E", "#B8D24D",

"#EDE447", "#FAB158", "#FDAF91", "#FF7777", "#FD0000", "#AD002A",

"#AE8691", "#DEB8A1", "#4C4E4E", "#5B4232"), `30` = c("#00468B",

"#5377A7", "#3B81AB", "#5C298F", "#6C6DA4", "#925E9F", "#759EDD",

"#76C8DC", "#0099B4", "#42C1BB", "#76D1B1", "#0F8074", "#0A7C2E",

"#28AA6C", "#B8D24D", "#EDE447", "#FAB158", "#FDAF91", "#E67E74",

"#FF7777", "#FD0000", "#AD002A", "#792244", "#AD556B", "#AE8691",

"#CE9573", "#B09F91", "#DEB8A1", "#4C4E4E", "#5B4232"), `0` = c("#00468B",

"#5377A7", "#6C6DA4", "#925E9F", "#759EDD", "#0099B4", "#42C1BB",

"#76D1B1", "#0A7C2E", "#B8D24D", "#EDE447", "#FAB158", "#FDAF91",

"#FF7777", "#FD0000", "#AD002A", "#AE8691", "#DEB8A1", "#4C4E4E",

"#5B4232")), volcano = list(`0` = c("#F9766D", "#00000032",

"#609DFF"))), box = list(default = list(`1` = "#2771A7", `2` = c("#D32421",

"#2771A7"), `3` = c("#3A9736", "#2771A7", "#D32421"), `4` = c("#3A9736",

"#2771A7", "#C6AFD1", "#D32421"), `5` = c("#3A9736", "#2771A7",

"#C6AFD1", "#D32421", "#F3BB6F"), `8` = c("#D32421", "#F09594",

"#2771A7", "#3A9736", "#F3BB6F", "#C6AFD1", "#831D20", "#A2C8DC"

), `10` = c("#A2C8DC", "#F09594", "#2771A7", "#C6AFD1", "#D32421",

"#831D20", "#3A9736", "#F3BB6F", "#A3A49E", "#5B4232"), `15` = c("#00468B",

"#925E9F", "#759EDD", "#0099B4", "#0A7C2E", "#B8D24D", "#EDE447",

"#FAB158", "#FF7777", "#FD0000", "#AD002A", "#AE8691", "#DEB8A1",

"#CE9573", "#5B4232"), `20` = c("#00468B", "#5377A7", "#6C6DA4",

"#925E9F", "#759EDD", "#0099B4", "#42C1BB", "#76D1B1", "#0A7C2E",

"#B8D24D", "#EDE447", "#FAB158", "#FDAF91", "#FF7777", "#FD0000",

"#AD002A", "#AE8691", "#DEB8A1", "#4C4E4E", "#5B4232"), `30` = c("#00468B",

"#5377A7", "#3B81AB", "#5C298F", "#6C6DA4", "#925E9F", "#759EDD",

"#76C8DC", "#0099B4", "#42C1BB", "#76D1B1", "#0F8074", "#0A7C2E",

"#28AA6C", "#B8D24D", "#EDE447", "#FAB158", "#FDAF91", "#E67E74",

"#FF7777", "#FD0000", "#AD002A", "#792244", "#AD556B", "#AE8691",

"#CE9573", "#B09F91", "#DEB8A1", "#4C4E4E", "#5B4232"), `0` = c("#00468B",

"#5377A7", "#6C6DA4", "#925E9F", "#759EDD", "#0099B4", "#42C1BB",

"#76D1B1", "#0A7C2E", "#B8D24D", "#EDE447", "#FAB158", "#FDAF91",

"#FF7777", "#FD0000", "#AD002A", "#AE8691", "#DEB8A1", "#4C4E4E",

"#5B4232")), split = list(`0` = c("#F9766D", "#609DFF"), `1` = c("#56B4E9",

"#E69F00"))), all = list(default = list(`1` = "#2771A7", `2` = c("#D32421",

"#2771A7"), `3` = c("#3A9736", "#2771A7", "#D32421"), `4` = c("#3A9736",

"#2771A7", "#C6AFD1", "#D32421"), `5` = c("#3A9736", "#2771A7",

"#C6AFD1", "#D32421", "#F3BB6F"), `8` = c("#D32421", "#F09594",

"#2771A7", "#3A9736", "#F3BB6F", "#C6AFD1", "#831D20", "#A2C8DC"

), `10` = c("#A2C8DC", "#F09594", "#2771A7", "#C6AFD1", "#D32421",

"#831D20", "#3A9736", "#F3BB6F", "#A3A49E", "#5B4232"), `15` = c("#00468B",

"#925E9F", "#759EDD", "#0099B4", "#0A7C2E", "#B8D24D", "#EDE447",

"#FAB158", "#FF7777", "#FD0000", "#AD002A", "#AE8691", "#DEB8A1",

"#CE9573", "#5B4232"), `20` = c("#00468B", "#5377A7", "#6C6DA4",

"#925E9F", "#759EDD", "#0099B4", "#42C1BB", "#76D1B1", "#0A7C2E",

"#B8D24D", "#EDE447", "#FAB158", "#FDAF91", "#FF7777", "#FD0000",

"#AD002A", "#AE8691", "#DEB8A1", "#4C4E4E", "#5B4232"), `30` = c("#00468B",

"#5377A7", "#3B81AB", "#5C298F", "#6C6DA4", "#925E9F", "#759EDD",

"#76C8DC", "#0099B4", "#42C1BB", "#76D1B1", "#0F8074", "#0A7C2E",

"#28AA6C", "#B8D24D", "#EDE447", "#FAB158", "#FDAF91", "#E67E74",

"#FF7777", "#FD0000", "#AD002A", "#792244", "#AD556B", "#AE8691",

"#CE9573", "#B09F91", "#DEB8A1", "#4C4E4E", "#5B4232"), `0` = c("#00468B",

"#5377A7", "#6C6DA4", "#925E9F", "#759EDD", "#0099B4", "#42C1BB",

"#76D1B1", "#0A7C2E", "#B8D24D", "#EDE447", "#FAB158", "#FDAF91",

"#FF7777", "#FD0000", "#AD002A", "#AE8691", "#DEB8A1", "#4C4E4E",

"#5B4232")), dark = list(`1` = "#0A7C2E", `2` = c("#D32421",

"#00468B"), `3` = c("#0A7C2E", "#00468B", "#D32421"), `4` = c("#0A7C2E",

"#00468B", "#925E9F", "#D32421"), `5` = c("#00468B", "#0A7C2E",

"#0099B4", "#925E9F", "#AD002A"), `8` = c("#00468B", "#925E9F",

"#0099B4", "#0A7C2E", "#FDAF91", "#FD0000", "#AD002A", "#DEB8A1"

), `10` = c("#00468B", "#925E9F", "#0099B4", "#3DB88C", "#0A7C2E",

"#FDAF91", "#FD0000", "#AD002A", "#DEB8A1", "#5B4232"), `15` = c("#00468B",

"#925E9F", "#759EDD", "#0099B4", "#0A7C2E", "#B8D24D", "#EDE447",

"#FAB158", "#FF7777", "#FD0000", "#AD002A", "#AE8691", "#DEB8A1",

"#CE9573", "#5B4232"), `20` = c("#00468B", "#5377A7", "#6C6DA4",

"#925E9F", "#759EDD", "#0099B4", "#42C1BB", "#76D1B1", "#0A7C2E",

"#B8D24D", "#EDE447", "#FAB158", "#FDAF91", "#FF7777", "#FD0000",

"#AD002A", "#AE8691", "#DEB8A1", "#4C4E4E", "#5B4232"), `30` = c("#00468B",

"#5377A7", "#3B81AB", "#5C298F", "#6C6DA4", "#925E9F", "#759EDD",

"#76C8DC", "#0099B4", "#42C1BB", "#76D1B1", "#0F8074", "#0A7C2E",

"#28AA6C", "#B8D24D", "#EDE447", "#FAB158", "#FDAF91", "#E67E74",

"#FF7777", "#FD0000", "#AD002A", "#792244", "#AD556B", "#AE8691",

"#CE9573", "#B09F91", "#DEB8A1", "#4C4E4E", "#5B4232"), `0` = c("#00468B",

"#5377A7", "#6C6DA4", "#925E9F", "#759EDD", "#0099B4", "#42C1BB",

"#76D1B1", "#0A7C2E", "#B8D24D", "#EDE447", "#FAB158", "#FDAF91",

"#FF7777", "#FD0000", "#AD002A", "#AE8691", "#DEB8A1", "#4C4E4E",

"#5B4232")), light = list(`1` = "#2771A7", `2` = c("#D32421",

"#2771A7"), `3` = c("#3A9736", "#2771A7", "#D32421"), `4` = c("#3A9736",

"#2771A7", "#C6AFD1", "#D32421"), `5` = c("#3A9736", "#2771A7",

"#C6AFD1", "#D32421", "#F3BB6F"), `8` = c("#D32421", "#F09594",

"#2771A7", "#3A9736", "#F3BB6F", "#C6AFD1", "#831D20", "#A2C8DC"

), `10` = c("#A2C8DC", "#F09594", "#2771A7", "#C6AFD1", "#D32421",

"#831D20", "#3A9736", "#F3BB6F", "#A3A49E", "#5B4232"), `15` = c("#00468B",

"#925E9F", "#759EDD", "#0099B4", "#0A7C2E", "#B8D24D", "#EDE447",

"#FAB158", "#FF7777", "#FD0000", "#AD002A", "#AE8691", "#DEB8A1",

"#CE9573", "#5B4232"), `20` = c("#00468B", "#5377A7", "#6C6DA4",

"#925E9F", "#759EDD", "#0099B4", "#42C1BB", "#76D1B1", "#0A7C2E",

"#B8D24D", "#EDE447", "#FAB158", "#FDAF91", "#FF7777", "#FD0000",

"#AD002A", "#AE8691", "#DEB8A1", "#4C4E4E", "#5B4232"), `30` = c("#00468B",

"#5377A7", "#3B81AB", "#5C298F", "#6C6DA4", "#925E9F", "#759EDD",

"#76C8DC", "#0099B4", "#42C1BB", "#76D1B1", "#0F8074", "#0A7C2E",

"#28AA6C", "#B8D24D", "#EDE447", "#FAB158", "#FDAF91", "#E67E74",

"#FF7777", "#FD0000", "#AD002A", "#792244", "#AD556B", "#AE8691",

"#CE9573", "#B09F91", "#DEB8A1", "#4C4E4E", "#5B4232"), `0` = c("#00468B",

"#5377A7", "#6C6DA4", "#925E9F", "#759EDD", "#0099B4", "#42C1BB",

"#76D1B1", "#0A7C2E", "#B8D24D", "#EDE447", "#FAB158", "#FDAF91",

"#FF7777", "#FD0000", "#AD002A", "#AE8691", "#DEB8A1", "#4C4E4E",

"#5B4232"))), gray = list(default = list(`2` = c("#D8DBE5", "#1B1919"

), `6` = c("#6C3C95", "#FDBF17", "#ABCF8E", "#EE2026", "#2E5696",

"#C8C8CA"), `0` = c("#00468B", "#ED0000", "#42B540", "#0099B4",

"#925E9F", "#FDAF91", "#AD002A", "#ADB6B6", "#1B1919"))), onlinereport = list(

default = list(`0` = c("#CE2930", "#76C5E1", "#D28F26", "#7A1B6C",

"#21A465", "#D4CC38", "#2E0A4A", "#1FABAB", "#E6E191", "#458ECD",

"#891B20", "#3A322F", "#C8CAC9", "#5F64AB", "#B172AC", "#E09FC5",

"#D33C67", "#EB6924", "#A369F4", "#70F2D3", "#6EB933", "#EDAB2D",

"#096D42", "#4EC4B5", "#A36924", "#7350EB", "#891a3a", "#bf109a",

"#E8851F", "#e77def", "#4ebee5", "#69a4f9", "#f9cfa5", "#13D9B1",

"#bfe046", "#bfac88", "#d2f7ba", "#24e4ed", "#A6E2BE", "#c0e9fc",

"#f9efb4", "#fcb4b4", "#e5bcf7", "#acb9ea", "#a7dbd1", "#f2f225",

"#c67997", "#87a822", "#d39279", "#5DA6E8")), stack = list(

`0` = c("#CE2930", "#76C5E1", "#D28F26", "#7A1B6C", "#21A465",

"#D4CC38", "#2E0A4A", "#1FABAB", "#E6E191", "#458ECD",

"#891B20", "#3A322F", "#C8CAC9", "#5F64AB", "#B172AC",

"#E09FC5", "#D33C67", "#EB6924", "#A369F4", "#70F2D3",

"#6EB933", "#EDAB2D", "#096D42", "#4EC4B5", "#A36924",

"#7350EB", "#891a3a", "#bf109a", "#E8851F", "#e77def",

"#4ebee5", "#69a4f9", "#f9cfa5", "#13D9B1", "#bfe046",

"#bfac88", "#d2f7ba", "#24e4ed", "#A6E2BE", "#c0e9fc",

"#f9efb4", "#fcb4b4", "#e5bcf7", "#acb9ea", "#a7dbd1",

"#f2f225", "#c67997", "#87a822", "#d39279", "#5DA6E8"

)), set1 = list(`0` = c("#F0B142", "#62D6F6", "#F14149",

"#28C580", "#FBF73E", "#B02F96", "#1CE8BF", "#F3854F", "#561BB5",

"#C6C5E8", "#7ED8FA", "#FDF791", "#EFD53F", "#E46665", "#4846A6",

"#BEDF50", "#46BBA8", "#acb9ea", "#A17817", "#821762", "#A2E2B3",

"#406BDE", "#0CB1ED", "#51EBFC", "#c4f98c", "#FFDC48", "#FEA8C3",

"#F1835B", "#C4477A", "#821762", "#D544AE", "#50B5F5", "#90D1F4",

"#A0F5F1", "#D1FCE4", "#54C4B5", "#D4E746", "#EDB349", "#FEE05B",

"#F76C50", "#BA2D2D", "#3793DE", "#a7dbd1", "#ede893", "#76cfed",

"#c67997", "#bfac88", "#d39279", "#9e516b", "#666b96")),

set2 = list(`0` = c("#69a4f9", "#bfe046", "#EDAC2E", "#f2f225",

"#a155f9", "#db2830", "#4ec4b5", "#4599de", "#E8851F", "#6FBA33",

"#5F64AC", "#58B984", "#891a3a", "#bf109a", "#f7cb16", "#15ad68",

"#14b5b5", "#4ebee5", "#1a497c", "#EC6925", "#096d42", "#7a1b6c",

"#d33c67", "#db9421", "#ded531", "#521f84", "#87a822", "#43efce",

"#11C2CC", "#fc68c7", "#e77def", "#76cfed", "#125fb2", "#a36924",

"#c67997", "#B271AD", "#acb9ea", "#fcb4b4", "#f9cfa5", "#f9efb4",

"#d2f7ba", "#8bd6d6", "#c5fcdd", "#c0e9fc", "#e5bcf7", "#eda4d3",

"#d39279", "#d6beb2", "#e2a1ba", "#7350EB")), set3 = list(

`0` = c("#bfe046", "#f7cb16", "#fcb4b4", "#e77def", "#acb9ea",

"#24e4ed", "#69a4f9", "#43efce", "#14b5b5", "#125fb2",

"#521f84", "#db2830", "#EC6925", "#f2f225", "#c0e9fc",

"#d2f7ba", "#f9cfa5", "#eda4d3", "#15ad68", "#6FBA33",

"#096d42", "#db9421", "#1a497c", "#7350EB", "#bf109a",

"#d33c67", "#fc68c7", "#EDAC2E", "#ded531", "#87a822",

"#4ec4b5", "#77D7F2", "#a155f9", "#B271AD", "#7a1b6c",

"#a36924", "#c67997", "#E8851F", "#891a3a", "#d39279",

"#e5bcf7", "#5F64AC", "#4599de", "#76cfed", "#e2a1ba",

"#f9efb4", "#d6beb2", "#58B984", "#c5fcdd", "#8bd6d6"

))), tsne = list(default = list(`1` = "#42B540", `2` = c("#42B540",

"#EDE447"), `3` = c("#42B540", "#EDE447", "#FF7777"), `4` = c("#00468B",

"#42B540", "#EDE447", "#FF7777"), `5` = c("#00468B", "#42B540",

"#EDE447", "#759EDD", "#FF7777"), `10` = c("#00468B", "#925E9F",

"#0099B4", "#76D1B1", "#42B540", "#EDE447", "#FAB158", "#FF7777",

"#AD002A", "#759EDD"), `15` = c("#00468B", "#925E9F", "#759EDD",

"#0099B4", "#76D1B1", "#42B540", "#B8D24D", "#EDE447", "#FAB158",

"#FF7777", "#FD0000", "#AD002A", "#AE8691", "#CE9573", "#756455"

), `20` = c("#00468B", "#5377A7", "#6C6DA4", "#925E9F", "#759EDD",

"#0099B4", "#42C1BB", "#76D1B1", "#42B540", "#B8D24D", "#EDE447",

"#FAB158", "#FDAF91", "#FF7777", "#FD0000", "#AD002A", "#792244",

"#AE8691", "#CE9573", "#756455"), `24` = c("#00468B", "#5377A7",

"#3B81AB", "#5C298F", "#6C6DA4", "#925E9F", "#759EDD", "#76C8DC",

"#0099B4", "#42C1BB", "#76D1B1", "#0F8074", "#42B540", "#B8D24D",

"#EDE447", "#FAB158", "#FDAF91", "#FF7777", "#FD0000", "#AD002A",

"#792244", "#AE8691", "#CE9573", "#756455"), `28` = c("#00468B",

"#5377A7", "#3B81AB", "#5C298F", "#6C6DA4", "#925E9F", "#AD729B",

"#759EDD", "#76C8DC", "#0099B4", "#42C1BB", "#76D1B1", "#0F8074",

"#28AA6C", "#42B540", "#B8D24D", "#EDE447", "#FAB158", "#FDAF91",

"#E67E74", "#FF7777", "#FD0000", "#AD002A", "#792244", "#AE8691",

"#CE9573", "#B09F91", "#756455"), `35` = c("#00468B", "#5377A7",

"#3B81AB", "#5C298F", "#6C6DA4", "#925E9F", "#AD729B", "#BF8099",

"#D18E96", "#759EDD", "#76C8DC", "#0099B4", "#42C1BB", "#0F8074",

"#438424", "#28AA6C", "#42B540", "#B8D24D", "#EDE447", "#FCCD94",

"#FAB158", "#DCA30C", "#DD7C06", "#E39C94", "#FDAF91", "#E67E74",

"#FF7777", "#FD0000", "#AD002A", "#792244", "#AF556B", "#AE8691",

"#CE9573", "#B09F91", "#756455"), `0` = c("#00468B", "#5377A7",

"#3B81AB", "#5C298F", "#6C6DA4", "#925E9F", "#AD729B", "#BF8099",

"#D18E96", "#759EDD", "#76C8DC", "#0099B4", "#42C1BB", "#0F8074",

"#438424", "#28AA6C", "#42B540", "#B8D24D", "#EDE447", "#FCCD94",

"#FAB158", "#DCA30C", "#DD7C06", "#E39C94", "#FDAF91", "#E67E74",

"#FF7777", "#FD0000", "#AD002A", "#792244", "#AF556B", "#AE8691",

"#CE9573", "#B09F91", "#756455")), set1 = list(`1` = "#42B540",

`2` = c("#42B540", "#EDE447"), `3` = c("#42B540", "#EDE447",

"#FF7777"), `4` = c("#00468B", "#42B540", "#EDE447", "#FF7777"

), `5` = c("#00468B", "#42B540", "#EDE447", "#759EDD", "#FF7777"

), `10` = c("#00468B", "#925E9F", "#0099B4", "#76D1B1", "#42B540",

"#EDE447", "#FAB158", "#FF7777", "#AD002A", "#759EDD"), `15` = c("#00468B",

"#925E9F", "#759EDD", "#0099B4", "#76D1B1", "#42B540", "#B8D24D",

"#EDE447", "#FAB158", "#FF7777", "#FD0000", "#AD002A", "#AE8691",

"#CE9573", "#756455"), `20` = c("#00468B", "#5377A7", "#6C6DA4",

"#925E9F", "#759EDD", "#0099B4", "#42C1BB", "#76D1B1", "#42B540",

"#B8D24D", "#EDE447", "#FAB158", "#FDAF91", "#FF7777", "#FD0000",

"#AD002A", "#792244", "#AE8691", "#CE9573", "#756455"), `24` = c("#00468B",

"#5377A7", "#3B81AB", "#5C298F", "#6C6DA4", "#925E9F", "#759EDD",

"#76C8DC", "#0099B4", "#42C1BB", "#76D1B1", "#0F8074", "#42B540",

"#B8D24D", "#EDE447", "#FAB158", "#FDAF91", "#FF7777", "#FD0000",

"#AD002A", "#792244", "#AE8691", "#CE9573", "#756455"), `28` = c("#00468B",

"#5377A7", "#3B81AB", "#5C298F", "#6C6DA4", "#925E9F", "#AD729B",

"#759EDD", "#76C8DC", "#0099B4", "#42C1BB", "#76D1B1", "#0F8074",

"#28AA6C", "#42B540", "#B8D24D", "#EDE447", "#FAB158", "#FDAF91",

"#E67E74", "#FF7777", "#FD0000", "#AD002A", "#792244", "#AE8691",

"#CE9573", "#B09F91", "#756455"), `35` = c("#00468B", "#5377A7",

"#3B81AB", "#5C298F", "#6C6DA4", "#925E9F", "#AD729B", "#BF8099",

"#D18E96", "#759EDD", "#76C8DC", "#0099B4", "#42C1BB", "#0F8074",

"#438424", "#28AA6C", "#42B540", "#B8D24D", "#EDE447", "#FCCD94",

"#FAB158", "#DCA30C", "#DD7C06", "#E39C94", "#FDAF91", "#E67E74",

"#FF7777", "#FD0000", "#AD002A", "#792244", "#AF556B", "#AE8691",

"#CE9573", "#B09F91", "#756455"), `0` = c("#00468B", "#5377A7",

"#3B81AB", "#5C298F", "#6C6DA4", "#925E9F", "#AD729B", "#BF8099",

"#D18E96", "#759EDD", "#76C8DC", "#0099B4", "#42C1BB", "#0F8074",

"#438424", "#28AA6C", "#42B540", "#B8D24D", "#EDE447", "#FCCD94",

"#FAB158", "#DCA30C", "#DD7C06", "#E39C94", "#FDAF91", "#E67E74",

"#FF7777", "#FD0000", "#AD002A", "#792244", "#AF556B", "#AE8691",

"#CE9573", "#B09F91", "#756455")), set2 = list(`1` = "#94B2CE",

`2` = c("#94B2CE", "#EBB196"), `3` = c("#94B2CE", "#EBB196",

"#AACDA2"), `4` = c("#B6D1FC", "#EBB196", "#AACDA2", "#E2BDD4"

), `5` = c("#94B2CE", "#EBB196", "#AACDA2", "#E2BDD4", "#AB91BB"

), `10` = c("#94B2CE", "#AEB7C7", "#AACDA2", "#DBB3B5", "#82BDE1",

"#E2BDD4", "#BBB978", "#CDBBDB", "#EBB196", "#AB91BB"), `16` = c("#94B2CE",

"#AEB7C7", "#AACDA2", "#DBB3B5", "#82BDE1", "#887474", "#E2BDD4",

"#BBB978", "#8AA5A2", "#DDB177", "#8892BF", "#DC7FAF", "#78AB91",

"#CDBBDB", "#EBB196", "#AB91BB"), `20` = c("#94B2CE", "#AEB7C7",

"#9B8EAC", "#AACDA2", "#C0767A", "#DBB3B5", "#82BDE1", "#778B8B",

"#887474", "#6D6D6D", "#BBB978", "#8AA5A2", "#DDB177", "#8892BF",

"#DC7FAF", "#78AB91", "#CDBBDB", "#86A0C7", "#EBB196", "#AB91BB"

), `25` = c("#94B2CE", "#AEB7C7", "#9B8EAC", "#AACDA2", "#C0767A",

"#DBB3B5", "#82BDE1", "#778B8B", "#887474", "#B0A9A5", "#E2BDD4",

"#6D6D6D", "#BBB978", "#8AA5A2", "#DDB177", "#8892BF", "#C48ABD",

"#DC7FAF", "#78AB91", "#919E73", "#CDBBDB", "#C08A67", "#86A0C7",

"#EBB196", "#AB91BB"), `30` = c("#94B2CE", "#AEB7C7", "#D0AE90",

"#9B8EAC", "#AACDA2", "#C0767A", "#DBB3B5", "#82BDE1", "#778B8B",

"#887474", "#B0A9A5", "#E2BDD4", "#FFADC9", "#6D6D6D", "#BBB978",

"#C5C6A7", "#8AA5A2", "#A9C3C7", "#DDB177", "#8892BF", "#C48ABD",

"#B6CB95", "#DC7FAF", "#78AB91", "#919E73", "#CDBBDB", "#C08A67",

"#86A0C7", "#EBB196", "#AB91BB"), `35` = c("#799FC2", "#AEB7C7",

"#C4B29A", "#D0AE90", "#9B8EAC", "#AACDA2", "#C0767A", "#D2A0A3",

"#63ACD9", "#B9B1C0", "#887474", "#B0A9A5", "#DBACC9", "#FFADC9",

"#6D6D6D", "#B7B7B9", "#BBB978", "#C5C6A7", "#8AA5A2", "#A9C3C7",

"#DDB177", "#8892BF", "#B1B076", "#C48ABD", "#B6CB95", "#DC7FAF",

"#78AB91", "#CB7C88", "#919E73", "#CDBBDB", "#C08A67", "#86A0C7",

"#EBB196", "#AB91BB", "#DAC097"), `0` = c("#799FC2", "#AEB7C7",

"#C4B29A", "#D0AE90", "#9B8EAC", "#AACDA2", "#C0767A", "#D2A0A3",

"#63ACD9", "#B9B1C0", "#887474", "#B0A9A5", "#DBACC9", "#FFADC9",

"#6D6D6D", "#B7B7B9", "#BBB978", "#C5C6A7", "#8AA5A2", "#A9C3C7",

"#DDB177", "#8892BF", "#B1B076", "#C48ABD", "#B6CB95", "#DC7FAF",

"#78AB91", "#CB7C88", "#919E73", "#CDBBDB", "#C08A67", "#86A0C7",

"#EBB196", "#AB91BB", "#DAC097")), set3 = list(`1` = "#E4A436",

`2` = c("#E4A436", "#48B9D0"), `3` = c("#E4A436", "#48B9D0",

"#A668AC"), `4` = c("#E4A436", "#48B9D0", "#A668AC", "#DFA7A4"

), `5` = c("#E4A436", "#48B9D0", "#A668AC", "#8EC5BF", "#DFA7A4"

), `10` = c("#E4A436", "#BD685F", "#48B9D0", "#83A5CD", "#A668AC",

"#815A6C", "#DFA7A4", "#8EC5BF", "#8E745D", "#CFC4B8"), `14` = c("#E4A436",

"#BD685F", "#48B9D0", "#94DBE0", "#83A5CD", "#A668AC", "#815A6C",

"#ED987F", "#8492B4", "#8EC5BF", "#D31715", "#835B43", "#A28C74",

"#D5CFAA"), `20` = c("#E4A436", "#BD685F", "#6CA3B4", "#48B9D0",

"#94DBE0", "#7CBA92", "#83A5CD", "#A668AC", "#815A6C", "#D6B5B1",

"#ED987F", "#703225", "#8492B4", "#8EC5BF", "#AB877D", "#D31715",

"#B5271D", "#835B43", "#A28C74", "#D5CFAA"), `25` = c("#E4A436",

"#BD685F", "#948689", "#6CA3B4", "#48B9D0", "#94DBE0", "#22ACA8",

"#66A39D", "#7CBA92", "#2073B7", "#83A5CD", "#A668AC", "#D6B5B1",

"#ED987F", "#703225", "#8492B4", "#87A3B8", "#8EC5BF", "#AB877D",

"#D31715", "#B5271D", "#835B43", "#CFC4B8", "#A28C74", "#D5CFAA"

), `30` = c("#E4A436", "#BD685F", "#948689", "#6CA3B4", "#48B9D0",

"#94DBE0", "#22ACA8", "#66A39D", "#7CBA92", "#2073B7", "#83A5CD",

"#A668AC", "#815A6C", "#D6B5B1", "#ED987F", "#703225", "#B37C79",

"#8492B4", "#87A3B8", "#8EC5BF", "#99BFB2", "#AB877D", "#DFA7A4",

"#CE0D09", "#B5271D", "#9C4130", "#8E745D", "#CFC4B8", "#A28C74",

"#D5CFAA"), `35` = c("#E4A436", "#BD685F", "#948689", "#6CA3B4",

"#48B9D0", "#94DBE0", "#22ACA8", "#66A39D", "#1B766C", "#7CBA92",

"#2073B7", "#83A5CD", "#A668AC", "#815A6C", "#D6B5B1", "#ED987F",

"#703225", "#B37C79", "#9E93A7", "#8492B4", "#87A3B8", "#8BB4BB",

"#8EC5BF", "#99BFB2", "#AB877D", "#DFA7A4", "#D31715", "#CE0D09",

"#B5271D", "#9C4130", "#835B43", "#8E745D", "#CFC4B8", "#A28C74",

"#D5CFAA"), `0` = c("#E4A436", "#BD685F", "#948689", "#6CA3B4",

"#48B9D0", "#94DBE0", "#22ACA8", "#66A39D", "#1B766C", "#7CBA92",

"#2073B7", "#83A5CD", "#A668AC", "#815A6C", "#D6B5B1", "#ED987F",

"#703225", "#B37C79", "#9E93A7", "#8492B4", "#87A3B8", "#8BB4BB",

"#8EC5BF", "#99BFB2", "#AB877D", "#DFA7A4", "#D31715", "#CE0D09",

"#B5271D", "#9C4130", "#835B43", "#8E745D", "#CFC4B8", "#A28C74",

"#D5CFAA"))), spot = list(set1 = list(`2` = c("#005EEC",

"#A0CD1E"), `3` = c("#005EEC", "#A0CD1E", "#FFC700"), `4` = c("#005EEC",

"#A0CD1E", "#FFC700", "#FE1100"), `5` = c("#005EEC", "#A0CD1E",

"#FFC700", "#FE1100", "#FF7497"), `6` = c("#005EEC", "#A0CD1E",

"#FFC700", "#FF7B01", "#FE1100", "#FF7497"), `7` = c("#005EEC",

"#00D776", "#A0CD1E", "#FFC700", "#FF7B01", "#FE1100", "#FF7497"

), `8` = c("#005EEC", "#00D776", "#A0CD1E", "#FFC700", "#FF7B01",

"#FE1100", "#FF7497", "#DE00AD"), `16` = c("#005EEC", "#009BB1",

"#00D776", "#50D24A", "#A0CD1E", "#D0CA0F", "#FFC700", "#FFA101",

"#FF7B01", "#FF4601", "#FE1100", "#FF4867", "#FF7497", "#EF3AA2",

"#DE00AD", "#DE005D"), `32` = c("#005EEC", "#2F7AEC", "#009BB1",

"#28B4C8", "#00D776", "#3AEE9D", "#50D24A", "#78D273", "#A0CD1E",

"#B5D94C", "#D0CA0F", "#E5E145", "#FFC700", "#F5D04A", "#FFA101",

"#FFB73D", "#FF7B01", "#FF9330", "#FF4601", "#FF6A33", "#FE1100",

"#E53A2E", "#FF4867", "#FF6680", "#FF7497", "#FFA6BC", "#EF3AA2",

"#EF60B2", "#DE00AD", "#F03DC9", "#DE005D", "#DE2C77"), `50` = c("#004CBE",

"#005EEC", "#2F7AEC", "#5E97EC", "#009BB1", "#28B4C8", "#64BBC8",

"#00D776", "#3AEE9D", "#77EEB9", "#50D24A", "#78D273", "#A0D29D",

"#A0CD1E", "#B5D94C", "#C0D977", "#D0CA0F", "#E5E145", "#E5E273",

"#FFC700", "#F5D04A", "#F5DA7B", "#FFA101", "#FFB73D", "#FFCA70",

"#DF6B00", "#FF7B01", "#FF9533", "#FFAF66", "#FF4601", "#FF6A33",

"#FF9066", "#FE1100", "#E53A2E", "#E5655C", "#FF4867", "#FF6680",

"#FF99AA", "#FF7497", "#FFA6BC", "#FFD9E2", "#EF3AA2", "#EF60B2",

"#EF8FC6", "#DE00AD", "#F03DC9", "#F060D0", "#DE005D", "#DE2C77",

"#DE5991"))), ggsci = list(npg = list(`0` = c("#E64B35", "#4DBBD5",

"#00A087", "#3C5488", "#F39B7F", "#8491B4", "#91D1C2", "#DC0000",

"#7E6148", "#B09C85")), aaas = list(`0` = c("#3B4992", "#EE0000",

"#008B45", "#631879", "#008280", "#BB0021", "#5F559B", "#A20056",

"#808180", "#1B1919")), nejm = list(`0` = c("#BC3C29", "#0072B5",

"#E18727", "#20854E", "#7876B1", "#6F99AD", "#FFDC91", "#EE4C97"

)), lancet = list(`0` = c("#00468B", "#ED0000", "#42B540", "#0099B4",

"#925E9F", "#FDAF91", "#AD002A", "#ADB6B6", "#1B1919")), jama = list(

`0` = c("#374E55", "#DF8F44", "#00A1D5", "#B24745", "#79AF97",

"#6A6599", "#80796B")), jco = list(`0` = c("#0073C2", "#EFC000",

"#868686", "#CD534C", "#7AA6DC", "#003C67", "#8F7700", "#3B3B3B",

"#A73030", "#4A6990")), ucscgb = list(`0` = c("#FF0000", "#FF9900",

"#FFCC00", "#00FF00", "#6699FF", "#CC33FF", "#99991E", "#999999",

"#FF00CC", "#CC0000", "#FFCCCC", "#FFFF00", "#CCFF00", "#358000",

"#0000CC", "#99CCFF", "#00FFFF", "#CCFFFF", "#9900CC", "#CC99FF",

"#996600", "#666600", "#666666", "#CCCCCC", "#79CC3D", "#CCCC99"

)), d3_1 = list(`0` = c("#1F77B4", "#FF7F0E", "#2CA02C", "#D62728",

"#9467BD", "#8C564B", "#E377C2", "#7F7F7F", "#BCBD22", "#17BECF"

)), d3_2 = list(`0` = c("#1F77B4", "#FF7F0E", "#2CA02C", "#D62728",

"#9467BD", "#8C564B", "#E377C2", "#7F7F7F", "#BCBD22", "#17BECF",

"#AEC7E8", "#FFBB78", "#98DF8A", "#FF9896", "#C5B0D5", "#C49C94",

"#F7B6D2", "#C7C7C7", "#DBDB8D", "#9EDAE5")), locuszoom = list(

`0` = c("#D43F3A", "#EEA236", "#5CB85C", "#46B8DA", "#357EBD",

"#9632B8", "#B8B8B8")), igv = list(`0` = c("#5050FF", "#CE3D32",

"#749B58", "#F0E685", "#466983", "#BA6338", "#5DB1DD", "#802268",

"#6BD76B", "#D595A7", "#924822", "#837B8D", "#C75127", "#D58F5C",

"#7A65A5", "#E4AF69", "#3B1B53", "#CDDEB7", "#612A79", "#AE1F63",

"#E7C76F", "#5A655E", "#CC9900", "#99CC00", "#A9A9A9", "#CC9900",

"#99CC00", "#33CC00", "#00CC33", "#00CC99", "#0099CC", "#0A47FF",

"#4775FF", "#FFC20A", "#FFD147", "#990033", "#991A00", "#996600",

"#809900", "#339900", "#00991A", "#009966", "#008099", "#003399",

"#1A0099", "#660099", "#990080", "#D60047", "#FF1463", "#00D68F",

"#14FFB1")), cosmic_1 = list(`0` = c("#2E2A2B", "#CF4E9C", "#8C57A2",

"#358DB9", "#82581F", "#2F509E", "#E5614C", "#97A1A7", "#3DA873",

"#DC9445")), cosmic_2 = list(`0` = c("#171717", "#7D0226", "#300049",

"#165459", "#3F2327", "#0B1948", "#E71012", "#555555", "#193006",

"#A8450C")), cosmic_3 = list(`0` = c("#5ABCEB", "#050708", "#D33C32",

"#CBCACB", "#ABCD72", "#E7C9C6")), uchicago_1 = list(`0` = c("#800000",

"#767676", "#FFA319", "#8A9045", "#155F83", "#C16622", "#8F3931",

"#58593F", "#350E20")), uchicago_2 = list(`0` = c("#800000",

"#D6D6CE", "#FFB547", "#ADB17D", "#5B8FA8", "#D49464", "#B1746F",

"#8A8B79", "#725663")), uchicago_3 = list(`0` = c("#800000",

"#767676", "#CC8214", "#616530", "#0F425C", "#9A5324", "#642822",

"#3E3E23", "#350E20")), startrek = list(`0` = c("#CC0C00", "#5C88DA",

"#84BD00", "#FFCD00", "#7C878E", "#00B5E2", "#00AF66")), tron = list(

`0` = c("#FF410D", "#6EE2FF", "#F7C530", "#95CC5E", "#D0DFE6",

"#F79D1E", "#748AA6")), futurama = list(`0` = c("#FF6F00",

"#C71000", "#008EA0", "#8A4198", "#5A9599", "#FF6348", "#84D7E1",

"#FF95A8", "#3D3B25", "#ADE2D0", "#1A5354", "#3F4041")), rickandmorty = list(

`0` = c("#FAFD7C", "#82491E", "#24325F", "#B7E4F9", "#FB6467",

"#526E2D", "#E762D7", "#E89242", "#FAE48B", "#A6EEE6", "#917C5D",

"#69C8EC")), simpsons = list(`0` = c("#FED439", "#709AE1",

"#8A9197", "#D2AF81", "#FD7446", "#D5E4A2", "#197EC0", "#F05C3B",

"#46732E", "#71D0F5", "#370335", "#075149", "#C80813", "#91331F",

"#1A9993", "#FD8CC1")), gsea = list(`0` = c("#4500AC", "#2600D1",

"#6B58EE", "#8787FF", "#C6C0FF", "#D4D4FF", "#FFBFE5", "#FF8888",

"#FF707F", "#FF5959", "#EE3F3F", "#D60C00"))))

color.yaml <-

"Colors.yml"

core_otsu <-

function (dt)

{

weight.bg <- sapply(seq(0, max(dt)), function(i) sum(dt <=

i)/length(dt))

weight.fg <- 1 - weight.bg

mean.bg <- sapply(seq(0, max(dt)), function(i) mean(dt[dt <=

i]))

mean.fg <- sapply(seq(0, max(dt)), function(i) mean(dt[dt >

i]))

var.between <- weight.bg * weight.fg * (mean.bg - mean.fg)^2

thres <- which.max(var.between)

return(thres)

}

DoCellCycleScoring <-

function (object, s.genes = NULL, g2m.genes = NULL, ...)

{

if (is.null(s.genes))

s.genes <- cc.genes$s.genes

if (is.null(g2m.genes))

g2m.genes <- cc.genes$g2m.genes

s.genes <- FindFeaturesID(object, s.genes)

g2m.genes <- FindFeaturesID(object, g2m.genes)

if (length(s.genes) < 2 || length(g2m.genes) < 2)

return(object)

object <- CellCycleScoring(object, s.features = s.genes,

g2m.features = g2m.genes, set.ident = FALSE, ...)

object[["CC.Difference"]] <- object[["S.Score"]] - object[["G2M.Score"]]

return(object)

}

DoDimReduc <-

function (object, assay = NULL, pc.num = 50, is.checkpca = TRUE,

n.components = 2L, reduction = NULL, reduction.surfix = NULL,

dims = NULL, nn.name = NULL, is.tsne = TRUE, is.UMAP = TRUE,

...)

{

if (is.null(assay))

assay <- DefaultAssay(object)

if (is.null(reduction))

reduction <- "pca"

if (is.null(reduction.surfix))

reduction.surfix <- assay

if (reduction == "pca" && is.null(nn.name)) {

message("-->PCA<--")

pc.num <- min(pc.num, ncol(object) - 1)

object <- RunPCA(object = object, assay = assay, npcs = pc.num,

features = VariableFeatures(object), verbose = FALSE)

object[[paste0("pca_", reduction.surfix)]] <- object[["pca"]]

if (is.checkpca)

CheckPCA(object)

reduction <- "pca"

}

pc.num <- ncol(object[[reduction]])

sig.PCs <- if (is.null(dims))

seq(pc.num)

else dims

if (is.tsne && is.null(nn.name)) {

object <- DoRunTSNE(object, dims = sig.PCs, reduction = reduction,

nn.name = nn.name, reduction.surfix = reduction.surfix,

n.components = n.components, ...)

}

if (is.UMAP) {

object <- DoRunUMAP(object, dims = sig.PCs, reduction = reduction,

nn.name = nn.name, reduction.surfix = reduction.surfix,

n.components = n.components)

}

return(object)

}

DoFindAllMarkers <-

function (object, parameter = list(), group.by = "seurat_clusters",

min.pct = eval(parse(text = IfNull(parameter$FindMarkers$min_pct,

0.25))), logfc.threshold = eval(parse(text = IfNull(parameter$FindMarkers$logfc,

0.25))), return.thresh = eval(parse(text = IfNull(parameter$FindMarkers$pvalue,

0.01))), pseudocount.use = eval(parse(text = IfNull(parameter$FindMarkers$pseudocount.use,

0))), only.pos = eval(parse(text = IfNull(parameter$FindMarkers$only.pos,

TRUE))), base = exp(1), ...)

{

Idents(object) <- group.by

if (packageVersion("Seurat") < as.numeric_version("4.0.0")) {

object.markers <- FindAllMarkers(object = object, only.pos = only.pos,

min.pct = min.pct, logfc.threshold = logfc.threshold,

return.thresh = return.thresh, pseudocount.use = pseudocount.use,

...)

}

else {

object.markers <- FindAllMarkers(object = object, only.pos = only.pos,

min.pct = min.pct, logfc.threshold = logfc.threshold,

return.thresh = return.thresh, pseudocount.use = pseudocount.use,

base = base, ...)

}

if (nrow(object.markers) == 0) {

object.markers <- data.frame(p_val = 1, avg_logFC = 1,

pct.1 = 1, pct.2 = 1, p_val_adj = 1, cluster = "1",

gene = 1)

object.markers <- object.markers[-1, ]

}

return(object.markers)

}

DoFindClusters <-

function (object, reduction = "pca", dims = NULL, resolution = 0.5,

algorithm = 1, color.save = "color.cluster", prune.SNN = 1/15)

{

if (is.null(dims) || max(dims) > length(object[[reduction]]))

dims <- seq(object[[reduction]])

object <- FindNeighbors(object = object, reduction = reduction,

dims = dims, force.recalc = TRUE, prune.SNN = prune.SNN)

object <- FindClusters(object = object, resolution = resolution,

algorithm = algorithm, temp.file.location = getwd())

object@misc[[color.save]] <- SetColor(object@meta.data$seurat_clusters,

"tsne", "set1")

return(object)

}

DoFindClusters.WNN <-

function (object, reduction.list = NULL, dims.list = NULL, resolution = 0.5,

algorithm = 1, color.save = "color.cluster", ...)

{

object <- FindMultiModalNeighbors(object = object, modality.weight.name = "weight.nn",

reduction.list = reduction.list, dims.list = dims.list,

...)

object <- FindClusters(object = object, resolution = resolution,

algorithm = algorithm, graph.name = "wsnn", temp.file.location = getwd())

object@misc[[color.save]] <- SetColor(object@meta.data$seurat_clusters,

"tsne", "set1")

return(object)

}

DoIntegration <-

function (object, split.by = "orig.ident", dims = 1:50, nfeatures = 3000,

is.SCT = FALSE, normalization.method = "LogNormalize")

{

old.assay <- DefaultAssay(object)

object.list <- SplitObject(object, split.by = split.by)

object.list <- SplitObject.Image(object.list)

anchor.features <- nfeatures

if (is.SCT) {

for (i in seq(object.list)) {

object.list[[i]] <- SCTransform(object.list[[i]],

vars.to.regress = object@misc$vars.regress, verbose = FALSE,

assay = old.assay)

}

anchor.features <- SelectIntegrationFeatures(object.list = object.list,

nfeatures = nfeatures)

object.list <- PrepSCTIntegration(object.list = object.list,

anchor.features = anchor.features)

normalization.method <- "SCT"

}

k.filter <- min(200, ceiling(min(sapply(object.list, ncol))/2))

if (any(sapply(object.list, ncol) <= max(dims))) {

message(paste(sapply(object.list, ncol), collapse = " "))

dims <- seq(min(sapply(object.list, ncol)) - 1)

}

anchors <- FindIntegrationAnchors(object.list = object.list,

dims = dims, normalization.method = normalization.method,

anchor.features = anchor.features, k.filter = k.filter)

if (nrow(anchors@anchors) == 0) {

message("[Integrate] anchors is 0. No Integrating.")

return(object)

}

integrated <- IntegrateData(anchorset = anchors, dims = dims,

normalization.method = normalization.method, k.weight = k.filter)

if (!is.SCT) {

integrated <- ScaleData(integrated, verbose = FALSE,

vars.to.regress = object@misc$vars.regress)

}

integrated@misc <- object@misc

integrated[[old.assay]] <- object[[old.assay]]

integrated@reductions <- object@reductions

integrated@meta.data <- object@meta.data

return(integrated)

}

DoNormalization <-

function (object, parameter = list(), assay = "RNA", is_SCTransform = FALSE,

vars.regress = NULL, is.check = TRUE, nfeatures = 2000, normalization.method = "LogNormalize",

sct.method = "poisson", scale.only.var.genes = FALSE, vfeatures = parameter$marker$vfeatures,

vfeature.must = NULL, vfeature.remove = NULL, ...)

{

DefaultAssay(object) <- assay

object <- FindRegressVars(object, parameter, vars.regress = vars.regress,

...)

if (is_SCTransform) {

object <- SCTransform(object, assay = assay, vars.to.regress = object@misc$vars.regress,

verbose = FALSE, min_cells = 1, method = sct.method,

return.only.var.genes = scale.only.var.genes)

VariableFeatures(object) <- setdiff(VariableFeatures(object),

vfeature.remove)

}

else {

message("-->Normalize Data<--")

object <- NormalizeData(object, normalization.method = normalization.method,

scale.factor = 10000)

message("-->Find Variable Genes<--")

object <- FindVariableFeatures(object, selection.method = "vst",

nfeatures = nfeatures)

VariableFeatures(object) <- union(VariableFeatures(object),

vfeature.must)

VariableFeatures(object) <- setdiff(VariableFeatures(object),

vfeature.remove)

if (!is.null(vfeatures))

VariableFeatures(object) <- vfeatures

if (is.check)

CheckVariableFeature(object)

message("-->ScaleData<--")

features <- if (scale.only.var.genes)

NULL

else rownames(object)

object <- ScaleData(object = object, vars.to.regress = object@misc$vars.regress,

features = features)

}

return(object)

}

DoRunTSNE <-

function (object, dims = 1:50, reduction = "pca", nn.name = NULL,

reduction.surfix = NULL, n.components = 2L, ...)

{

message("-->Run tSNE<--")

perplexity <- min(30, floor((ncol(object) - 1)/3))

reduction.name <- if (n.components == 2)

"tsne"

else paste0("tsne", n.components)

dims <- intersect(dims, seq(object[[reduction]]))

object <- RunTSNE(object, dims = dims, dim.embed = n.components,

perplexity = perplexity, reduction.name = reduction.name,

reduction = reduction, ...)

if (!is.null(reduction.surfix)) {

object[[paste0(reduction.name, "_", reduction.surfix)]] <- object[[reduction.name]]

}

return(object)

}

DoRunUMAP <-

function (object, dims = 1:50, reduction = "pca", nn.name = NULL,

reduction.surfix = NULL, n.components = 2L, umap.method = "uwot",

...)

{

message("-->Run UMAP<--")

n.neighbors <- min(30, length(dims))

reduction.name <- if (n.components == 2)

"umap"

else paste0("umap", n.components)

dims <- intersect(dims, seq(object[[reduction]]))

if (is.null(nn.name)) {

object <- RunUMAP(object, dims = dims, umap.method = umap.method,

n.neighbors = n.neighbors, n.components = n.components,

reduction.name = reduction.name, reduction = reduction,

...)

}

else {

object <- RunUMAP(object, umap.method = umap.method,

n.neighbors = n.neighbors, n.components = n.components,

reduction.name = reduction.name, nn.name = nn.name,

...)

}

if (!is.null(reduction.surfix)) {

object[[paste0(reduction.name, "_", reduction.surfix)]] <- object[[reduction.name]]

}

return(object)

}

dot_theme_default <-

function (font_use = "Arial")

{

library(ggplot2)

mytheme <- theme_bw() + theme(panel.grid.major = element_blank(),

panel.grid.minor = element_blank(), panel.border = element_rect(color = "#000000",

size = 0.80000000000000004), axis.text = element_text(color = "#000000",

size = 11), axis.text.x = element_text(angle = 0,

hjust = 0.5, vjust = 0.5), axis.text.y = element_text(hjust = 0.5,

vjust = 0.5), axis.title = element_text(color = "#000000",

size = 14, face = "plain"), axis.title.x = element_text(margin = margin(2.5,

0, 2.5, 0, "mm")), axis.title.y = element_text(margin = margin(0,

2.5, 0, 2.5, "mm")), axis.ticks = element_line(color = "#000000",

size = 0.5), axis.ticks.length = unit(0.10000000000000001,

"cm"), legend.title = element_blank(), legend.text = element_text(size = 11),

plot.title = element_text(color = "#000000", size = 16,

face = "plain", hjust = 0.5), plot.margin = unit(c(5,

5, 5, 5), "mm"))

if (font_use != "" & "extrafont" %in% installed.packages()) {

library(extrafont)

library(extrafontdb)

library(Rttf2pt1)

if (font_use %in% fonts()) {

mytheme <- mytheme + theme(text = element_text(family = font_use))

}

}

if (FALSE & font_use == "Arial" & "Cairo" %in% installed.packages()) {

library(Cairo)

CairoFonts(regular = "Arial:style=Regular", bold = "Arial:style=Bold",

italic = "Arial:style=Italic", bolditalic = "Arial:style=Bold Italic,BoldItalic")

source(ggsave_R)

}

if (FALSE & "showtext" %in% installed.packages()) {

library(showtext)

showtext_auto(enable = TRUE)

font_add("Arial", regular = arial_ttf)

mytheme <- mytheme + theme(text = element_text(family = "Arial"))

}

mytheme

}

dot_theme_manhattan <-

function (font_use = "Arial")

{

library(ggplot2)

mytheme <- theme_bw() + theme(panel.grid.major = element_blank(),

panel.grid.minor = element_blank(), panel.border = element_rect(color = "#000000",

size = 0.80000000000000004), axis.text = element_text(color = "#000000",

size = 11), axis.text.x = element_text(angle = 0,

hjust = 0.5, vjust = 0.5), axis.text.y = element_text(hjust = 1,

vjust = 0.5), axis.title = element_text(color = "#000000",

size = 14, face = "bold"), axis.title.x = element_text(margin = margin(2.5,

0, 2.5, 0, "mm")), axis.title.y = element_text(margin = margin(0,

2.5, 0, 2.5, "mm")), axis.ticks = element_line(color = "#000000",

size = 0.5), axis.ticks.length = unit(0.10000000000000001,

"cm"), legend.title = element_blank(), legend.justification = "center",

plot.title = element_text(color = "#000000", size = 16,

face = "bold", hjust = 0.5), plot.margin = unit(c(5,

5, 5, 5), "mm"))

if (font_use != "" & "extrafont" %in% installed.packages()) {

library(extrafont)

library(extrafontdb)

library(Rttf2pt1)

if (font_use %in% fonts()) {

mytheme <- mytheme + theme(text = element_text(family = font_use))

}

}

mytheme

}

DotPlot <-

function (object, assay = NULL, features, cols = c("lightgrey",

"blue"), col.min = -2.5, col.max = 2.5, dot.min = 0, dot.scale = 6,

group.by = NULL, split.by = NULL, split.fade.scale = 0.80000000000000004,

scale.by = "radius", scale.min = NA, scale.max = NA)

{

assay <- assay %||% DefaultAssay(object = object)

DefaultAssay(object = object) <- assay

scale.func <- switch(EXPR = scale.by, size = scale_size,

radius = scale_radius, stop("'scale.by' must be either 'size' or 'radius'"))

data.features <- FetchData(object = object, vars = features)

data.features$id <- if (is.null(x = group.by)) {

Idents(object = object)

}

else {

object[[group.by, drop = TRUE]]

}

if (!is.factor(x = data.features$id)) {

data.features$id <- factor(x = data.features$id)

}

id.levels <- levels(x = data.features$id)

data.features$id <- as.vector(x = data.features$id)

if (!is.null(x = split.by)) {

splits <- object[[split.by, drop = TRUE]]

if (!is.factor(x = splits)) {

splits <- factor(splits)

}

if (nlevels(x = splits) > length(x = cols)) {

stop("Not enought colors for the number of groups")

}

cols <- cols[seq(levels(x = splits))]

names(x = cols) <- levels(x = splits)

cols.split <- cols

data.features$id <- paste(data.features$id, splits, sep = "_")

id.levels <- paste0(rep(x = id.levels, each = nlevels(x = splits)),

"_", rep(x = levels(x = splits), times = length(x = id.levels)))

tmp <- unique(data.frame(id = data.features$id, splits))

cols <- cols[tmp$splits]

names(cols) <- tmp$id

}

data.plot <- lapply(X = unique(x = data.features$id), FUN = function(ident) {

data.use <- data.features[data.features$id == ident,

1:(ncol(x = data.features) - 1), drop = FALSE]

avg.exp <- apply(X = data.use, MARGIN = 2, FUN = function(x) {

return(mean(x = expm1(x = x)))

})

pct.exp <- apply(X = data.use, MARGIN = 2, FUN = PercentAbove,

threshold = 0)

return(list(avg.exp = avg.exp, pct.exp = pct.exp))

})

names(x = data.plot) <- unique(x = data.features$id)

data.plot <- lapply(X = names(x = data.plot), FUN = function(x) {

data.use <- as.data.frame(x = data.plot[[x]])

data.use$features.plot <- rownames(x = data.use)

data.use$id <- x

return(data.use)

})

data.plot <- do.call(what = "rbind", args = data.plot)

if (!is.null(x = id.levels)) {

data.plot$id <- factor(x = data.plot$id, levels = id.levels)

}

if (nlevels(data.plot$id) == 1) {

avg.exp.scaled <- scale(data.plot$avg.exp)

avg.exp.scaled <- MinMax(data = avg.exp.scaled, min = col.min,

max = col.max)

}

else {

avg.exp.scaled <- sapply(X = unique(x = data.plot$features.plot),

FUN = function(x) {

data.use <- data.plot[data.plot$features.plot ==

x, "avg.exp"]

data.use <- scale(x = data.use)

data.use <- MinMax(data = data.use, min = col.min,

max = col.max)

return(data.use)

})

}

avg.exp.scaled <- as.vector(x = t(x = avg.exp.scaled))

if (!is.null(x = split.by)) {

color.index <- as.numeric(x = cut(x = avg.exp.scaled,

breaks = 100))

}

data.plot$avg.exp.scaled <- avg.exp.scaled

data.plot$features.plot <- factor(x = data.plot$features.plot,

levels = rev(x = features))

data.plot$pct.exp[data.plot$pct.exp < dot.min] <- NA

data.plot$pct.exp <- data.plot$pct.exp * 100

if (!is.null(x = split.by)) {

split.col.function <- function(color, value) {

color.hsv <- rgb2hsv(col2rgb(color))

color.hsv[2, ] <- color.hsv[2, ] * (1 - split.fade.scale)

min.color <- hsv(color.hsv[1, ], color.hsv[2, ],

color.hsv[3, ])

return(colorRampPalette(colors = c(min.color, color))(100)[value])

}

data.plot$colors <- mapply(FUN = split.col.function,

color = cols[as.character(data.plot$id)], value = color.index)

}

color.by <- ifelse(test = is.null(x = split.by), yes = "avg.exp.scaled",

no = "colors")

if (!is.na(x = scale.min)) {

data.plot[data.plot$pct.exp < scale.min, "pct.exp"] <- scale.min

}

if (!is.na(x = scale.max)) {

data.plot[data.plot$pct.exp > scale.max, "pct.exp"] <- scale.max

}

plot <- ggplot(data = data.plot, mapping = aes_string(x = "features.plot",

y = "id")) + geom_point(mapping = aes_string(size = "pct.exp",

color = color.by)) + scale.func(range = c(0, dot.scale),

limits = c(scale.min, scale.max)) + theme(axis.title.x = element_blank(),

axis.title.y = element_blank()) + guides(size = guide_legend(title = "Percent Expressed")) +

labs(x = "Features", y = ifelse(test = is.null(x = split.by),

yes = "Identity", no = "Split Identity")) + theme_cowplot()

if (!is.null(x = split.by)) {

plot <- plot + scale_color_identity() + geom_polygon(aes(x = 0,

y = 0, fill = avg.exp.scaled)) + scale_fill_gradientn(colours = sapply(split.col.function(cols.split[1]),

col2grey)) + geom_point(aes(x = 0, y = 0, alpha = avg.exp.scaled),

size = 0) + scale_alpha("contrast", range = c(1,

1), limits = c(0, 1), breaks = seq(0, 1, length.out = length(cols.split)),

labels = names(cols.split), guide = guide_legend(override.aes = list(color = cols.split,

size = 3)))

}

else if (length(x = cols) == 1) {

plot <- plot + scale_color_distiller(palette = cols)

}

else {

plot <- plot + scale_color_gradient(low = cols[1], high = cols[2])

}

if (is.null(x = split.by)) {

plot <- plot + guides(color = guide_colorbar(title = "Average Expression"))

}

return(plot)

}

FeatureScatterShell <-

function (object, feature1 = NULL, feature2 = NULL, outfile = NULL,

group.by = "orig.ident", cols = NULL, span = NULL, standard = NULL)

{

p <- FeatureScatter(object = object, feature1 = feature1,

feature2 = feature2, span = span, group.by = group.by,

pt.size = 0.10000000000000001, cols = cols) + guides(color = guide_legend(override.aes = list(size = 2),

title = NULL))

if (!is.null(standard)) {

if (!is.null(standard[[feature1]])) {

p <- p + geom_vline(xintercept = standard[[feature1]],

color = "red")

}

if (!is.null(standard[[feature2]])) {

p <- p + geom_hline(yintercept = standard[[feature2]],

color = "red")

}

}

p <- p + dot_theme_default()

ggsave(p, file = outfile, width = 7, height = 6)

}

fetch_color <-

function (n = 0, type = c(names(color.list), "random"), tag = NULL,

is.extend = TRUE, verbose = FALSE)

{

type <- match.arg(type)

if (type == "random") {

color.use <- fetch_random_color(n = n, usepalette = T)

if (verbose) {

message("color : ", type, "->", tag, "->", n)

}

return(color.use)

}

tag <- match.arg(tag, names(color.list[[type]]))

if (!is.numeric(n))

stop("'n' must be numeric.")

n.available <- as.numeric(names(color.list[[type]][[tag]]))

n.select <- n.available[n.available >= n]

if (length(n.select)) {

if (n == min(n.select)) {

n.select <- as.character(min(n.select))

color.use <- color.list[[type]][[tag]][[n.select]]

}

else {

n.select <- as.character(min(n.select))

color.use <- head(color.list[[type]][[tag]][[n.select]],

n)

}

}

else {

n.select <- as.character(0)

color.use <- color.list[[type]][[tag]][[n.select]]

if (is.extend)

color.use <- colorRampPalette(color.list[[type]][[tag]][[n.select]])(n)

}

if (verbose) {

message("color : ", type, "->", tag, "->", n.select)

}

return(color.use)

}

fetch_random_color <-

function (n = 1, usepalette = FALSE, hue = " ", luminosity = " ")

{

library(randomcoloR)

if (usepalette == TRUE) {

set.seed(1)

color.use <- distinctColorPalette(k = n)

}

else {

color.use <- randomColor(count = n, hue = hue, luminosity = luminosity)

}

return(color.use)

}

FilterCells <-

function (object, parameter = list(), do.stat = TRUE, set.num = IfNull(parameter$filter$set.num,

"none"), set.num.seed = IfNull(parameter$filter$set.num.seed,

42), standard = parameter$filter$standard, filter.cells = parameter$filter$filter.cells,

record_file = "filtered_used_parameter.yaml", ...)

{

message("--->Filter Cells<---")

if (!is.null(filter.cells) && file.exists(filter.cells)) {

filter.cells <- readLines(filter.cells)

}

cells.use <- .FilterCells(object, set.num = set.num, set.num.seed = set.num.seed,

standard = standard, filter.cells = filter.cells, record_file = record_file)

object <- object[, cells.use]

if (do.stat) {

StatFilterCells(object, ...)

}

return(object)

}

FilterGenes <-

function (object, parameter = list(), min.cell = IfNull(parameter$filter$min.cell,

0), assay = NULL)

{

message("--->Filter Genes<---")

if (min.cell > 0) {

if (is.null(assay))

assay <- DefaultAssay(object)

DefaultAssay(object) <- assay

if (min.cell < 1) {

min.cell <- min.cell * length(object@cell.names)

}

num.cells <- Matrix::rowSums(object@assays[[assay]]@counts >

0)

genes.filter <- num.cells[which(num.cells < min.cell)]

genes.filter <- FindFeaturesName(object, genes.filter)

write.table(genes.filter, file = "filtered_genes.xls",

quote = F, sep = "\t", col.name = F)

genes.use <- names(num.cells[which(num.cells >= min.cell)])

object <- object[genes.use, ]

}

return(object)

}

FindFeaturesID <-

function (object, features, unlist = TRUE)

{

object@misc$fdata <- AddUnderscore(object@misc$fdata)

if (all(rownames(object) %in% object@misc$fdata$dash)) {

rownames(object@misc$fdata) <- object@misc$fdata$dash

}

features <- sapply(X = features, FUN = function(x) {

if (!exists("fdata", object@misc))

return(NULL)

g1 <- toupper(rownames(object@misc$fdata)) %in% toupper(x)

if (sum(g1) > 0)

return(rownames(object@misc$fdata)[g1])

g2 <- toupper(object@misc$fdata$name) %in% toupper(x)

if (sum(g2) > 0)

return(rownames(object@misc$fdata)[g2])

g3 <- toupper(object@misc$fdata$merge_name) %in% toupper(x)

if (sum(g3) > 0)

return(rownames(object@misc$fdata)[g3])

g4 <- toupper(object@misc$fdata$underscore) %in% toupper(x)

if (sum(g4) > 0)

return(rownames(object@misc$fdata)[g4])

message("[WARNING] '", x, "' not found gene id.")

return(NULL)

})

if (unlist)

features <- unlist(features)

return(features)

}

FindFeaturesName <-

function (object, features, col = "merge_name", is.fast = FALSE)

{

if (!exists("fdata", object@misc))

return(features)

object@misc$fdata <- AddUnderscore(object@misc$fdata)

new <- gsub("_", "-", features)

if (all(new %in% object@misc$fdata$dash)) {

rownames(object@misc$fdata) <- object@misc$fdata$dash

features <- new

}

if (is.fast) {

Name <- object@misc$fdata[features, col]

names(Name) <- features

}

else {

Name <- sapply(features, function(x) {

id <- object@misc$fdata[x, col]

ifelse(is.null(id) || is.na(id), x, id)

})

}

return(Name)

}

FindRegressVars <-

function (object, parameter = list(), vars.regress = NULL, force_recal = FALSE,

is_rm_cc = IfNull(parameter$cell_cycle$is_remove, TRUE),

is_rm_all_cc_signal = IfNull(parameter$cell_cycle$is_rm_all_signal,

FALSE), ...)

{

if (is.null(vars.regress)) {

vars.regress <- paste0("nCount_", DefaultAssay(object))

if (exists("percent.mito", object@meta.data)) {

vars.regress <- c(vars.regress, "percent.mito")

}

if (is_rm_cc) {

message("-->CellCycle Scoring<--")

if (!exists("CC.Difference", object@meta.data) ||

force_recal) {

object <- DoCellCycleScoring(object, ...)

}

if (is_rm_all_cc_signal) {

for (i in c("S.Score", "G2M.Score")) {

if (exists(i, object@meta.data)) {

vars.regress <- c(vars.regress, i)

}

}

}

else {

if (exists("CC.Difference", object@meta.data)) {

vars.regress <- c(vars.regress, "CC.Difference")

}

}

}

}

else if (vars.regress == "none") {

vars.regress <- NULL

}

object@misc$vars.regress <- vars.regress

return(object)

}

FindTopMarker <-

function (object.markers, top_num = 20, object = NULL, outfile = "Top.avg_exp.xls.tmp")

{

logfc_name <- grep("avg_log", colnames(object.markers), value = T)[1]

logfc_name <- as.name(logfc_name)

top <- object.markers %>% group_by(cluster) %>% arrange(desc(!!logfc_name),

p_val, p_val_adj, .by_group = TRUE) %>% filter(1:n() <=

top_num)

if (!is.null(outfile)) {

tmp <- top %>% select(Cluster = cluster, Gene_ID = gene)

if (!is.null(object)) {

tmp$Gene_ID <- ChangeOUTName(tmp$Gene_ID, object@misc$fdata)

}

WriteTable(tmp, outfile)

}

return(top)

}

FindWHNum <-

function (data, ncol = NULL, nrow = NULL, by.col = TRUE)

{

if (length(data) == 1) {

if (!is.numeric(data))

stop()

data <- round(data)

}

else {

data <- length(data)

}

if (!is.null(ncol) && !is.null(nrow)) {

if (ncol * nrow < data)

stop()

}

else if (!is.null(ncol)) {

nrow <- ceiling(data/ncol)

}

else if (!is.null(nrow)) {

ncol <- ceiling(data/nrow)

}

else {

if (by.col) {

ncol <- ceiling(sqrt(data))

nrow <- ceiling(data/ncol)

}

else {

nrow <- ceiling(sqrt(data))

ncol <- ceiling(data/nrow)

}

}

return(c(nrow, ncol))

}

ggsave_R <-

"src/Rlib/ggsave.R"

heatmap_theme_default <-

function (font_use = "Arial")

{

library(ggplot2)

mytheme <- theme_void() + theme(axis.text = element_text(color = "#000000",

size = 11), axis.text.x = element_text(angle = 45, hjust = 1,

vjust = 1), axis.text.y = element_text(hjust = 0, vjust = 0.5),

legend.title = element_blank(), legend.justification = c(0.5,

0.5), legend.margin = margin(5, 5, 5, 5), legend.text = element_text(hjust = 0.5,

vjust = 0.5, margin = margin(2, 0, 0, 0)), plot.title = element_text(size = 16,

face = "plain", hjust = 0.5), plot.margin = unit(c(5,

5, 5, 5), "mm"))

if (font_use != "" & "extrafont" %in% installed.packages()) {

library(extrafont)

library(extrafontdb)

library(Rttf2pt1)

if (font_use %in% fonts()) {

mytheme <- mytheme + theme(text = element_text(family = font_use))

}

}

if (FALSE & font_use == "Arial" & "Cairo" %in% installed.packages()) {

library(Cairo)

CairoFonts(regular = "Arial:style=Regular", bold = "Arial:style=Bold",

italic = "Arial:style=Italic", bolditalic = "Arial:style=Bold Italic,BoldItalic")

source(ggsave_R)

}

if (FALSE & "showtext" %in% installed.packages()) {

library(showtext)

showtext_auto(enable = TRUE)

font_add("Arial", regular = arial_ttf)

mytheme <- mytheme + theme(text = element_text(family = "Arial"))

}

mytheme

}

heatmap_theme_ggcor <-

function (font_use = "Arial")

{

library(ggplot2)

library(ggcor)

mytheme <- theme_cor() + theme(axis.text = element_text(color = "#000000",

size = 11), legend.title = element_blank(), legend.text = element_text(hjust = 0.5,

vjust = 0.5, margin = margin(2, 0, 0, 0)), plot.title = element_text(size = 16,

face = "plain", hjust = 0.5), )

if (font_use != "" & "extrafont" %in% installed.packages()) {

library(extrafont)

library(extrafontdb)

library(Rttf2pt1)

if (font_use %in% fonts()) {

mytheme <- mytheme + theme(text = element_text(family = font_use))

}

}

if (FALSE & font_use == "Arial" & "Cairo" %in% installed.packages()) {

library(Cairo)

CairoFonts(regular = "Arial:style=Regular", bold = "Arial:style=Bold",

italic = "Arial:style=Italic", bolditalic = "Arial:style=Bold Italic,BoldItalic")

source(ggsave_R)

}

if (FALSE & "showtext" %in% installed.packages()) {

library(showtext)

showtext_auto(enable = TRUE)

font_add("Arial", regular = arial_ttf)

mytheme <- mytheme + theme(text = element_text(family = "Arial"))

}

mytheme

}

hist_theme_default <-

function (font_use = "Arial")

{

library(ggplot2)

mytheme <- theme_bw() + theme(panel.grid.major = element_blank(),

panel.grid.minor = element_blank(), panel.border = element_rect(color = "#000000",

size = 0.80000000000000004), axis.text = element_text(color = "#000000",

size = 11), axis.text.x = element_text(angle = 0,

hjust = 0.5, vjust = 0.5), axis.text.y = element_text(hjust = 1,

vjust = 0.5), axis.title = element_text(color = "#000000",

size = 14, face = "plain"), axis.title.x = element_text(margin = margin(2.5,

0, 2.5, 0, "mm")), axis.title.y = element_text(margin = margin(0,

2.5, 0, 2.5, "mm")), axis.ticks = element_line(color = "#000000",

size = 0.5), axis.ticks.length = unit(0.10000000000000001,

"cm"), legend.title = element_blank(), strip.text = element_blank(),

plot.title = element_text(size = 16, face = "plain",

hjust = 0.5), plot.margin = unit(c(5, 5, 5, 5), "mm"))

if (font_use != "" & "extrafont" %in% installed.packages()) {

library(extrafont)

library(extrafontdb)

library(Rttf2pt1)

if (font_use %in% fonts()) {

mytheme <- mytheme + theme(text = element_text(family = font_use))

}

}

if (FALSE & font_use == "Arial" & "Cairo" %in% installed.packages()) {

library(Cairo)

CairoFonts(regular = "Arial:style=Regular", bold = "Arial:style=Bold",

italic = "Arial:style=Italic", bolditalic = "Arial:style=Bold Italic,BoldItalic")

source(ggsave_R)

}

if (FALSE & "showtext" %in% installed.packages()) {

library(showtext)

showtext_auto(enable = TRUE)

font_add("Arial", regular = arial_ttf)

mytheme <- mytheme + theme(text = element_text(family = "Arial"))

}

mytheme

}

IfNull <-

function (var, default = NULL)

{

if (is.null(var)) {

return(default)

}

else {

return(var)

}

}

KDE <-

function (x, y, z = NULL, h, n = 25, ...)

{

hx <- MASS::bandwidth.nrd(x)

hy <- MASS::bandwidth.nrd(y)

if (hx <= 0)

hx <- diff(range(x))/n

if (hy <= 0)

hy <- diff(range(y))/n

if (is.null(z)) {

if (missing(h))

h <- c(hx, hy)

d <- MASS::kde2d(x = x, y = y, h = h, n = n, ...)

gr <- data.frame(with(d, expand.grid(x, y)), as.vector(d$z))

colnames(gr) <- c("xgr", "ygr", "zgr")

mod <- loess(zgr ~ xgr * ygr, data = gr)

dens <- predict(mod, newdata = data.frame(xgr = x, ygr = y))

}

else {

hz <- MASS::bandwidth.nrd(z)

if (hz <= 0)

hz <- diff(range(z))/n

if (missing(h))

h <- c(hx, hy, hz)

d <- kde3d(x, y, z, h, n = n, ...)

gr <- data.frame(with(d, expand.grid(x, y, z)), as.vector(d$d))

colnames(gr) <- c("xgr", "ygr", "zgr", "dgr")

mod <- loess(dgr ~ xgr * ygr * zgr, data = gr)

dens <- predict(mod, newdata = data.frame(xgr = x, ygr = y,

zgr = z))

}

return(dens)

}

kde3d <-

function (x, y, z, h, n = 20, lims = c(range(x), range(y), range(z)))

{

nx <- length(x)

if (length(y) != nx || length(z) != nx)

stop("data vectors must be the same length")

if (missing(h))

h <- c(MASS::bandwidth.nrd(x), MASS::bandwidth.nrd(y),

MASS::bandwidth.nrd(z))/6

else if (length(h) != 3)

h <- rep(h, length = 3)

if (length(n) != 3)

n <- rep(n, length = 3)

if (length(lims) == 2)

lims <- rep(lims, length = 6)

gx <- seq(lims[1], lims[2], length = n[1])

gy <- seq(lims[3], lims[4], length = n[2])

gz <- seq(lims[5], lims[6], length = n[3])

mx <- matrix(outer(gx, x, dnorm, h[1]), n[1], nx)

my <- matrix(outer(gy, y, dnorm, h[2]), n[2], nx)

mz <- matrix(outer(gz, z, dnorm, h[3]), n[3], nx)

v <- array(0, n)

tmy.nx <- t(my)/nx

for (k in 1:n[3]) {

tmy.nz.zk <- tmy.nx * mz[k, ]

v[, , k] <- mx %*% tmy.nz.zk

}

return(list(x = gx, y = gy, z = gz, d = v))

}

line_theme_default <-

function (font_use = "Arial")

{

library(ggplot2)

options(scipen = -1)

mytheme <- theme_bw() + theme(panel.grid = element_blank(),

panel.border = element_rect(color = "#000000", size = 0.80000000000000004),

axis.text = element_text(color = "#000000", size = 11),

axis.text.x = element_text(angle = 0, hjust = 0.5, vjust = 0.5),

axis.text.y = element_text(hjust = 1, vjust = 0.5), axis.title = element_text(color = "#000000",

size = 14, face = "plain"), axis.title.x = element_text(margin = margin(2.5,

0, 2.5, 0, "mm")), axis.title.y = element_text(margin = margin(0,

2.5, 0, 2.5, "mm")), axis.ticks = element_line(color = "#000000",

size = 0.5), axis.ticks.length = unit(0.10000000000000001,

"cm"), legend.title = element_blank(), plot.title = element_text(size = 16,

face = "plain", hjust = 0.5), plot.margin = unit(c(5,

5, 5, 5), "mm"))

if (font_use != "" & "extrafont" %in% installed.packages()) {

library(extrafont)

library(extrafontdb)

library(Rttf2pt1)

if (font_use %in% fonts()) {

mytheme <- mytheme + theme(text = element_text(family = font_use))

}

}

if (FALSE & font_use == "Arial" & "Cairo" %in% installed.packages()) {

library(Cairo)

CairoFonts(regular = "Arial:style=Regular", bold = "Arial:style=Bold",

italic = "Arial:style=Italic", bolditalic = "Arial:style=Bold Italic,BoldItalic",

)

source(ggsave_R)

}

if (FALSE & "showtext" %in% installed.packages()) {

library(showtext)

showtext_auto(enable = TRUE)

font.add("Arial", regular = paste0(ttf_dir, "/arial.ttf"),

bold = paste0(ttf_dir, "/arialbd.ttf"), italic = paste0(ttf_dir,

"/ariali.ttf"), bolditalic = paste0(ttf_dir,

"/arialbi_ttf"))

mytheme <- mytheme + theme(text = element_text(family = "Arial"))

}

mytheme

}

ListCellCluster <-

function (object, outfile = "Cells.cluster.list.xls", cluster = "seurat_clusters",

sample = "orig.ident", group = "Groups")

{

data <- .GetMetaData(object, cols = c(Cluster = cluster,

Samples = sample, Groups = group))

WriteTable(data, file = outfile)

}

ListMarker <-

function (object, object.markers, outfile = "DeGene.list.xls",

is.return = FALSE, is.fast = FALSE, group.by = "seurat_clusters",

assay = DefaultAssay(object), slot = "data", group.by.name = "Cluster",

...)

{

Targets_name <- paste0("Target_", group.by.name)

Others_name <- paste0("Other_", group.by.name)

Targets_mean_name <- paste0(Targets_name, "_mean")

Others_mean_name <- paste0(Others_name, "_mean")

Targets_pct_name <- paste0(Targets_name, "_pct")

Others_pct_name <- paste0(Others_name, "_pct")

Targets_mean <- CalAvgExp(object, unique(object.markers$gene),

is.return = T, is.reverse = F, assay = assay, slot = slot,

group.by = group.by, ...) %>% reshape2::melt(varnames = c("gene",

"cluster"), value.name = Targets_mean_name) %>% mutate(cluster = factor(cluster))

Others_mean <- CalAvgExp(object, unique(object.markers$gene),

is.return = T, is.reverse = T, assay = assay, slot = slot,

group.by = group.by, ...) %>% reshape2::melt(varnames = c("gene",

"cluster"), value.name = Others_mean_name) %>% mutate(cluster = factor(cluster))

Name <- FindFeaturesName(object, unique(object.markers$gene),

"name", is.fast = is.fast)

marker_list <- object.markers %>% left_join(y = Targets_mean) %>%

left_join(y = Others_mean) %>% mutate(Log2FC = log2(!!as.name(Targets_mean_name)/!!as.name(Others_mean_name)),

name = Name[gene]) %>% select(`:=`(!!Targets_name, cluster),

`Gene ID` = gene, `Gene Name` = name, `:=`(!!Targets_pct_name,

pct.1), `:=`(!!Others_pct_name, pct.2), !!Targets_mean_name,

!!Others_mean_name, Log2FC, Pvalue = p_val, Qvalue = p_val_adj)

if (is.null(outfile) || is.return) {

return(marker_list)

}

else {

marker_list[["Gene ID"]] <- ChangeOUTName(marker_list[["Gene ID"]],

object@misc$fdata)

WriteTable(marker_list, outfile)

}

}

Load <-

function (file)

{

object <- readRDX(file)

if ("version" %in% slotNames(object)) {

if (grepl("^2", object@version)) {

object <- Seurat::UpdateSeuratObject(object)

}

}

return(object)

}

MakeSeuratObj <-

function (parameter = list(), assay = "RNA", data_name = parameter$data$name,

data_dir = parameter$data$dir, name_list = parameter$name_list,

group.use = parameter$Groups, use.names = FALSE)

{

object.list <- list()

for (i in seq(data_name)) {

mat <- .Read10X(data_dir[i], use.names = use.names, assay = assay)

object.list[[i]] <- CreateSeuratObject(counts = mat,

project = data_name[i], assay = assay)

}

object <- MergeObject(object.list)

object <- SetSeuratInfo(object, data_name = data_name, name_list = name_list,

group.use = group.use)

return(object)

}

MakeSeuratObj_ARC <-

function (parameter = list(), assay = "RNA", use.names = FALSE,

assay.ATAC = "ATAC", do.merge_peak = TRUE, data_name = parameter$data$name,

data_dir = parameter$data$dir, data_frag = parameter$data$fragment,

data_meta = parameter$data$metadata, name_list = parameter$name_list,

group.use = parameter$Groups, gtf_file = parameter$ref$gtf)

{

object.list <- list()

for (i in seq(data_name)) {

counts <- .Read10X(data_dir[i], use.names = use.names)

object.list[[i]] <- CreateSeuratObject(counts = counts[[assay]],

project = data_name[i], assay = assay)

object.list[[i]][[assay.ATAC]] <- CreateChromatinAssay(counts = counts[[assay.ATAC]],

fragments = data_frag[i], sep = c(":", "-(?=\\d+$)"),

min.cells = -1, min.features = -1)

metadata <- read.csv(data_meta[i], header = TRUE, row.names = 1,

stringsAsFactors = FALSE)

metadata <- metadata[, c("is_cell", "atac_peak_region_fragments",

"atac_fragments")]

metadata <- metadata[colnames(object.list[[i]]), ]

object.list[[i]] <- AddMetaData(object.list[[i]], metadata = metadata)

}

if (do.merge_peak) {

object.list <- AddMergePeaks(object.list, assay.name = assay.ATAC)

}

object <- MergeObject(object.list)

object <- SetSeuratInfo(object, data_name = data_name, name_list = name_list,

group.use = group.use)

if (!is.null(gtf_file)) {

gtf <- rtracklayer::import(con = gtf_file)

if (!"gene_biotype" %in% names(gtf@elementMetadata@listData)) {

col <- grep("gene_type|biotype", names(gtf@elementMetadata@listData),

value = T)[1]

gtf$gene_biotype <- gtf@elementMetadata@listData[[col]]

}

Annotation(object[[assay.ATAC]]) <- gtf

}

DefaultAssay(object) <- assay

return(object)

}

MakeSpatialObj <-

function (parameter = list(), assay = "Spatial", filter.matrix = TRUE,

data_name = parameter$data$name, data_dir = parameter$data$dir,

name_list = parameter$name_list, group.use = parameter$Groups,

refdir = parameter$refdir, use.names = FALSE, image.dirname = "spatial",

mat.dirname = "filtered_feature_bc_matrix", mat.h5name = "filtered_feature_bc_matrix.h5")

{

object.list <- list()

for (i in seq(data_name)) {

input <- file.path(data_dir[i], mat.dirname)

if (!dir.exists(input))

input <- file.path(data_dir[i], mat.h5name)

mat <- .Read10X(input, use.names = use.names, assay = assay)

object.list[[i]] <- CreateSeuratObject(counts = mat,

project = data_name[i], assay = assay)

image <- Read10X_Image(image.dir = file.path(data_dir[i],

image.dirname), filter.matrix = filter.matrix)

if (all(grepl(pattern = "-[0-9]+$", x = rownames(image@coordinates)))) {

rownames(image@coordinates) <- as.vector(x = as.character(x = sapply(X = rownames(image@coordinates),

FUN = Seurat:::ExtractField, field = 1, delim = "-")))

}

image <- image[Cells(x = object.list[[i]])]

DefaultAssay(image) <- assay

object.list[[i]][[data_name[i]]] <- image

}

object <- MergeObject(object.list)

names(object@images) <- data_name

if (is.null(name_list) && !is.null(refdir))

name_list <- file.path(refdir, "/genes/name_list.xls")

object <- SetSeuratInfo(object, data_name = data_name, name_list = name_list,

group.use = group.use, assay = assay)

return(object)

}

MergeObject <-

function (object.list, data_name = do.call(c, lapply(object.list,

function(x) levels(x@meta.data$orig.ident))))

{

if (class(object.list) == "list") {

if (length(object.list) == 1) {

object <- RenameCells(object.list[[1]], add.cell.id = data_name)

return(object)

}

else {

object <- merge(object.list[[1]], object.list[-1],

add.cell.ids = data_name)

return(object)

}

}

else {

return(object.list)

}

}

Otsu <-

function (data, bin = 256, is.return.range = TRUE)

{

dt0 <- bin_stat(data, bin = bin)

dt <- table(dt0)

dt[dt > 100] <- 100

if (length(dt) > 0) {

thres <- core_otsu(dt)

}

else {

thres <- 0

}

if (is.return.range)

return(range(as.numeric(names(dt0)[dt0 %in% names(dt)[dt >=

thres]])))

else return(as.numeric(names(dt)[dt == thres - 1]))

}

Otsu.bak <-

function (data, digits = 0, is.return.range = TRUE)

{

dt0 <- table(round(data, digits = digits))

dt <- dt0

dt[dt > 100] <- 100

thres <- core_otsu(dt)

if (is.return.range)

return(range(as.numeric(names(dt0)[dt0 >= thres])))

else return(thres)

}

OverrideFeatures <-

function (object, parameter = list(), assay = NULL, slots = c("counts",

"data"), features = parameter$filter_gene$override$features,

set_to = parameter$filter_gene$override$set_to)

{

if (is.null(assay))

assay <- DefaultAssay(object)

if (is.null(set_to))

set_to <- 0

if (!is.null(features)) {

features_tmp <- NULL

for (i in features) {

if (file.exists(i)) {

features_tmp <- c(features_tmp, read.table(i,

sep = "\t", quote = "")[1])

}

else {

features_tmp <- features[i]

}

}

features <- FindFeaturesID(object, features_tmp)

for (slot in slots) {

feature <- intersect(features, rownames(slot(object@assays[[assay]],

slot)))

slot(object@assays[[assay]], slot)[feature, ] <- set_to

}

if (!is.null(object@misc$counts)) {

feature <- intersect(features, rownames(object@misc$counts))

object@misc$counts[feature, ] <- set_to

}

}

return(object)

}

PerformDE <-

function (object, cells.1, cells.2, features, test.use, verbose,

min.cells.feature, latent.vars, densify, ...)

{

if (!(test.use %in% DEmethods_latent()) && !is.null(x = latent.vars)) {

warning("'latent.vars' is only used for the following tests: ",

paste(DEmethods_latent(), collapse = ", "), call. = FALSE,

immediate. = TRUE)

}

if (!test.use %in% DEmethods_checkdots()) {

CheckDots(...)

}

if (length(intersect(cells.1, cells.2)) > 0) {

common.cells <- intersect(cells.1, cells.2)

newname.cells <- paste0(common.cells, "_new")

cells.2 <- setdiff(cells.2, cells.1)

object <- object[, c(cells.1, cells.2, common.cells),

drop = FALSE]

colnames(object) <- c(cells.1, cells.2, newname.cells)

cells.2 <- c(cells.2, newname.cells)

}

data.use <- object[features, c(cells.1, cells.2), drop = FALSE]

if (densify) {

data.use <- as.matrix(x = data.use)

}

de.results <- switch(EXPR = test.use, wilcox = WilcoxDETest(data.use = data.use,

cells.1 = cells.1, cells.2 = cells.2, verbose = verbose,

...), bimod = DiffExpTest(data.use = data.use, cells.1 = cells.1,

cells.2 = cells.2, verbose = verbose), roc = MarkerTest(data.use = data.use,

cells.1 = cells.1, cells.2 = cells.2, verbose = verbose),

t = DiffTTest(data.use = data.use, cells.1 = cells.1,

cells.2 = cells.2, verbose = verbose), negbinom = GLMDETest(data.use = data.use,

cells.1 = cells.1, cells.2 = cells.2, min.cells = min.cells.feature,

latent.vars = latent.vars, test.use = test.use, verbose = verbose),

poisson = GLMDETest(data.use = data.use, cells.1 = cells.1,

cells.2 = cells.2, min.cells = min.cells.feature,

latent.vars = latent.vars, test.use = test.use, verbose = verbose),

MAST = MASTDETest(data.use = data.use, cells.1 = cells.1,

cells.2 = cells.2, latent.vars = latent.vars, verbose = verbose,

...), DESeq2 = DESeq2DETest(data.use = data.use,

cells.1 = cells.1, cells.2 = cells.2, verbose = verbose,

...), LR = LRDETest(data.use = data.use, cells.1 = cells.1,

cells.2 = cells.2, latent.vars = latent.vars, verbose = verbose),

stop("Unknown test: ", test.use))

return(de.results)

}

pie_theme_default <-

function (font_use = "Arial")

{

library(ggplot2)

mytheme <- theme_void() + theme(legend.title = element_blank(),

legend.key.height = unit(5, "mm"), legend.key.width = unit(5,

"mm"), strip.text = element_text(size = 16, face = "plain",

hjust = 0.5), plot.title = element_text(size = 20,

face = "plain", hjust = 0.5), )

if (font_use != "" & "extrafont" %in% installed.packages()) {

library(extrafont)

library(extrafontdb)

library(Rttf2pt1)

if (font_use %in% fonts()) {

mytheme <- mytheme + theme(text = element_text(family = font_use))

}

}

if (FALSE & font_use == "Arial" & "Cairo" %in% installed.packages()) {

library(Cairo)

CairoFonts(regular = "Arial:style=Regular", bold = "Arial:style=Bold",

italic = "Arial:style=Italic", bolditalic = "Arial:style=Bold Italic,BoldItalic")

source(ggsave_R)

}

if (FALSE & "showtext" %in% installed.packages()) {

library(showtext)

showtext_auto(enable = TRUE)

font_add("Arial", regular = arial_ttf)

mytheme <- mytheme + theme(text = element_text(family = "Arial"))

}

mytheme

}

PlotAboutFeatures <-

function (object, features = NULL, group.by = "seurat_clusters",

outpref = NULL, group.colors = NULL, plot.feature = FALSE,

is.use.name = TRUE, reduction = "umap", ...)

{

object@meta.data <- droplevels(object@meta.data)

PlotDotPlot(object, features = features, group.by = group.by,

outfile = paste0(outpref, ".DotPlot.pdf"), is.use.name = is.use.name)

PlotHeatmapPlot(object, features = features, group.by = group.by,

outfile = paste0(outpref, ".Heatmap.pdf"), group.colors = group.colors,

is.use.name = is.use.name, ...)

if (plot.feature) {

PlotFeaturePlot(object, features = features, outfile = paste0(outpref,

".Distribution.pdf"), reduction = reduction, is.use.name = is.use.name)

}

}

PlotBasicStat <-

function (object, outpref = NULL, color = NULL, assay = NULL,

group.by = "orig.ident", span = NULL, stat_mito = TRUE, stat_plastid = TRUE,

standard = NULL, ...)

{

if (is.null(color)) {

color <- switch(group.by, Groups = object@misc[["color.group"]],

orig.ident = object@misc[["color.sample"]], seurat_clusters = object@misc[["color.cluster"]])

}

if (is.null(assay)) {

assay <- DefaultAssay(object)

}

nFeature <- paste0("nFeature_", assay)

nCount <- paste0("nCount_", assay)

filter_feature <- function(features) unlist(sapply(features,

function(x) if (x %in% colnames(object@meta.data))

x))

get_y_name <- function(feature) sapply(features, switch,

percent.mito = "Percentage(%)", percent.plastid = "Percentage(%)",

"Number")

features <- c(nFeature, nCount)

if (stat_mito)

features <- c(features, "percent.mito")

if (stat_plastid)

features <- c(features, "percent.plastid")

features <- filter_feature(features)

VlnplotShell(object, features = features, labs.y = get_y_name(features),

outfile = paste0(outpref, ".merge.pdf"), cols.use = color,

group.by = group.by, assay = assay, standard = standard,

...)

features <- c("expected.marker", "exclude.marker")

features <- filter_feature(features)

VlnplotShell(object, features = features, labs.y = get_y_name(features),

outfile = paste0(outpref, ".PresetMarker.pdf"), cols.use = color,

group.by = group.by, assay = assay, ...)

FeatureScatterShell(object, feature1 = nCount, feature2 = nFeature,

span = span, outfile = paste0(outpref, ".nCount-nFeature.pdf"),

cols = color, group.by = group.by, standard = standard)

if (stat_mito && exists("percent.mito", object@meta.data)) {

FeatureScatterShell(object, feature1 = nCount, feature2 = "percent.mito",

span = span, outfile = paste0(outpref, ".nCount-pMito.pdf"),

cols = color, group.by = group.by, standard = standard)

}

if (stat_plastid && exists("percent.plastid", object@meta.data)) {

FeatureScatterShell(object, feature1 = nCount, feature2 = "percent.plastid",

span = span, outfile = paste0(outpref, ".nCount-pPlastid.pdf"),

cols = color, group.by = group.by, standard = standard)

}

}

PlotCluster <-

function (object, reduction = "umap", p1.group.by = "orig.ident",

split.by = p1.group.by, p2.group.by = "seurat_clusters",

outpref = NULL, ...)

{

.PlotCluster(object, reduction = reduction, p1.group.by = p1.group.by,

p2.group.by = p2.group.by, outfile = paste0(outpref,

".pdf"), ...)

for (i in unique(object@meta.data[[split.by]])) {

cells.use <- rownames(object@meta.data)[object@meta.data[[split.by]] ==

i]

.PlotCluster(object, reduction = reduction, cells = cells.use,

p1.group.by = p1.group.by, p2.group.by = p2.group.by,

outfile = paste0(outpref, ".", i, ".pdf"), ...)

}

data <- object[[reduction]]@cell.embeddings %>% as.data.frame() %>%

tibble::rownames_to_column(var = "Cells") %>% left_join(.GetMetaData(object,

cols = c(Samples = p1.group.by, Cluster = p2.group.by,

"Groups")))

WriteTable(data, file = paste0(outpref, ".plot.data.tmp"))

}

PlotDensityPlot <-

function (object, features = NULL, reduction = "umap", outpref = "DensityPlot/",

...)

{

if (is.null(features)) {

.PlotDensityPlot(object, reduction = reduction, outpref = outpref,

...)

}

else {

for (i in features) {

.PlotDensityPlot(object, i, reduction = reduction,

outpref = outpref, ...)

}

}

}

PlotDotPlot <-

function (object, features = NULL, outfile = NULL, group.by = "seurat_clusters",

is.use.name = TRUE, ...)

{

p <- DotPlot(object, features = features, group.by = group.by,

...) + RotatedAxis()

p <- p + dot_theme_default() + theme(axis.text.x = element_text(angle = 45,

hjust = 1, vjust = 1), axis.text.y = element_text(hjust = 1))

if (is.use.name) {

levels(p$data$features.plot) <- FindFeaturesName(object,

levels(p$data$features.plot))

}

if (is.null(outfile)) {

return(p)

}

else {

w <- max(6, ceiling(length(features)) * 0.34999999999999998 +

2)

h <- max(6, length(unique(object@meta.data[[group.by]])) *

0.40000000000000002)

ggsave(p, file = outfile, width = w, height = h, limitsize = FALSE)

}

}

PlotFeaturePlot <-

function (object, features, reduction = "umap", is.combine = TRUE,

outpref = NULL, outfile = NULL, is.use.name = TRUE, ...)

{

if (is.combine) {

.PlotFeaturePlot(object, features = features, reduction = reduction,

outfile = outfile, ...)

}

else {

if (is.null(outpref))

outpref <- "ExpPlot"

for (i in features) {

if (is.use.name) {

name <- FindFeaturesName(object, i)

name <- gsub("[ /\\]", "_", name)

name <- gsub("%20", "_", name)

}

else {

name = i

}

.PlotFeaturePlot(object, features = i, reduction = reduction,

outfile = paste(c(outpref, name, "pdf"), collapse = "."),

plot.basic.size = 6, ...)

}

}

}

PlotHeatmap.online <-

function (object, feature = NULL, parameter = list(), outfile = NULL,

annot_with = parameter$annotation_col$with, annot_by = parameter$annotation_col$by,

orders = parameter$annotation_col$order, filter_list = parameter$data$filter,

assay = NULL, slot = "scale.data", legend.position = IfNull(parameter$legend$position,

"right"), color = IfNull(unlist(parameter$legend$color[c("low",

"mid", "high")]), c("#A020F0", "#000000", "#FFFF00")),

title = IfNull(parameter$labs$title, NA), xlab = parameter$labs$xlab,

ylab = parameter$labs$ylab, scale.range = IfNull(parameter$data$scale.range,

3), scale.min = scale.range * -1, scale.max = scale.range,

cluster_rows = IfNull(parameter$hclust$row, TRUE), cluster_cols = FALSE,

show_rownames = IfNull(parameter$axis$y.text$show, TRUE),

show_colnames = IfNull(parameter$axis$x.text$show, FALSE),

is.useGeneName = IfNull(parameter$data$use.genename, TRUE),

is.useIdName = IfNull(parameter$data$use.id_name, FALSE),

fontsize = IfNull(parameter$font$size, 10), font_family = parameter$font$family,

...)

{

cells <- Cells(object)

for (i in names(filter_list)) {

colname <- switch(i, cluster = "seurat_clusters", sample = "orig.ident",

group = "group")

if (!is.null(filter_list[[i]]) && exists(colname, object@meta.data)) {

cells <- setdiff(cells, Cells(object)[object@meta.data[[colname]] %in%

filter_list[[i]]])

}

}

annotation_row <- NA

annotation_col <- object@meta.data[cells, c("seurat_clusters",

"orig.ident")]

colnames(annotation_col) <- c("cluster", "sample")

if (exists("group", object@meta.data))

annotation_col$group <- object@meta.data[cells, "group"]

if (!is.null(annot_with))

annotation_col <- annotation_col[intersect(annot_with,

colnames(annotation_col))]

order_col <- union(annot_by, annot_with)

if (!is.null(orders)) {

for (i in names(orders)) {

if (i %in% colnames(annotation_col)) {

level <- levels(annotation_col[[i]])

level <- c(intersect(orders[[i]], level), setdiff(level,

orders[[i]]))

annotation_col[[i]] <- factor(annotation_col[[i]],

levels = level)

if (!is.null(orders[[i]])) {

annotation_col <- annotation_col[annotation_col[[i]] %in%

orders[[i]], ]

}

}

}

}

annotation_col <- droplevels(annotation_col)

annotation_col <- tibble::rownames_to_column(annotation_col,

var = "cell") %>% arrange_at(vars(!!!lapply(order_col,

as.name))) %>% tibble::column_to_rownames(var = "cell")

annotation_colors <- sapply(colnames(annotation_col), function(x) switch(x,

sample = object@misc[["color.sample"]], cluster = object@misc[["color.cluster"]],

group = object@misc[["color.group"]])[levels(annotation_col[[x]])],

simplify = FALSE)

annotation_colors <- annotation_colors[!sapply(annotation_colors,

is.null)]

is.legend <- if (legend.position == "none")

FALSE

else TRUE

color <- colorRampPalette(color)(100)

mat <- GetAssayData(object, assay = assay, slot = slot)

if (slot == "scale.data" && nrow(mat) == 0) {

object <- ScaleData(object, assay = assay, features = rownames(object))

mat <- GetAssayData(object, assay = assay, slot = slot)

}

if (!is.null(feature)) {

if (!is.null(object@misc$fdata)) {

feature <- FindFeaturesID(object, feature)

}

if (!all(feature %in% rownames(mat))) {

object <- ScaleData(object, assay = assay, features = feature)

mat <- GetAssayData(object, assay = assay, slot = slot)

}

mat <- mat[as.character(feature), , drop = FALSE]

if (nrow(mat) < 2)

cluster_rows <- FALSE

}

mat <- mat[, rownames(annotation_col), drop = FALSE]

mat <- MinMax(mat, scale.min, scale.max)

breaks <- if (length(unique(as.vector(mat))) == 1) {

unique(as.vector(mat)) + c(-1, 0, 1)

}

else {

NA

}

if (is.useIdName) {

name <- FindFeaturesName(object, rownames(mat), col = "name")

name <- paste0(rownames(mat), "(", name, ")")

rownames(mat) <- name

}

else {

if (is.useGeneName)

rownames(mat) <- FindFeaturesName(object, rownames(mat),

"name")

}

require(grid)

ph <- pheatmap::pheatmap(mat, useRaster = T, border_color = NA,

breaks = breaks, color = color, legend = is.legend, clustering_method = "ward.D2",

treeheight_row = 20, cluster_rows = cluster_rows, cluster_cols = cluster_cols,

annotation_row = annotation_row, annotation_col = annotation_col,

annotation_colors = annotation_colors, show_rownames = show_rownames,

show_colnames = show_colnames, main = title, fontsize = fontsize,

slient = TRUE)

height <- max(7, convertHeight(sum(ph$gtable$heights), "inches",

valueOnly = T))

width <- max(7, convertWidth(sum(ph$gtable$widths), "inches",

valueOnly = T))

while (!is.null(dev.list())) {

dev.off()

}

if (!is.null(outfile)) {

pdf(file = outfile, height = height, width = width)

}

if (!is.null(xlab) || !is.null(ylab)) {

setHook("grid.newpage", function() pushViewport(viewport(x = 1,

y = 1, width = 0.90000000000000002, height = 0.90000000000000002,

name = "vp", just = c("right", "top"))), action = "prepend")

}

grid.draw(ph$gtable)

if (!is.null(xlab) || !is.null(ylab)) {

setHook("grid.newpage", NULL, "replace")

grid.text(xlab, y = -0.050000000000000003, gp = gpar(fontsize = fontsize))

grid.text(ylab, x = -0.050000000000000003, rot = 90,

gp = gpar(fontsize = fontsize))

}

if (!is.null(outfile)) {

dev.off()

}

}

PlotHeatmapPlot <-

function (object, features = NULL, group.by = "seurat_clusters",

is.use.name = TRUE, outfile = NULL, group.colors = NULL,

color = c("#FF00FF", "#000000", "#FFFF00"), slot = "scale.data",

assay = NULL, ...)

{

if (is.null(group.colors)) {

group.colors <- switch(group.by, seurat_clusters = object@misc$color.cluster,

orig.ident = object@misc$color.sample, Groups = object@misc$color.group)

}

else {

group.colors <- group.colors[levels(object@meta.data[[group.by]])]

}

if (slot == "scale.data") {

if (!is.null(features)) {

if (is.null(assay))

assay <- DefaultAssay(object)

DefaultAssay(object) <- assay

if (!all(features %in% rownames(slot(object@assays[[assay]],

slot)))) {

message("Below features arenot in scale.data, recal scale.data:")

message(paste0(features[!features %in% rownames(slot(object@assays[[assay]],

slot))], collapse = ", "))

object <- ScaleData(object, features = features)

}

}

}

p <- DoHeatmap(object = object, features = features, cells = NULL,

group.by = group.by, group.colors = group.colors, combine = FALSE,

raster = FALSE, slot = slot, assay = assay, ...)

p <- p[[1]]

p <- p + theme(legend.title = element_blank())

p$layers[[2]] <- NULL

if (is.use.name) {

levels(p$data$Feature) <- FindFeaturesName(object, levels(p$data$Feature))

}

if (length(color) == 2) {

p <- p + scale_fill_gradient(low = color[1], high = color[2],

na.value = "white")

}

else if (length(color) == 3) {

p <- p + scale_fill_gradient2(low = color[1], mid = color[2],

high = color[3], na.value = "white")

}

else if (length(color) > 3) {

p <- p + scale_fill_gradientn(colors = color, na.value = "white")

}

if (is.null(outfile)) {

return(p)

}

else {

h <- max(7, length(unique(features)) * 0.11 + 2.5)

w <- h * 4/3

p <- p + theme(plot.margin = margin(t = 1, r = 1, unit = "lines"))

ggsave(p, file = outfile, width = w, height = h, limitsize = FALSE)

}

}

PlotPresetMarker <-

function (object, cols.use = object@misc$color.cluster, group.by = "seurat_clusters",

outpref = "PresetMarker", reduction = "umap")

{

PresetMarker <- union(object@misc[["expected.marker"]], object@misc[["more.marker"]])

PresetMarker <- union(PresetMarker, object@misc[["exclude.marker"]])

if (length(PresetMarker)) {

VlnplotShell(object, features = PresetMarker, outfile = paste0(outpref,

".VlnPlot.pdf"), titles = object@misc$fdata[PresetMarker,

"merge_name"], cols.use = cols.use, group.by = group.by)

PlotAboutFeatures(object, features = PresetMarker, outpref = outpref,

plot.feature = TRUE, group.by = group.by, reduction = reduction)

}

}

PlotVlnPlot <-

function (object, features, outpref = NULL, group.by = "seurat_clusters",

cols.use = NULL, is.use.name = TRUE, ...)

{

name <- if (is.use.name) {

FindFeaturesName(object, features)

}

else if (is.null(names(features))) {

features

}

else {

names(features)

}

names(features) <- as.vector(name)

if (is.null(cols.use)) {

cols.use <- switch(group.by, seurat_clusters = object@misc$color.cluster,

orig.ident = object@misc$color.sample)

}

p <- VlnplotShell(object, features, split.save.pref = outpref,

group.by = group.by, titles = name, cols.use = cols.use,

...)

}

readRDX <-

function (file)

{

con <- gzfile(file)

on.exit(close(con))

magic <- readChar(con, 5L, useBytes = TRUE)

if (grepl("RD[ABX][2-9]\n", magic)) {

object <- get(load(file))

}

else {

object <- readRDS(file)

}

return(object)

}

RenameFeatures <-

function (object, new.names = NULL, from.type = c("id", "merge_name",

"old_merge_name"), to.type = c("id", "merge_name", "old_merge_name"),

features = NULL)

{

if (is.null(new.names)) {

from.type <- match.arg(from.type)

to.type <- match.arg(to.type)

fdata <- object@misc$fdata

fdata$id <- rownames(fdata)

new.names <- fdata[, to.type]

names(new.names) <- fdata[, from.type]

}

if (is.null(features)) {

assays <- Seurat:::FilterObjects(object = object, classes.keep = "Assay")

for (assay in assays) {

slot(object = object, name = "assays")[[assay]] <- RenameFeatures.Assays(object = object[[assay]],

new.names = new.names)

}

dimreducs <- Seurat:::FilterObjects(object = object,

classes.keep = "DimReduc")

for (dr in dimreducs) {

object[[dr]] <- RenameFeatures.DimReduc(object = object[[dr]],

new.names = new.names)

}

return(object)

}

else {

return(as.vector(new.names[features]))

}

}

RenameFeatures.Assays <-

function (object, new.names = NULL)

{

for (data.slot in c("counts", "data", "scale.data")) {

old.data <- GetAssayData(object = object, slot = data.slot)

if (nrow(x = old.data) <= 1) {

next

}

old.name <- rownames(x = slot(object = object, name = data.slot))

rownames(x = slot(object = object, name = data.slot)) <- as.vector(new.names[old.name])

}

if (length(slot(object = object, name = "var.features")) >

0) {

old.name <- rownames(x = slot(object = object, name = "var.features"))

slot(object = object, name = "var.features") <- as.vector(new.names[old.name])

}

return(object)

}

RenameFeatures.DimReduc <-

function (object, new.names = NULL)

{

for (projected in c(TRUE, FALSE)) {

data.slot <- ifelse(projected, "feature.loadings.projected",

"feature.loadings")

old.data <- Loadings(object = object, projected = projected)

rownames(x = old.data) <- as.vector(new.names[rownames(x = old.data)])

slot(object = object, name = data.slot) <- old.data

}

return(object)

}

RestoreObject <-

function (object)

{

if (!is.null(object@misc[["counts"]]) && ncol(object@misc[["counts"]]) !=

ncol(object)) {

metadata <- if (exists("pdata", object@misc)) {

object@misc[["pdata"]]

}

else {

object@meta.data

}

new.object <- CreateSeuratObject(counts = object@misc[["counts"]],

meta.data = metadata, project = object@project.name,

assay = "RNA")

new.object@misc <- object@misc

return(new.object)

}

else {

return(object)

}

}

SetColor <-

function (x, type = "tsne", tag = "set1", ...)

{

if (!is.factor(x))

x <- as.factor(x)

color <- if (exists("fetch_color"))

fetch_color(n = nlevels(x), type = type, tag = tag, ...)

else rainbow(n = nlevels(x))

names(color) <- levels(x)

return(color)

}

SetSeuratInfo <-

function (object, data_name = NULL, name_list = NULL, sample_col = "orig.ident",

assay = "RNA", group.use = NULL, group_col = "Groups")

{

object@meta.data[[sample_col]] <- if (is.null(data_name)) {

as.factor(object@meta.data[[sample_col]])

}

else {

factor(object@meta.data[[sample_col]], levels = data_name)

}

object <- SetIdent(object, value = sample_col)

object@misc[["fdata"]] <- AddFData(object, name_list)

object@misc[["pdata"]] <- FetchData(object, c(sample_col,

paste0("nFeature_", assay), paste0("nCount_", assay)))

object@misc[["counts"]] <- GetAssayData(object, slot = "counts",

assay = assay)

object@misc[["color.sample"]] <- SetColor(object@meta.data[[sample_col]],

"tsne", "set3")

if (!is.null(group.use)) {

object@meta.data[[group_col]] <- object@meta.data[[sample_col]]

group <- unlist(lapply(names(group.use), function(i) {

x <- rep(i, length.out = length(group.use[[i]]))

names(x) <- group.use[[i]]

x

}))

levels(object@meta.data[[group_col]]) <- group[levels(object@meta.data[[group_col]])]

object@meta.data[[group_col]] <- factor(object@meta.data[[group_col]],

levels = names(group.use))

object@misc[["pdata"]][[group_col]] <- object@meta.data[[group_col]]

object@misc[["color.group"]] <- SetColor(object@meta.data[[group_col]],

"tsne", "set2")

}

return(object)

}

show_all_color <-

function ()

{

library(dplyr, warn.conflicts = F)

dt <- reshape2::melt(color.list)

dt <- dt %>% group_by(L1, L2, L3) %>% mutate(x = 1:n()) %>%

arrange(L1, L2, as.numeric(L3), x)

dt$L3 <- factor(dt$L3, levels = as.character(0:max(as.numeric(dt$L3))))

cc <- unique(as.character(dt$value))

names(cc) <- cc

library(ggplot2, warn.conflicts = F)

p <- list()

for (i in unique(dt$L1)) {

p[[i]] <- ggplot(dt %>% filter(L1 == i), aes(x = x, y = L3)) +

geom_tile(aes(fill = value), color = "grey") + facet_grid(L2 ~

., scales = "free_y", switch = "y") + scale_fill_manual(values = cc) +

scale_x_continuous(expand = expand_scale()) + ylab(i) +

theme_minimal() + theme(legend.position = "none",

strip.placement = "outside", panel.grid = element_blank(),

axis.title.x = element_blank(), axis.text.x = element_blank())

}

cowplot::plot_grid(plotlist = p, ncol = 1, axis = "ltrb",

align = "hv")

}

SplitObject.Image <-

function (objects, names.in = "orig.ident")

{

if (class(objects) != "list") {

if ("images" %in% slotNames(objects) && length(objects@images) >

1) {

keep.image <- levels(droplevels(objects@meta.data)[[names.in]])

objects@images <- objects@images[keep.image]

}

}

else {

for (i in seq(objects)) {

if ("images" %in% slotNames(objects[[i]]) && length(objects[[i]]@images) >

1) {

keep.image <- levels(droplevels(objects[[i]]@meta.data)[[names.in]])

objects[[i]]@images <- objects[[i]]@images[keep.image]

}

}

}

return(objects)

}

StatCluster <-

function (object, group.by = "orig.ident", outpref = "Cluster.stat",

stat.what = "seurat_clusters", assay = DefaultAssay(object),

...)

{

.StatCluster(object, stat.what = stat.what, outpref = outpref,

assay = assay)

.StatCluster_by(object, stat.what = stat.what, group.by = group.by,

outpref = outpref)

.PlotClusterStat(object, stat.what = stat.what, group.by = group.by,

outpref = outpref, ...)

}

StatFeatures <-

function (object, features = NULL, col.name = NULL, stat_pct = FALSE,

assay = NULL, add_to_pdata = FALSE)

{

if (is.null(features) || length(features) == 0) {

warning("[features] is empty. return 'object' without any change.")

return(object)

}

if (file.exists(features[1]))

features <- read.table(features[1], sep = "\t", quote = "")[[1]]

features <- FindFeaturesID(object = object, features = features,

unlist = FALSE)

if (is.null(assay))

assay <- DefaultAssay(object = object)

features <- intersect(features, rownames(object[[assay]]))

metadata <- Matrix::colSums(x = GetAssayData(object = object,

slot = "counts", assay = assay)[features, , drop = FALSE])

if (stat_pct)

metadata <- metadata/object@meta.data[[paste0("nCount_",

assay)]] * 100

if (!is.null(x = col.name)) {

object@misc[[col.name]] <- features

if (add_to_pdata)

object@misc$pdata[[col.name]] <- metadata

object <- AddMetaData(object = object, metadata = metadata,

col.name = col.name)

return(object)

}

else {

return(metadata)

}

}

StatFilterCells <-

function (object, group.by = "orig.ident", outfile = "Filter.stat.xls",

out_celllist = "filtered_cells.xls", old_metadata = object@misc$pdata,

current_metadata = object@meta.data)

{

filter_cells <- setdiff(rownames(old_metadata), rownames(current_metadata))

write.table(filter_cells, file = out_celllist, quote = F,

sep = "\t", col.name = F)

name <- if (group.by == "orig.ident")

"Samples"

else group.by

group.by <- as.name(group.by)

a <- current_metadata %>% group_by(`:=`(!!name, !!group.by)) %>%

summarise(after_filter_num = n(), after_filter_median_UMI_per_cell = median(nCount_RNA),

after_filter_median_genes_per_cell = median(nFeature_RNA))

b <- old_metadata %>% group_by(`:=`(!!name, !!group.by)) %>%

summarise(before_filter_num = n(), before_filter_median_UMI_per_cell = median(nCount_RNA),

before_filter_median_genes_per_cell = median(nFeature_RNA))

if (exists("percent.mito", current_metadata) && exists("percent.mito",

old_metadata)) {

a <- current_metadata %>% group_by(`:=`(!!name, !!group.by)) %>%

summarise(after_filter_median_MT_per_cell = median(percent.mito)) %>%

left_join(y = a)

b <- old_metadata %>% group_by(`:=`(!!name, !!group.by)) %>%

summarise(before_filter_median_MT_per_cell = median(percent.mito)) %>%

left_join(y = b)

}

if (exists("percent.plastid", current_metadata) && exists("percent.plastid",

old_metadata)) {

a <- current_metadata %>% group_by(`:=`(!!name, !!group.by)) %>%

summarise(after_filter_median_Plastid_per_cell = median(percent.plastid)) %>%

left_join(y = a)

b <- old_metadata %>% group_by(`:=`(!!name, !!group.by)) %>%

summarise(before_filter_median_Plastid_per_cell = median(percent.plastid)) %>%

left_join(y = b)

}

filter_stat_table <- full_join(b, a) %>% replace(is.na(.),

0) %>% mutate(pct = paste0(round(after_filter_num/before_filter_num *

100, 2), "%")) %>% select(!!name, before_filter_num,

after_filter_num, pct, contains("UMI"), contains("genes"),

contains("MT"), contains("Plastid"))

write.table(filter_stat_table, file = outfile, quote = F,

sep = "\t", row.names = F)

}

StatFilterCells_ATAC <-

function (object, group.by = "orig.ident", outfile = "Filter.ATAC.stat.xls",

out_celllist = "filtered_cells.xls", old_metadata = object@misc$pdata,

current_metadata = object@meta.data)

{

filter_cells <- setdiff(rownames(old_metadata), rownames(current_metadata))

write.table(filter_cells, file = out_celllist, quote = F,

sep = "\t", col.name = F)

name <- if (group.by == "orig.ident")

"Samples"

else group.by

group.by <- as.name(group.by)

a <- current_metadata %>% group_by(`:=`(!!name, !!group.by)) %>%

summarise(after_filter_num = n(), after_filter_median_UMI_per_cell = median(nCount_RNA),

after_filter_median_genes_per_cell = median(nFeature_RNA))

b <- old_metadata %>% group_by(`:=`(!!name, !!group.by)) %>%

summarise(before_filter_num = n(), before_filter_median_UMI_per_cell = median(nCount_RNA),

before_filter_median_genes_per_cell = median(nFeature_RNA))

filter_stat_table <- left_join(a, b) %>% mutate(pct = paste0(round(after_filter_num/before_filter_num *

100, 2), "%")) %>% select(!!name, before_filter_num,

after_filter_num, pct, before_filter_median_UMI_per_cell,

after_filter_median_UMI_per_cell, before_filter_median_genes_per_cell,

after_filter_median_genes_per_cell)

}

StatMarker <-

function (object.markers, Cluster_name = "Cluster", Item_name = "Number of DE genes",

color = NULL, outpref = "DeGene.stat")

{

stat <- cbind(tibble(`:=`(!!Cluster_name, Item_name)) %>%

as.matrix(), t(table(object.markers$cluster)))

WriteTable(stat, paste0(outpref, ".xls"))

p <- ggplot(object.markers) + geom_bar(aes(x = cluster, fill = cluster),

stat = "count") + theme_light() + labs(x = Cluster_name,

y = Item_name)

p <- p + bar_theme_default()

p <- p + theme(legend.position = "none")

if (!is.null(color))

p <- p + scale_fill_manual(values = color)

ggsave(p, file = paste0(outpref, ".pdf"), height = 6, width = 8)

invisible(stat)

}

SubsetObj <-

function (object, cells = NULL, sample = NULL, cluster = NULL,

sample.name = "orig.ident", cluster.name = "seurat_clusters",

...)

{

cells <- if (!is.null(cells))

cells

else Cells(object)

if (!is.null(sample)) {

if (is.list(sample)) {

}

else {

cells.sample <- Cells(object)[object[[sample.name]][[1]] %in%

sample]

}

cells <- intersect(cells, cells.sample)

}

if (!is.null(cluster)) {

if (is.list(cluster)) {

}

else {

cells.cluster <- Cells(object)[object[[cluster.name]][[1]] %in%

cluster]

}

cells <- intersect(cells, cells.cluster)

}

others <- list(...)

if (length(others) > 0) {

}

object <- object[, cells]

object@meta.data <- droplevels(object@meta.data)

if ("images" %in% slotNames(object)) {

object@images <- object@images[Images(object) %in% unique(object@meta.data[[sample.name]])]

for (image in Images(object)) {

image.cells <- intersect(rownames(object@images[[image]]@coordinates),

cells)

object@images[[image]]@coordinates <- object@images[[image]]@coordinates[image.cells,

]

}

}

return(object)

}

ttf_dir <-

"src/Rlib/../../fonts/tff/msttcore"

unlist.rev <-

function (data)

{

data <- unlist(lapply(names(data), function(x) `names<-`(x = rep(x,

length(data[[x]])), value = data[[x]])))

return(data)

}

VlnplotShell <-

function (object, features = NULL, group.by = "orig.ident", cols.use = NULL,

group.point.by = NULL, group.point.color = NULL, labs.y = NULL,

titles = NULL, legend.position = "none", outfile = NULL,

split.save.pref = NULL, pt.size = 0.10000000000000001, alpha = 0.5,

is.cline.x.text = TRUE, standard = NULL, nRow = NULL, ...)

{

if (is.null(features))

return(1)

if (is.null(nRow)) {

nCol <- ifelse(length(features) == 3, 3, ceiling(sqrt(length(features))))

nRow <- ceiling(length(features)/nCol)

}

else {

nCol <- ceiling(length(features)/nRow)

}

plots <- VlnPlot(object = object, features = features, ncol = nCol,

group.by = group.by, cols = cols.use, pt.size = pt.size,

combine = FALSE, ...)

for (i in seq(features)) {

if (!is.null(group.point.by) && pt.size > 0) {

data <- cbind(plots[[i]]$data, object[[group.point.by]])

plots[[i]]$layers[[2]] <- NULL

plots[[i]] <- plots[[i]] + geom_jitter(aes_string(color = group.point.by),

height = 0, size = pt.size, data = data)

if (!is.null(group.point.color)) {

plots[[i]] <- plots[[i]] + scale_color_manual(values = group.point.color)

}

}

if (group.by == "orig.ident")

plots[[i]] <- plots[[i]] + xlab("Samples")

else if (group.by == "Groups")

plots[[i]] <- plots[[i]] + xlab("Groups")

else if (group.by == "seurat_clusters")

plots[[i]] <- plots[[i]] + xlab("Clusters")

if (!is.null(labs.y[i]) && !is.na(labs.y[i]))

plots[[i]] <- plots[[i]] + ylab(labs.y[i])

if (!is.null(titles[i]) && !is.na(titles[i]))

plots[[i]] <- plots[[i]] + ggtitle(titles[i])

plots[[i]]$layers[[1]]$aes_params$alpha <- alpha

plots[[i]]$layers <- rev(plots[[i]]$layers)

plots[[i]] <- plots[[i]] + box_theme_default()

if (is.cline.x.text)

plots[[i]] <- plots[[i]] + theme(axis.text.x = element_text(angle = 45,

hjust = 1, vjust = 1))

if (!is.null(standard[[features[[i]]]])) {

plots[[i]] <- plots[[i]] + geom_hline(yintercept = standard[[features[[i]]]],

color = "red")

}

}

if (!is.null(split.save.pref)) {

for (i in seq(features)) {

name <- ifelse(!is.null(names(features[i])), names(features[i]),

features[i])

name <- gsub("[ /\\]", "_", name)

name <- gsub("%20", "_", name)

outfile.tmp <- paste(split.save.pref, name, "pdf",

sep = ".")

ggsave(plots[[i]] + theme(legend.position = legend.position),

file = outfile.tmp, width = 6, height = 6)

}

}

plots.combined <- patchwork::wrap_plots(plots, ncol = nCol) &

theme(legend.position = "none")

if (!is.null(dev.list()))

dev.off()

if (is.null(outfile)) {

return(plots.combined)

}

else {

ggsave(plots.combined, file = outfile, width = 6 * nCol,

height = 6 * nRow, limitsize = F)

}

}

WriteTable <-

function (x, file, quote = FALSE, sep = "\t", row.names = FALSE,

col.names = TRUE, ...)

{

write.table(x, file = file, quote = quote, sep = sep, row.names = row.names,

col.names = col.names, ...)

### Deal arguements

args <- commandArgs(T)

file <- args[1]

outdir <- args[2]

add_lib <- args[3]

if ( is.null( file ) | is.na( file ) ){

warning( "\n Usage : Seurat.R <parameter.yaml> (<outdir>)\n" )

quit()

}

### Loading Library

handlers <- list("bool#no" = function(x){if ( x %in% c("false", "FALSE") ) FALSE else x}, "bool#yes" = function(x){if ( x %in% c("true", "TRUE") ) TRUE else x})

parameter <- yaml::yaml.load_file( file, handlers = handlers)

library(Seurat)

library(dplyr)

library(ggplot2)

library(patchwork)

library(harmony)

library(future)

options(future.globals.maxSize = 100 * 1024 * 1024^2)

plan("multiprocess", workers = 4)

#plan("sequential")

#source("/home/xushuyang/Pipeline/R/SCellWare/Seurat_lib.R", chdir = T)

if ( ! is.na(add_lib) ) source(add_lib, chdir = T)

### Let's shake it

if ( ! is.na(outdir) ) {

dir.create(outdir, showWarnings = F, recursive = T)

setwd(outdir)

}

PlotAboutFeatures <- function(object, features = NULL, group.by = "seurat_clusters", outpref = NULL, group.colors = NULL, plot.feature = FALSE, is.use.name = TRUE, ... ) {

object@meta.data <- droplevels(object@meta.data)

PlotDotPlot(object, features = features, group.by = group.by, outfile = paste0(outpref, ".DotPlot.pdf"), is.use.name = is.use.name)

PlotHeatmapPlot(object, features = features, group.by = group.by, outfile = paste0(outpref, ".Heatmap.pdf"), group.colors = group.colors, is.use.name = is.use.name, ...)

if ( plot.feature ) {

PlotFeaturePlot(object, features = features, outfile = paste0(outpref, ".TSNE.Distribution.pdf"), reduction = "tsne", is.use.name = is.use.name)

PlotFeaturePlot(object, features = features, outfile = paste0(outpref, ".UMAP.Distribution.pdf"), reduction = "umap", is.use.name = is.use.name)

}

}

PlotPresetMarker <- function(object, cols.use = object@misc$color.cluster, group.by = "seurat_clusters", outpref = "PresetMarker"){

PresetMarker <- union(object@misc[["expected.marker"]], object@misc[["more.marker"]])

PresetMarker <- union(PresetMarker, object@misc[["exclude.marker"]])

if ( length(PresetMarker) ) {

VlnplotShell(object, features = PresetMarker, outfile = paste0(outpref, ".VlnPlot.pdf"), titles = object@misc$fdata[PresetMarker, "merge_name"], cols.use = cols.use, group.by = group.by)

PlotAboutFeatures(object, features = PresetMarker, outpref = outpref, plot.feature = TRUE, group.by = group.by)

}

}

### Creat Seurat Object

message( "==>Reading 10x data<==" )

if ( ! is.null(parameter$obj_use) ) {

message("Loading existed object ...")

load(parameter$obj_use)

if ( ! is.null(parameter$marker$expected) ) obj <- StatFeatures(obj, parameter$marker$expected, col.name = "expected.marker")

} else {

obj <- MakeSeuratObj(parameter)

### Add some check flag

message( "==>Adding MetaData<==" )

if ( ! is.null(parameter$marker$mito_list) ) obj <- StatFeatures(obj, parameter$marker$mito_list, col.name = "percent.mito", stat_pct = T, add_to_pdata = T)

if ( ! is.null(parameter$marker$expected) ) obj <- StatFeatures(obj, parameter$marker$expected, col.name = "expected.marker")

if ( ! is.null(parameter$marker$excluded) ) obj <- StatFeatures(obj, parameter$marker$excluded, col.name = "exclude.marker")

if ( ! is.null(parameter$marker$more) ) obj@misc[["more.marker"]] <- FindFeaturesID(obj, parameter$marker$more, unlist = FALSE)

WriteTable(tibble::rownames_to_column(obj@meta.data, var = "Cells"), file = "metadata.xls")

### Data Stat - before filter

message( "==>Stat before BasicInfo<==" )

if ( is.null(parameter$filter$filter.cells) ) {

PlotBasicStat(obj, "BasicInfo")

if ( ! is.null(parameter$Groups) ) {

PlotBasicStat(obj, "BasicInfo.groups", group.by = "Groups")

}

} else {

obj[["DF"]] <- "Singlet"

filter.cells <- readLines(parameter$filter$filter.cells)

obj@meta.data[filter.cells, "DF"] <- "Doublet"

PlotBasicStat(obj, "BasicInfo", group.point.by = "DF", group.point.color = c("Singlet" = "black", "Doublet" = "red"))

if ( ! is.null(parameter$Groups) ) {

PlotBasicStat(obj, "BasicInfo.groups", group.by = "Groups", group.point.by = "DF", group.point.color = c("Singlet" = "black", "Doublet" = "red"))

}

}

### Filter

message( "==>Filter<==" )

obj <- FilterGenes(obj, parameter)

obj <- FilterCells(obj, parameter, do.stat = FALSE)

StatFilterCells(obj, group.by = "orig.ident", outfile = "Filter.stat.xls")

if ( ! is.null(parameter$Groups) ) {

StatFilterCells(obj, group.by = "Groups", outfile = "Filter.stat.groups.xls")

}

### Data Stat - after filter

message( "==>Stat after BasicInfo<==" )

PlotBasicStat(obj, "AfterFilter.BasicInfo")

if ( ! is.null(parameter$Groups) ) {

PlotBasicStat(obj, "AfterFilter.BasicInfo.groups", group.by = "Groups")

}

}

if (parameter$reanalysis) {

### Normalization Data

message( "==>Normalization Data<==" )

obj <- DoNormalization(obj, parameter, is_SCTransform = FALSE)

### Reduce dimension

message( "==>Reduce dimension<==" )

obj <- DoDimReduc(obj)

### Find clusters

message( "==>Find clusters<==" )

obj <- DoFindClusters(obj, reduction = "pca", dims = NULL, resolution = parameter$cluster_resolution)

if ( ! is.null(parameter$integration$method) ) {

if ( length(table(obj[["orig.ident"]])) > 1 ) {

## check before integration visualization

obj[["beforeInteg.cluster"]] <- Idents(object = obj)

PlotCluster(obj, reduction = 'umap_RNA', outpref = "UMAP_before" )

PlotCluster(obj, reduction = 'tsne_RNA', outpref = "tSNE_before" )

if ( ! is.null(parameter$Groups) ) {

PlotCluster(obj, reduction = 'umap_RNA', outpref = "UMAP_before.groups", split.by = "Groups", p1.group.by = "Groups" )

PlotCluster(obj, reduction = 'tsne_RNA', outpref = "tSNE_before.groups", split.by = "Groups", p1.group.by = "Groups" )

}

### Integration

message( "==> Do Integration <==" )

if ( parameter$integration$method == "CCA" ) {

obj <- DoIntegration(obj, split.by = "orig.ident")

obj <- DoDimReduc(obj)

obj <- DoFindClusters(obj, reduction = "pca", resolution = parameter$cluster_resolution)

} else {

obj <- RunHarmony(obj, group.by.vars = "orig.ident", project.dim = FALSE, assay.use = DefaultAssay(obj))

obj <- DoDimReduc(obj, reduction = "harmony", reduction.surfix = "harmony")

obj <- DoFindClusters(obj, reduction = "harmony", resolution = parameter$cluster_resolution)

}

}

}

}

## Draw t-SNE plot

message( "==>Draw t-SNE plot<==" )

PlotCluster(obj, reduction = 'umap', outpref = "UMAP", p2.group.by = "seurat_clusters2", p2.color = obj@misc$color.cluster2, p2.label = F )

PlotCluster(obj, reduction = 'tsne', outpref = "tSNE", p2.group.by = "seurat_clusters2", p2.color = obj@misc$color.cluster2, p2.label = F )

if ( ! is.null(parameter$Groups) ) {

PlotCluster(obj, reduction = 'umap', outpref = "UMAP.groups", split.by = "Groups", p1.group.by = "Groups" )

PlotCluster(obj, reduction = 'tsne', outpref = "tSNE.groups", split.by = "Groups", p1.group.by = "Groups" )

}

### Save data object

#message( "==>Output obj.Rda<==" )

DefaultAssay(obj) <- "RNA"

#save(obj, file = "obj.Rda")

### stat table

message( "==>Stat table<==" )

StatCluster(obj)

if ( ! is.null(parameter$Groups) ) {

StatCluster(obj, "Groups")

}

CalAvgExp(obj)

ListCellCluster(obj)

PlotPresetMarker(obj)

#message( "==>Output obj.Rda<==" )

#save(obj, file = "obj.Rda")

### Find maker genes

message( "==>Find maker genes<==" )

obj.markers <- DoFindAllMarkers(obj, parameter)

message( "==>Output markers.Rda<==" )

save( obj.markers, file = "markers.Rda" )

#obj.markers$gene <- ChangeOUTName(obj.markers$gene, object@misc$fdata)

## stat marker

message( "==>stat marker<==" )

StatMarker(obj.markers, color = obj@misc$color.cluster)

ListMarker(obj, obj.markers)

### Top marker

message( "==>display top markers<==" )

top <- FindTopMarker(obj.markers, top_num = parameter$heatmap$top, object = obj)

PlotAboutFeatures(obj, features = unique(top$gene), outpref = "Top")

dir.create("DensityPlot/", showWarnings = F, recursive = T)

unlink("DensityPlot/*", recursive = T)

PlotDensityPlot(obj, unique(top$gene), reduction = 'tsne', outpref = "DensityPlot/")

dir.create("ExpPlot/", showWarnings = F, recursive = T)

unlink("ExpPlot/*", recursive = T)

PlotFeaturePlot(obj, unique(top$gene), reduction = 'tsne', outpref = "ExpPlot/ExpPlot", is.combine = FALSE)

dir.create("ViolinPlot/", showWarnings = F, recursive = T)

unlink("ViolinPlot/*", recursive = T)

PlotVlnPlot(obj, unique(top$gene), outpref = "ViolinPlot/ViolinPlot")

### Hasta la vista, baby

message( "==>All Done!<==" )

library(Seurat)

library(dplyr)

library(ggplot2)

library(patchwork)

source("Seurat_lib.R", chdir = T)

PlotCluster = function(object, reduction = 'umap', p1.group.by = "orig.ident", split.by = p1.group.by, p2.group.by = "seurat_clusters", outpref = NULL, ...){

.PlotCluster(object, reduction = reduction, p1.group.by = p1.group.by, p2.group.by = p2.group.by, outfile = paste0(outpref, ".pdf"), ...)

.PlotCluster(object, reduction = reduction, p1.group.by = NULL, p2.group.by = p2.group.by, outfile = paste0(outpref, ".svg"), ...)

for ( i in unique(object@meta.data[[split.by]]) ){

cells.use <- rownames(object@meta.data)[object@meta.data[[split.by]] == i]

.PlotCluster(object, reduction = reduction, cells = cells.use, p1.group.by = p1.group.by, p2.group.by = p2.group.by, outfile = paste0(outpref, ".", i, ".pdf"), ...)

.PlotCluster(object, reduction = reduction, cells = cells.use, p1.group.by = NULL, p2.group.by = p2.group.by, outfile = paste0(outpref, ".", i, ".svg"), ...)

}

data <- object[[reduction]]@cell.embeddings %>% as.data.frame() %>%

tibble::rownames_to_column(var = "Cells") %>%

left_join(.GetMetaData(object, cols = c("Samples" = p1.group.by, "Cluster" = p2.group.by, "Groups")))

WriteTable(data, file = paste0(outpref, ".plot.data.tmp"))

}

args = commandArgs(TRUE)

obj_file = args[1]

sample = args[2]

if (isTRUE(sample)) sample = "T"

obj = Load(obj_file)

obj$seurat_clusters_old = factor(obj$seurat_clusters_old, levels = stringr::str_sort(unique(obj$seurat_clusters_old), numeric = T))

obj@misc$color.cluster_old = SetColor(obj$seurat_clusters_old, "tsne", "set1")

PlotCluster(obj, reduction = 'umap', outpref = paste0("UMAP_celltype.", sample), p2.group.by = "seurat_clusters", p2.color = obj@misc$color.cluster, p2.label = F )

PlotCluster(obj, reduction = 'tsne', outpref = paste0("tSNE_celltype.", sample), p2.group.by = "seurat_clusters", p2.color = obj@misc$color.cluster, p2.label = F )

PlotCluster(obj, reduction = 'umap', outpref = paste0("UMAP_cluster.", sample), p2.group.by = "seurat_clusters_old", p2.color = obj@misc$color.cluster_old, p2.label = T)

PlotCluster(obj, reduction = 'tsne', outpref = paste0("tSNE_cluster.", sample), p2.group.by = "seurat_clusters_old", p2.color = obj@misc$color.cluster_old, p2.label = T)

#source('~/SeuratLoad.R')

library(Seurat)

library(dplyr)

library(ggplot2)

library(patchwork)

library(harmony)

library(stringr)

source("Seurat_lib.R", chdir = T)

load('obj.Rda')

#load('markers.Rda')

genes = read.table("gene.list", header = F, stringsAsFactors = F)$V1

genes = str_to_title(genes)

#top <- FindTopMarker(obj.markers, top_num = 5, object = obj)

features = FindFeaturesID(obj, genes)

#features = top$gene

obj = subset(obj, seurat_clusters == "Other", invert = TRUE)

obj@meta.data = droplevels(obj@meta.data)

#top = FindTopMarker(obj.markers, top_num = 5, object = obj)

obj$seurat_clusters = as.character(obj$seurat_clusters) %>% factor(levels = rev(levels(obj$seurat_clusters)))

obj$seurat_clusters_old = as.character(obj$seurat_clusters_old)

cl = setNames(obj$seurat_clusters, obj$seurat_clusters_old)

cl = cl[unique(names(cl))]

cl = aggregate(names(cl), by = list(cl), as.character)

cl$x = lapply(cl$x, str_sort, numeric = T, decreasing = T)

cl = do.call(c, cl$x)

obj$seurat_clusters_old = factor(obj$seurat_clusters_old, levels = cl)

p = PlotDotPlot(obj, features = rev(features), group.by = "seurat_clusters")

levels(p$data$features.plot) = FindFeaturesName(obj, levels(p$data$features.plot))

p = p + scale_color_gradientn(colours=c("#432060","#198B8A","#E9DD29"))+ labs(x = '', y = '')+

theme_bw() +

theme(panel.grid = element_blank(),

panel.border = element_rect(linetype = 1, fill = NA),

axis.text.x = element_text(angle = 90, vjust = .5, hjust = 1))

ggsave('Marker.dotplot.pdf', p, w = 8, h = 4, bg = 'white')

ggsave('Marker.dotplot.png', p, w = 8, h = 4, bg = 'white')

library(ggplot2)

library(dplyr)

library(patchwork)

library(reshape2)

#ct = c(T_diff = "T", B_diff = "B", NK_diff = "NK", Mye = "Mye")

#cp = c("Control-vs-TB", "TB_3-vs-TB")

ct = c("Naive_CD4+T", "Th1/17", "Tfh", "Treg", "Naive_CD8+T", "Cytotoxic_CD8+T", "Exhausted_CD8+T", "Proliferated_T")

ct = setNames(ct, ct)

names(ct) = gsub("\\/", "_", names(ct))

cp = c("Control-vs-CTB", "ATB-vs-CTB")

df = list()

for (i in names(ct)) {

for (j in cp) {

idx = paste0(i, ".", j)

df[[idx]] = read.table(paste0(idx, ".gsea.xls"), sep = "\t", header = F, stringsAsFactors = F)

colnames(df[[idx]]) = c("Pathway", "KEGG_ClassA", "KEGG_ClassB", "NES", "Pvalue", "Qvalue")

df[[idx]]$Cluster = ct[i]

df[[idx]]$Comparison = j

}

}

df = do.call(rbind, df)

df$log10q = -log10(abs(df$Qvalue))

df$Comparison[df$Comparison == "Control-vs-TB"] = "Control-vs-CTB"

df$Comparison[df$Comparison == "TB_3-vs-TB"] = "ATB-vs-CTB"

df$Comparison = factor(df$Comparison, levels = c("Control-vs-CTB", "ATB-vs-CTB"))

df = df %>% group_by(KEGG_ClassA, KEGG_ClassB) %>% arrange(.by_group = TRUE)

df$Pathway = factor(df$Pathway, unique(df$Pathway))

df$KEGG_ClassA = factor(df$KEGG_ClassA, unique(df$KEGG_ClassA))

df$KEGG_ClassB = factor(df$KEGG_ClassB, unique(df$KEGG_ClassB))

df$Cluster = factor(df$Cluster, levels = ct)

breaks = seq(0, ceiling(max(df$log10q[!is.infinite(df$log10q)])), 0.5)

print(breaks)

limits = c(min(breaks), max(breaks))

p1 = ggplot(df) +

geom_point(aes(x = Cluster, y = Pathway, size = log10q, color = NES)) +

facet_wrap(~Comparison) +

scale_x_discrete(position = "top") +

scale_y_discrete(position = "right") +

scale_size_continuous(breaks = breaks, limits = limits) +

guides(size=guide_legend(nrow = 1)) +

scale_color_gradient2(low = "blue", mid = "white", high = "red") +

labs(x = "", y = "", size = "-log10(Qvalue)") +

theme_bw() +

theme(legend.position="top", legend.box="vertical", axis.text.x = element_text(angle = 90, hjust = 0, vjust = 1), axis.text.y = element_text(colour = "black"), strip.placement = "outside")

h = length(unique(df$Pathway)) * 0.175 + 1.5

w = length(unique(df$Cluster)) * length(cp) * 0.2 + 6.5

ggsave(p1, filename = "DotPlot.pdf", height = h, width = w)

df2 = df[, c("Pathway", "KEGG_ClassB", "KEGG_ClassA")] %>% summarize_all(unique) %>% as.data.frame()

rownames(df2) = df2$Pathway

df2 = df2[levels(p1$data$Pathway), ]

write.table(df2, "DotPlot.order.xls", col.names = T, row.names = F, sep = "\t", quote = F)

df$log10q[df$Qvalue > 0.05] = NA

p1 = ggplot(df) +

geom_point(aes(x = Cluster, y = Pathway, size = log10q, color = NES)) +

facet_wrap(~Comparison) +

scale_x_discrete(position = "top") +

scale_y_discrete(position = "right") +

scale_size_continuous(breaks = breaks, limits = limits) +

guides(size=guide_legend(nrow = 1)) +

scale_color_gradient2(low = "blue", mid = "white", high = "red") +

labs(x = "", y = "", size = "-log10(Qvalue)") +

theme_bw() +

theme(legend.position="top", legend.box="vertical", axis.text.x = element_text(angle = 90, hjust = 0, vjust = 1), axis.text.y = element_text(colour = "black"), strip.placement = "outside")

h = length(unique(df$Pathway)) * 0.175 + 1.5

w = length(unique(df$Cluster)) * length(cp) * 0.2 + 6.5

ggsave(p1, filename = "DotPlot2.pdf", height = h, width = w)

df2 = df[, c("Pathway", "KEGG_ClassB", "KEGG_ClassA")] %>% summarize_all(unique) %>% as.data.frame()

rownames(df2) = df2$Pathway

df2 = df2[levels(p1$data$Pathway), ]

write.table(df2, "DotPlot2.order.xls", col.names = T, row.names = F, sep = "\t", quote = F)

bin <- dirname(normalizePath(sub('--file=', '', grep('--file=', commandArgs(), value = T))))

if ( numeric_version(paste0(R.Version()[c("major","minor")], collapse = ".")) > 4.0 ) {

.libPaths("/home/xushuyang/R/x86_64-unknown-linux-gnu-library/4.0/")

}

getopt <- function(){

library(optparse)

option_list <- list(

make_option(c("-i", "--infile"), type = "character",

help = "required. Seurat object. not compatible with --expfile."),

make_option(c("-e", "--expfile"), type = "character",

help = "required. expression file. not compatible with --infile." ),

make_option(c("-o", "--outdir"), type = "character",

help = "required"),

make_option(c("-d", "--msigdb"), type = "character", default = paste0(bin, "/database/msigdb_v6.2.xml"),

help = 'optional, msigdb. default "%default". not compatible with --gmt'),

make_option(c("-t", "--gmt"), type = "character",

help = 'optional, self-made .gmt file. not compatible with --msigdb' ),

make_option(c("-m", "--method"), type = "character", default = "gsva",

help = 'optional, one of [ "gsva", "ssgsea", "zscore", "plage" ]. default "%default".' ),

make_option(c("-u", "--use.group"), action = "store_true", default = FALSE,

help = 'optional. calculate average exps by "--group.by" before running GSVA. work only with --infile.'),

make_option(c("-g", "--group.by"), type = "character", default = "seurat_clusters",

help = 'optional, default "%default". work only with --infile.'),

make_option(c("-l", "--add_lib"), type = "character",

help = 'optional')

)

opts.obj <- OptionParser(option_list = option_list)

opts <- parse_args(opts.obj)

if ( ( is.null(opts$infile) && is.null(opts$expfile) ) || is.null(opts$outdir) ) {

print_help(opts.obj)

q()

}

return(opts)

}

opts <- getopt()

##### load library #####

library("GSEABase")

library("GSVA")

library("Seurat")

source(paste0(bin, "/gsva_lib.R"), chdir = T)

if( !is.null(opts$add_lib) ) source(opts$add_lib, chdir = T)

##### _main_ ######

message("[Running] reading database ... " )

if ( is.null(opts$gmt) ) {

gsc <- getBroadSets(uri = opts$msigdb )

gsc.types <- sapply(gsc, function(elt) bcCategory(collectionType(elt)))

msigdb.type <- c( "h", "c1", "c2", "c3", "c4", "c5", "c6", "c7" )

names(msigdb.type) <- toupper(msigdb.type)

save.name <- "gsav.msigdb.Rda"

} else {

gsc <- getGmt(con = opts$gmt)

gsc.types <- names(gsc)

msigdb.type <- list(gsc.types)

names(msigdb.type) <- gsub(".gmt", "", basename(opts$gmt))

save.name <- paste("gsav", names(msigdb.type), "Rda", sep = ".")

}

gsc.geneid <- unique(unlist(geneIds(gsc)))

message("[Running] reading infile ... " )

if ( ! is.null(opts$infile) ) {

obj <- Load(opts$infile)

exp <- GetAssayData(obj)

if ( opts$use.group ) {

exp <- expm1(exp)

exp <- cal.mean(exp, obj@meta.data[[opts$group.by]])

}

exp.name <- as.vector(FindFeaturesName(obj, rownames(exp)))

if ( sum(exp.name %in% gsc.geneid) > sum(rownames(exp) %in% gsc.geneid) ) {

rownames(exp) <- exp.name

} else {

exp.name <- rownames(exp)

}

} else {

exp <- read.table(opts$expfile, header = TRUE, sep = "\t", check.name = FALSE, quote = '')

exp <- do.call(rbind, by(seq(nrow(exp)), exp[[1]], function(i) Matrix::colMeans(exp[i,-1]))) ## to remove rownames duplicate

exp.name <- rownames(exp)

}

message( "[Running] checking input's gene names ..." )

m1 <- CaseMatch(gsc.geneid, exp.name, F)

m2 <- CaseMatch(gsc.geneid, exp.name[!exp.name %in% m1], T)

if ( length(m2) > 0 ) {

m2_rev <- `names<-`(names(m2), m2)

rownames(exp)[exp.name %in% m2] <- as.vector(m2_rev[exp.name[exp.name %in% m2]])

}

message( " total gene names : ", length(exp.name) )

message( " in which match database : ", length(m1) )

message( " in which match database only if ignoring case: ", length(m2) )

message( " in which not match : ", length(exp.name) - length(m1) - length(m2) )

message( " they are : [[" )

message( " ", paste(exp.name[!exp.name %in% c(m1,m2)], collapse = ", " ) )

message( " ]]" )

message( "[Running] running gsva ..." )

gsva.list <- list()

for ( i in names(msigdb.type) ) {

gsva.list[[i]] <- gsva(exp, gsc[gsc.types %in% msigdb.type[[i]]], parallel.sz = 4, method = opts$method)

}

message( "[Running] plot results ..." )

for ( i in names(gsva.list) ) {

if ( ! is.null(opts$infile) ) {

if ( opts$use.group ) {

tmp <- gsva.list[[i]]

} else {

tmp <- cal.mean(gsva.list[[i]], obj@meta.data[[opts$group.by]])

}

}else {

tmp <- gsva.list[[i]]

}

plot.gsva(tmp, paste0( opts$outdir, "/", i, ".heatmap" ))

}

message( "[Running] output gsva matrix ..." )

for ( i in names(gsva.list) ) {

write.gsva(gsva.list[[i]], paste0( opts$outdir, "/", i, ".gsva.xls" ) )

}

message( "[Running] save gsva Rdata ... " )

save(gsva.list, file = paste0( opts$outdir, "/", save.name ) )

message( "[Finish] ALL DONE !" )

args = commandArgs(T)

library(Seurat)

library(dplyr)

library(ggplot2)

library(patchwork)

library(harmony)

library(stringr)

source("/public2/Bio/pipeline/SingleCell_Collections/SCellWare/v2.1/Seurat_lib.R", chdir = T)

#source('~/Start.r')

obj = Load(args[1])

dir.create(args[2])

setwd(args[2])

cell = data.frame(

Cells = Cells(obj),

Cluster = obj$seurat_clusters,

Samples = obj$orig.ident

)

sample = unique(cell$Samples)

color = obj@misc$color.cluster

#names(color) = NULL

#print(color)

s = lapply(sample, function(s) {

cells = cell[cell$Samples == s, ]

pdata = cells %>% group_by(Cluster) %>% summarise(Freq = length(Cells), .groups = 'drop')

# pdata = pdata[order(pdata$Freq),]

pdata$fraction = pdata$Freq / sum(pdata$Freq)

pdata$Cluster2 = paste0(pdata$Cluster, ' (', round(pdata$fraction * 100, digits = 1), '%)')

# pdata$Cluster = factor(pdata$Cluster, unique(pdata$Cluster))

# write.table(pdata, paste0(s, '.cluster.xls'), row.names = F, sep = '\t', quote = F)

pdata$ymax = cumsum(pdata$fraction)

pdata$ymin = c(0, pdata$ymax[-nrow(pdata)])

p = ggplot(pdata, aes(fill = Cluster, ymax = ymax, ymin = ymin, xmax = 3.5, xmin = 2, col = Cluster)) +

geom_rect() + coord_polar('y') + xlim(c(0, 4)) +

scale_fill_manual(values = color[levels(pdata$Cluster)]) + scale_color_manual(values = color[pdata$Cluster]) +

geom_text(aes(label = sub(' ', '\n', Cluster2),

x = 4, y = (ymax + ymin)/2), color = 'black', size = 4) +

geom_text(label = paste0(s, '\nTotal cells = ', nrow(cells)),

x = 0, y = 0, color = 'black', size = 5) +

labs(fill = 'Cluster', color = 'Cluster') +

theme_void() +

theme(panel.grid = element_blank(), axis.text = element_blank(), axis.ticks = element_blank())

ggsave(paste0(s, '.cluster.pdf'), p, w = 10, h = 8)

})

args = commandArgs(T)

obj_file = args[1]

outpfx = args[2]

use_cl = args[3]

.libPaths("/home/xushuyang/R/x86_64-unknown-linux-gnu-library/4.0/")

library(destiny)

library(Seurat)

library(ggplot2)

library(patchwork)

library(harmony)

library(dplyr)

source("/public2/Bio/pipeline/SingleCell_Collections/SCellWare/v2.1/Seurat_lib.R", chdir = T)

obj = Load(obj_file)

if (!is.na(use_cl)) {

keep_cl = strsplit(use_cl, ",") %>% unlist()

print(keep_cl)

obj = subset(obj, seurat_clusters %in% keep_cl)

print(table(obj$seurat_clusters))

obj@meta.data = droplevels(obj@meta.data)

obj@misc$color.cluster = obj@misc$color.cluster[levels(obj$seurat_clusters)]

}

embeddings <- obj@reductions$harmony@cell.embeddings

dm <- DiffusionMap(embeddings, k = ncol(embeddings))

dm2 = dm@eigenvectors

rownames(dm2) = rownames(embeddings)

obj[['diffusionmap']] = CreateDimReducObject(embeddings = dm2[Cells(obj), ], key="DC_")

outpref = paste0(outpfx, ".DiffusionMap")

.PlotCluster(obj, reduction = 'diffusionmap', p2.label = F, outfile = paste0(outpref, ".pdf"))

for (i in levels(obj$orig.ident)) {

obj2 = subset(obj, orig.ident == i)

obj2@meta.data = droplevels(obj2@meta.data)

obj2@misc$color.sample = obj@misc$color.sample[levels(obj2$orig.ident)]

.PlotCluster(obj2, reduction = 'diffusionmap', p2.label = F, outfile = paste0(outpref, ".", i, ".pdf"))

}

#col <- obj@meta.data[colnames(dm@transitions), "seurat_clusters"]

#pal <- obj@misc$color.cluster

## ggplot2å¯¹è±¡ï¼Œè‡ªå·±ä¿®æ”¹æ ·å¼

#p <- plot.DiffusionMap(dm, dims = 1:2, col = col, pal = pal)

#ggsave(p, filename = paste0(outpfx, ".diffusionmap.pdf"))

#col <- obj@meta.data[colnames(dm@transitions), "orig.ident"]

#pal <- obj@misc$color.sample

## ggplot2å¯¹è±¡ï¼Œè‡ªå·±ä¿®æ”¹æ ·å¼

#p <- plot.DiffusionMap(dm, dims = 1:2, col = col, pal = pal)

#ggsave(p, filename = paste0(outpfx, ".bySample.diffusionmap.pdf"))
